# Anatomy, feeding ecology, and ontogeny of a transitional baleen whale: a new genus and species of Eomysticetidae (Mammalia: Cetacea) from the Oligocene of New Zealand

**DOI:** 10.7717/peerj.1129

**Published:** 2015-09-10

**Authors:** Robert W. Boessenecker, R. Ewan Fordyce

**Affiliations:** 1 Department of Geology, University of Otago, Dunedin, New Zealand; 2 University of California Museum of Paleontology, University of California, Berkeley, CA, USA; 3 Current affiliation: Department of Geology and Environmental Geosciences, College of Charleston, Charleston, SC, USA

**Keywords:** Mysticeti, Eomysticetidae, New Zealand, Oligocene, Ontogeny, Filter feeding

## Abstract

The Eocene history of cetacean evolution is now represented by the expansive fossil record of archaeocetes elucidating major morphofunctional shifts relating to the land to sea transition, but the change from archaeocetes to modern cetaceans is poorly established. New fossil material of the recently recognized family Eomysticetidae from the upper Oligocene Otekaike Limestone includes a new genus and species, *Waharoa ruwhenua*, represented by skulls and partial skeletons of an adult, juvenile, and a smaller juvenile. Ontogenetic status is confirmed by osteohistology of ribs. *Waharoa ruwhenua* is characterized by an elongate and narrow rostrum which retains vestigial alveoli and alveolar grooves. Palatal foramina and sulci are present only on the posterior half of the palate. The nasals are elongate, and the bony nares are positioned far anteriorly. Enormous temporal fossae are present adjacent to an elongate and narrow intertemporal region with a sharp sagittal crest. The earbones are characterized by retaining inner and outer posterior pedicles, lacking fused posterior processes, and retaining a separate accessory ossicle. Phylogenetic analysis supports inclusion of *Waharoa ruwhenua* within a monophyletic Eomysticetidae as the earliest diverging clade of toothless mysticetes. This eomysticetid clade also included *Eomysticetus whitmorei*, *Micromysticetus rothauseni*, *Tohoraata raekohao*, *Tokarahia kauaeroa*, *Tokarahia lophocephalus*, and *Yamatocetus canaliculatus*. Detailed study of ontogenetic change demonstrates postnatal elaboration of the sagittal and nuchal crests, elongation of the intertemporal region, inflation of the zygomatic processes, and an extreme proportional increase in rostral length. Tympanic bullae are nearly full sized during early postnatal ontogeny indicating precocial development of auditory structures, but do increase slightly in size. Positive allometry of the rostrum suggests an ontogenetic change in feeding ecology, from neonatal suckling to a more specialized adult feeding behaviour. Possible absence of baleen anteriorly, a delicate temporomandibular joint with probable synovial capsule, non-laterally deflected coronoid process, and anteroposteriorly expanded palate suggests skim feeding as likely mode of adult feeding for zooplankton. Isotopic data in concert with preservation of young juveniles suggests the continental shelf of Zealandia was an important calving ground for latitudinally migrating Oligocene baleen whales.

## Introduction

**Figure 1:**
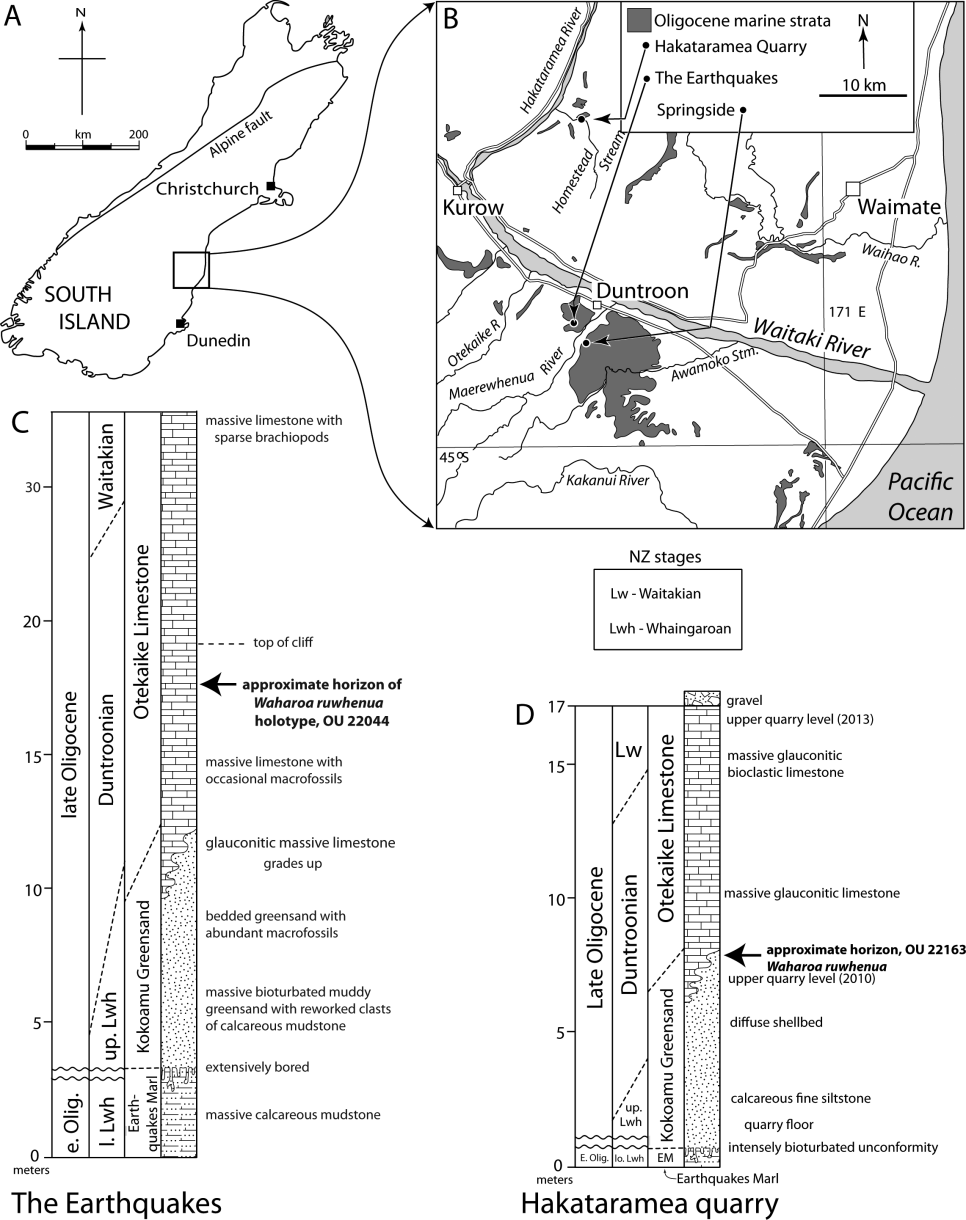
Geologic and geographic context of *Waharoa ruwhenua* localities. (A) map of South Island, New Zealand; (B) map of Waitaki Valley Regions showing location of Hakataramea Quarry, “The Earthquakes,” and Springside; (C), stratigraphic column of “The Earthquakes,” modified from Fordyce (1994); (D), stratigraphic column of Hakataramea Quarry, modified from (Gottfried, Fordyce & Rust, 2012).

The fossil record of cetaceans is densely sampled for the Neogene and many Miocene and Pliocene specimens provide good evidence of feeding adaptations (Muizon, Domning & Ketten, 2002; El Adli, Deméré & Boessenecker, 2014; Racicot et al., 2014), intraspecific variation (Bouetel & de Muizon, 2006; Gutstein et al., 2009; El Adli, Deméré & Boessenecker, 2014), adaptations for hearing and echolocation (Geisler & Luo, 1996; Luo & Eastman, 1995; Steeman, 2009), and pathology and disease (Dawson & Gottfried, 2002; Thomas et al., 2008; Beatty & Dooley, 2009; Gerholdt & Godfrey, 2010). Broader studies of fossil assemblages have elucidated cetacean paleobiogeography and faunal change (Barnes, 1977; Mchedlidze, 1984; Fordyce, 1991; Gottfried, Bohaska & Whitmore, 1994; Whitmore, 1994; Oishi & Hasegawa, 1995; Boessenecker, 2013), taphonomic patterns (Boessenecker, Perry & Schmitt, 2014) and megabiases (Uhen & Pyenson, 2007). Eocene cetacean assemblages now convincingly demonstrate the evolution of fully pelagic basilosaurid archaeocetes from earlier quadrupedal, fully terrestrial ancestors (Uhen, 2010). Spectacular fossils of some Eocene cetaceans such as *Dorudon atrox* Andrews, 1906 and *Georgiacetus vogtlensis* Hulbert et al., 1998 have yielded much information regarding locomotory adaptations and musculature, buoyancy, brain anatomy and evolution, feeding behavior, trophic relationships, hearing adaptations and ontogeny and skeletal growth (Hulbert et al., 1998; Uhen, 2004). Cetaceans from the Oligocene reveal the early evolutionary history of Neoceti, but most of the relevant literature consists of descriptive reports of ‘singleton’ specimens representing new taxa, but with little information on intraspecific variation, biogeography, biochronology, or ontogeny; indeed, the mode of life of Eocene archaeocetes is arguably better known than for Oligocene cetaceans (Sanders & Geisler, 2015). Ontogenetic changes in particular are generally poorly investigated amongst fossil cetaceans (but see observations in Kellogg, 1924; Kellogg, 1936; Bouetel & de Muizon, 2006; Uhen & Gingerich, 2001; Gingerich et al., 2009; Gutstein et al., 2009; Gol’din & Startsev, 2014), despite the promise for interpreting heterochrony (Tsai & Fordyce, 2014a) or potential for biasing cladistic analyses (Tsai & Fordyce, 2014b). In the absence of fossilized ontogenies, it may be possible to overinflate taxonomic diversity by erecting names upon different ontogenetic stages (Scannella & Horner, 2010; Campione et al., 2013). This paper reports a new genus and species of eomysticetid from the upper Oligocene Otekaike Limestone of New Zealand (Fig. 1) with three individuals represented by well-preserved crania, tympanoperiotics, and mandibles demonstrating ontogenetic changes from young juvenile to old adult, elucidating the early evolution of filter feeding in Chaeomysticeti, and representing one of the most completely known Oligocene cetaceans in terms of anatomy and paleobiology.

## Geologic Background

Fossils of *Waharoa ruwhenua* were collected from the Otekaike Limestone (Gage, 1957; Duntroonian to Waitakian Stages, Chattian, upper Oligocene) in North Otago and South Canterbury, South Island, New Zealand. Vertebrate skeletal remains from Otekaike Limestone and underlying Kokoamu Greensand include sharks, bony fish, marine birds (penguins and volant birds), and cetaceans; vertebrates most commonly occur as isolated bones but disarticulated associated and partially articulated skeletons occur infrequently (Fordyce, 1991; Boessenecker & Fordyce, 2015a). The glauconitic Kokoamu Greensand grades upwards into glauconitic limestone of the lower Maerewhenua Member of the Otekaike Limestone, deposited between 100 and 50 m water depth (Ayress, 1993). The Otekaike Limestone consists of massively bedded bioclastic limestone (packstone to grainstone, composed of sand-size equivalent foraminifera, bryozoan, mollusk, and echinoderm fragments) that is often glauconitic and probably deposited under transition zone settings below storm weather wave base under 50 m water depth, or possibly somewhat deeper offshore settings (Ayress, 1993; Beu & Maxwell, 1990); the general lack of mm to dm bedding suggests little traction current activity. The Otekaike Limestone has more-conspicuous macrofossils (albeit, generally not in shellbeds) than the underlying Kokoamu Greensand and includes sparse and isolated macroinvertebrates but some laterally extensive thin concentrations of presumed hiatal origin contain abundant brachiopods, bivalves, gastropods, scaphopods, and less abundant serpulids, barnacles, bryozoans, and scleractinian corals. Well-preserved calcareous microfossils such as foraminifera and ostracods are abundant. The Otekaike Limestone is conformably overlain by the Mt. Harris Formation (Waitakian-Otaian-Altonian Stage, Aquitanian-Burdigalian correlative, lower Miocene), but occasionally the contact is unconformable and marked by Gee Greensand (of Gage, 1957). The upper Kokoamu Greensand and lower Otekaike Limestone are correlative with the Duntroonian Stage (upper Chattian) and the uppermost Otekaike Limestone is assignable to the Waitakian Stage (uppermost Chattian and lower Aquitanian; Gage, 1957; Beu & Maxwell, 1990; see below).

## Methods

### Preparation, anatomical description, and illustration

Fossil material in OU collections was mechanically prepared with pneumatic air scribes. Fine preparation of earbones was performed under a Zeiss binocular microscope. Anatomical terminology follows that of Mead & Fordyce (2009) with additions from Oishi & Hasegawa (1995), Ekdale, Berta & Deméré (2011), and Boessenecker & Fordyce (2015b). Tympanoperiotic orientation follows Mead & Fordyce (2009) using anatomical structures (e.g., anterior process, posterior process, lateral tuberosity) to dictate orientation of tympanoperiotics when in isolation from the skull (in contrast to orientation *in situ*) to facilitate comparisons between taxa. Specimens were photographed with Nikon D90, D700, and D800 cameras with a 60 mm and 105 mm lens. For smaller specimens (e.g., tympanoperiotics) focus-stacking of several photographs (2–15) was implemented in Adobe Photoshop CS 6 to produce seamless images with continuous focus. Line illustrations have not been corrected for parallax.

### Osteohistology

Histologic sections were taken from rib fragments of *Waharoa* (OU 22075, 22163, 22044), embedded in epoxy and polished thin sections were prepared by University of Otago Petrology Technician B Pooley. Photomicrographs were captured under non-polarized light. Histological terminology follows Gray et al. (2007). Histological measurements were captured in ImageJ (Rasband, 1997–2015).

### Cladistic analysis

*Waharoa ruwhenua* was coded into the recent matrix published by Boessenecker & Fordyce (in press) consisting of 363 characters coded for 75 terminal taxa. Operational taxonomic units (OTUs) include three archaeocetes and two odontocetes as outgroups, and 69 mysticetes, including toothed mysticetes (*n* = 8), eomysticetids (*n* = 7), balaenids (*n* = 6), neobalaenids (*n* = 2), “cetotheres” *sensu lato* (*n* = 12), cetotheriids (*n* = 14), eschrichtiids (*n* = 3), and balaenopterids (*n* = 18). The three known specimens with cranial material of *Waharoa ruwhenua* were combined into a single OTU. Cladistic analysis was executed in TNT 1.1 (Goloboff, Farris & Nixon, 2008) using the “new technology” search with sectorial search and tree fusing options checked. Separate analyses were conducted under equal weights. Analyses included 10,000 random addition sequences and tree bisection-reconnection branch swapping saving 10 trees per replicate. The analysis is reported as a strict consensus tree with branch support (reported as GC frequency values) based on symmetric resampling with 2,000 replicates. See Boessenecker & Fordyce (in press).

### Nomenclatural acts

The electronic version of this article in Portable Document Format (PDF) will represent a published work according to the International Commission on Zoological Nomenclature (ICZN), and hence the new names contained in the electronic version are effectively published under that Code from the electronic edition alone. This published work and the nomenclatural acts it contains have been registered in ZooBank, the online registration system for the ICZN. The ZooBank LSIDs (Life Science Identifiers) can be resolved and the associated information viewed through any standard web browser by appending the LSID to the prefix “http://zoobank.org/”. The LSID for this publication is: urn:lsid:zoobank.org:pub:D7129183-9324-49AD-A8E2-9D0CC8FF8037. The online version of this work is archived and available from the following digital repositories: PeerJ, PubMed Central and CLOCKSS.

## Systematic Paleontology

**Table:** 

MAMMALIA Linneaus, 1758
CETACEA Brisson, 1762
MYSTICETI Gray, 1846
EOMYSTICETIDAE Sanders & Barnes, 2002
*Waharoa* new genus

**LSID**: urn:lsid:zoobank.org:act:3601D851-F9D2-4364-83F5-7A466DC432F3

**Type and only species**: *Waharoa ruwhenua*.

**Diagnosis of Genus**: same as for the type and only known species.

**Etymology**: Waharoa, meaning long mouth; from the Māori waha (mouth) plus roa (long).

*Waharoa ruwhenua* new species

**LSID**: urn:lsid:zoobank.org:act:2FC4AC75-2378-4FFA-801A-2A3475BFDF31

### Diagnosis of species

*Waharoa ruwhenua* is a relatively small-bodied mysticete (5–6 m estimated adult body length) with archaic features differing from crown Mysticeti including: an anteriorly placed bony nares, anteroposteriorly elongate and narrow intertemporal region, temporal fossae longer than wide, firmly closed naso-premaxilla and naso-frontal sutures, transversely broad median palatal keel, elongate and anteriorly directed zygomatic processes that extend anterior to the occipital shield apex, vertically developed falciform process of the squamosal, anteroposteriorly thickened paroccipital process with pit for stylohyal, trefoil-shaped occipital in posterior view, distinctly concave glenoid fossae of squamosal, unfused anterior and posterior pedicles of the tympanoperiotic, deep lateral pit on periotic, short posterior process of the periotic, well-differentiated lateral and medial lobes of tympanic bulla, horizontal cleft of sigmoid fissure, horizontal crest on posterior surface of medial lobe, tympanic cavity divided by transverse ridge, deeply incised elliptical foramen, double posterior pedicle of bulla, enlarged mandibular foramen and transversely thin “pan bone” of mandible, delicate angular process of the mandible, and vestigial alveoli in premaxilla, maxilla, and mandible. *Waharoa ruwhenua* differs from archaeocetes and toothed mysticetes in lacking alveoli from the posterior portion of the maxilla and possibly lacking adult dentition, possessing a proportionally more elongate rostrum, unfused fronto-maxilla and maxilla-premaxilla sutures, an orbitotemporal crest that is positioned entirely on the dorsal surface of the frontal, a subrectangular supraorbital process of the frontal that is transversely wider than anteroposteriorly long and at the same level as the nasals, posteroventrally oriented postglenoid processes of the squamosal, an occipital shield that extends anterior to the subtemporal crest, and posterolaterally directed paroccipital processes; differs from Basilosauridae in possessing nasal and premaxilla that extend further posterior than maxilla, nasals with parallel margins, vertical nuchal crests that do not dorsally overhang the posterior margin of the skull, transversely thickened basioccipital crests, pars cochlearis with rounded anteromedial margin and smoothly convex ventral surface, mallear fossa positioned medial to lateral tuberosity, superior process reduced to low ridge with anterior and posterior apices, posterior process of periotic that does not extend to lateral edge of braincase, anteriorly directed zygomatic processes, and humerus similar in length to ulna and radius; from Basilosauridae and Aetiocetidae in possessing a humerus with proximally positioned deltopectoral crest; from Basilosauridae and Balaenidae in possessing ulna and radius that are not anteriorly bowed or distally inflated; from Basilosauridae and Mammalodontidae in possessing a thin lateral edge of the maxilla, palatal foramina and sulci, lacking a firm mandibular symphysis, mandible with parallel dorsal and ventral margins; from Mammalodontidae and Aetiocetidae in more anterior termination of nasals, transversely narrower intertemporal region with high sagittal crest, and anteroposteriorly longer than wide temporal fossa; from Mammalodontidae and *Chonecetus* in possessing a transverse frontoparietal suture; from all archaeocetes and toothed mysticetes except *Chonecetus* in numerous supraorbital foramina and deep sulci in the frontal; and from *Aetiocetus* in lacking orbitotemporal crests that extend posteriorly onto the parietal.

*Waharoa ruwhenua* shares with Aetiocetidae and other Eomysticetidae an elongate posterior meatal crest that extends dorsally along most of the dorsoventral depth of the squamosal and an unfused mandibular symphysis. *Waharoa ruwhenua* shares with other Eomysticetidae various aforementioned archaic features and, to the exclusion of other Mysticeti, an extremely elongate rostrum and elongate nasals, an anteroposteriorly oriented and longitudinally twisted zygomatic process that lacks a supramastoid crest anterior to the subtemporal crest, a secondary squamosal fossa, and a superior process of the periotic reduced to a low discontinuous ridge with anterior and posterior apices. *Waharoa ruwhenua* shares with other Eomysticetidae and Cetotheriidae *sensu stricto* a transversely flattened and blade-like anterior process of the periotic. *Waharoa ruwhenua* shares *Tohoraata* and *Tokarahia* dorsoventrally shallow and wide occipital condyles, a triangular anterior process and well-developed incisural flange of the periotic; with *Tohoraata* and *Tokarahia* a concave anterodorsal margin of the anterior process of the periotic and a smooth and transversely convex posterior bullar facet; with *Tohoraata* a distinct lateral tubercle on the anterior process; and with *Eomysticetus* and *Micromysticetus* a short posterior process.

### Holotype

**Figure 2:**
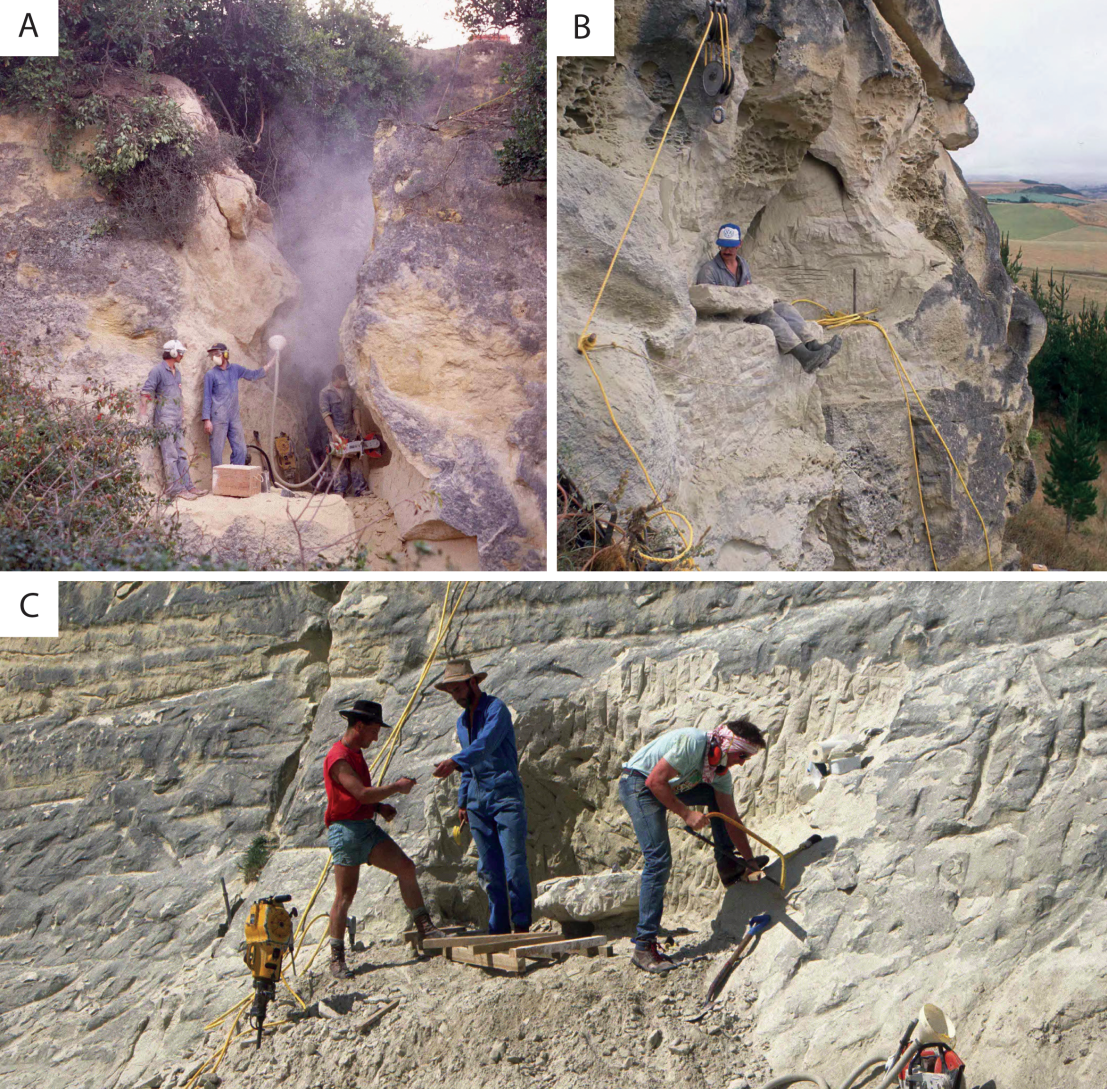
Excavation of *Waharoa ruwhenua* specimens. (A) “The Earthquakes,” OU 22044; (B) Springside, OU 22075; (C) Hakataramea Quarry, OU 22163. All photos © R. Ewan Fordyce.

OU 22044, nearly complete skull (approximate condylobasal length: 183 cm) with left tympanic bulla, right periotic, mandibles, atlas, axis, C5, C7, an isolated thoracic vertebra, ribs, and sternum. Collected from “The Earthquakes” (Fig. 2A), North Otago, during April, 1989, by RE Fordyce, A Grebneff, CM Jones, P McIntosh, and A Penniket (Grid reference I40:151931; New Zealand Fossil Record number I40/f94).

### Paratypes

OU 22075, small juvenile skeleton including anterior skull (estimated condylobasal length: 90 cm), squamosal fragments, tympanoperiotics, mandibles, axis, C7, six thoracic vertebrae including T1–T5, five lumbar vertebrae, one caudal vertebra, several ribs, scapula, humeri, radii, and ulnae, collected December 4–5 and 13–14 1990 from the lower Maerewhenua Member of the Otekaike Limestone exposed in west facing cliffs at Springside (Fig. 2B), North Otago, by RE Fordyce, A Grebneff, and CM Jones (Grid reference 141:228843; New Zealand Fossil Record number I41/f0184). OU 22163, large juvenile skeleton including nearly complete skull (estimated condylobasal length: 109.8 cm), tympanoperiotics, mandibles, four cervical vertebrae and poorly preserved thoracic vertebrae, and ribs, collected November 9–10 1992 from the lower Maerewhenua Member of the Otekaike Limestone at Hakataramea Quarry (Fig. 2C), South Canterbury, by RE Fordyce, CM Jones, RD Connell, and R Köhler (Grid reference I40:235137; New Zealand Fossil Record number I40/f0398).

### Referred specimen

OU 22140, isolated tympanic bulla collected October 10, 1990, from the lower Maerewhenua Member of the Otekaike Limestone at Awahokomo Pinnacles, Awahokomo Valley, North Otago, by A Grebneff (Grid reference I40:022085; New Zealand Fossil Record number I40/f0396).

### Etymology

Ruwhenua, from the Maori for shaking (ru) and whenua (land), a translation of the “The Earthquakes” locality.

### Type locality

“The Earthquakes,” 5 km east southeast of Duntroon, North Otago, South Island, New Zealand (Figs. 1 and 2A).

### Formation and age

The holotype was collected from a large (∼10 m) slumped block at “The Earthquakes” locality (Figs. 1 and 2A) near Duntroon (North Otago; New Zealand Fossil Record number I40/f94). Matrix samples from OU 22044 yielded no specimens of the Waitakian planktonic foraminiferan *Globoquadrina dehiscens*, suggesting Duntroonian age (27.3–25.2 Ma) for this specimen; likewise, the stratigraphic occurrence of this specimen within the Otekaike Limestone indicates a maximum age of Duntroonian. Also corroborating a maximum age of Duntroonian are valves of the pectinid *Lentipecten hochstetteri* (Beu & Maxwell, 1990). Taphonomic aspects of the holotype, including bite marks attributable to bony fish and elasmobranchs, bioerosion caused by the bone eating annelid worm *Osedax*, and evidence for fish (or elasmobranch) predation upon *Osedax*, were reported by Boessenecker & Fordyce (2015a).

OU 22163 was collected from the lower Otekaike Limestone at a quarry informally known as “Haughs’ Quarry” (Figs. 1 and 2C) in the Hakataramea River valley (South Canterbury; New Zealand Fossil Record number I40/f0398); this specimen occurred within a glauconitic limestone horizon corresponding to the uppermost parts of the Kokoamu Greensand-Otekaike Limestone transition, and approximately 4 m below the first appearance datum of *Globoquadrina dehiscens* within the quarry (D de B Hornibrook, pers. comm. to RE Fordyce, 1988). The holotype specimen of the billfish *Aglyptorhynchus hakataramea* Gottfried, Fordyce & Rust, 2012 (OU 22396) was collected from the same locality at a comparable level (both 2–3 m above the active quarry floor) and yielded a sample similarly lacking *Globoquadrina dehiscens* and preserving the benthic foraminifer *Notorotalia spinosa*, indicating Duntroonian age (Gottfried, Fordyce & Rust, 2012) and further suggesting a Duntroonian age assignment for OU 22163.

OU 22075 was collected from the lower Otekaike Limestone at Springside (Figs. 1 and 2B; North Otago; New Zealand Fossil Record number I41/f0184), from a massive glauconitic horizon corresponding to transitional strata of the Kokoamu-Otekaike succession (= basalmost Maerewhenua Member of the Otekaike Limestone).

## Description

The description is primarily based on the holotype adult specimen, but combines information from all known specimens. Where anatomical features in the juveniles differ, or if structures missing in the adult are present in the juveniles, they are noted.

### Premaxilla

**Figure 3:**
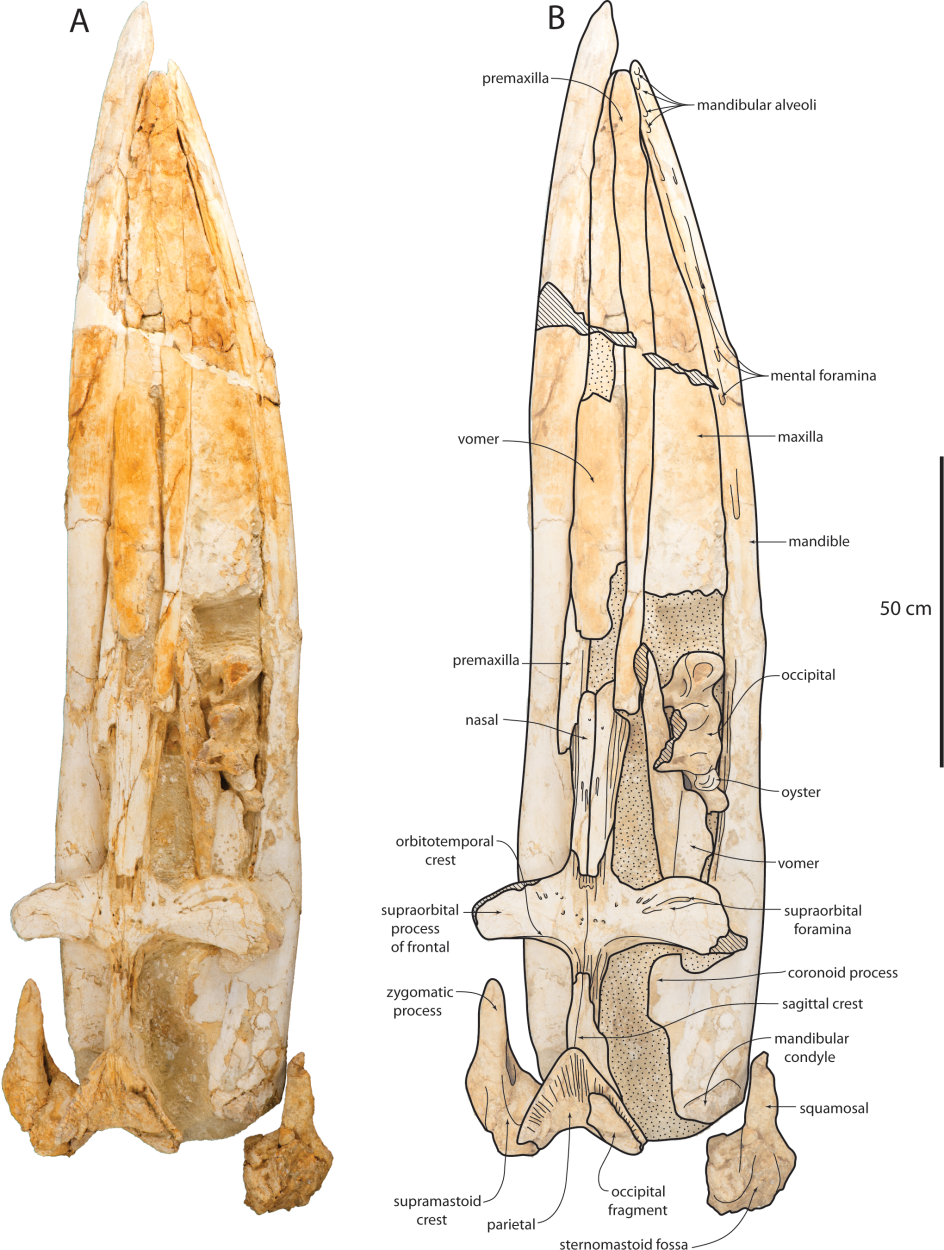
Holotype cranium (OU 22044) of *Waharoa ruwhenua* in dorsal view. (A) photograph; (B) interpretive line drawing. Stippling denotes matrix, cross-hatching denotes broken surfaces.

**Figure 4:**
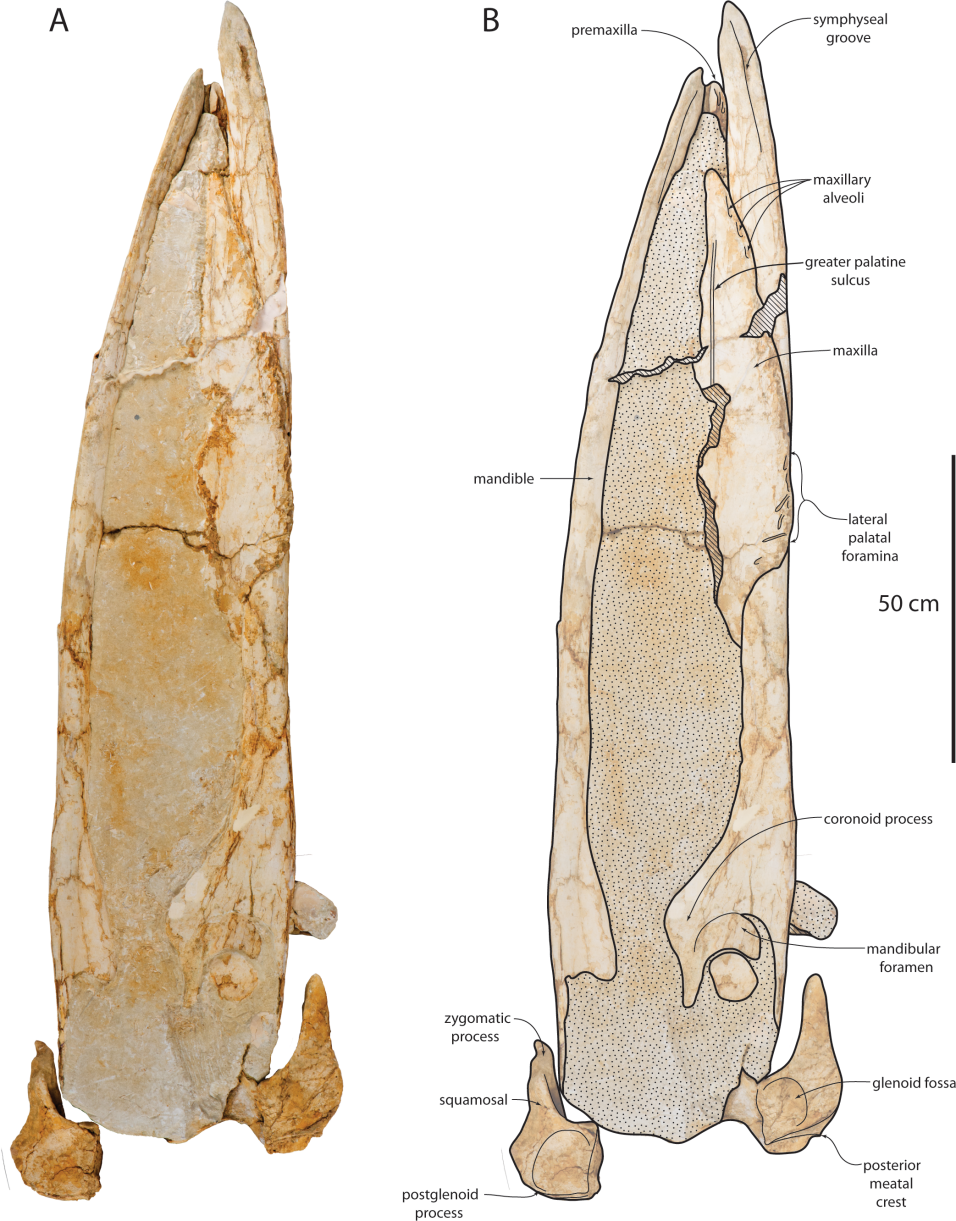
Holotype cranium (OU 22044) of *Waharoa ruwhenua* in ventral view. (A) photograph; (B) interpretive line drawing. Stippling denotes matrix, cross-hatching denotes broken surfaces.

**Figure 5:**
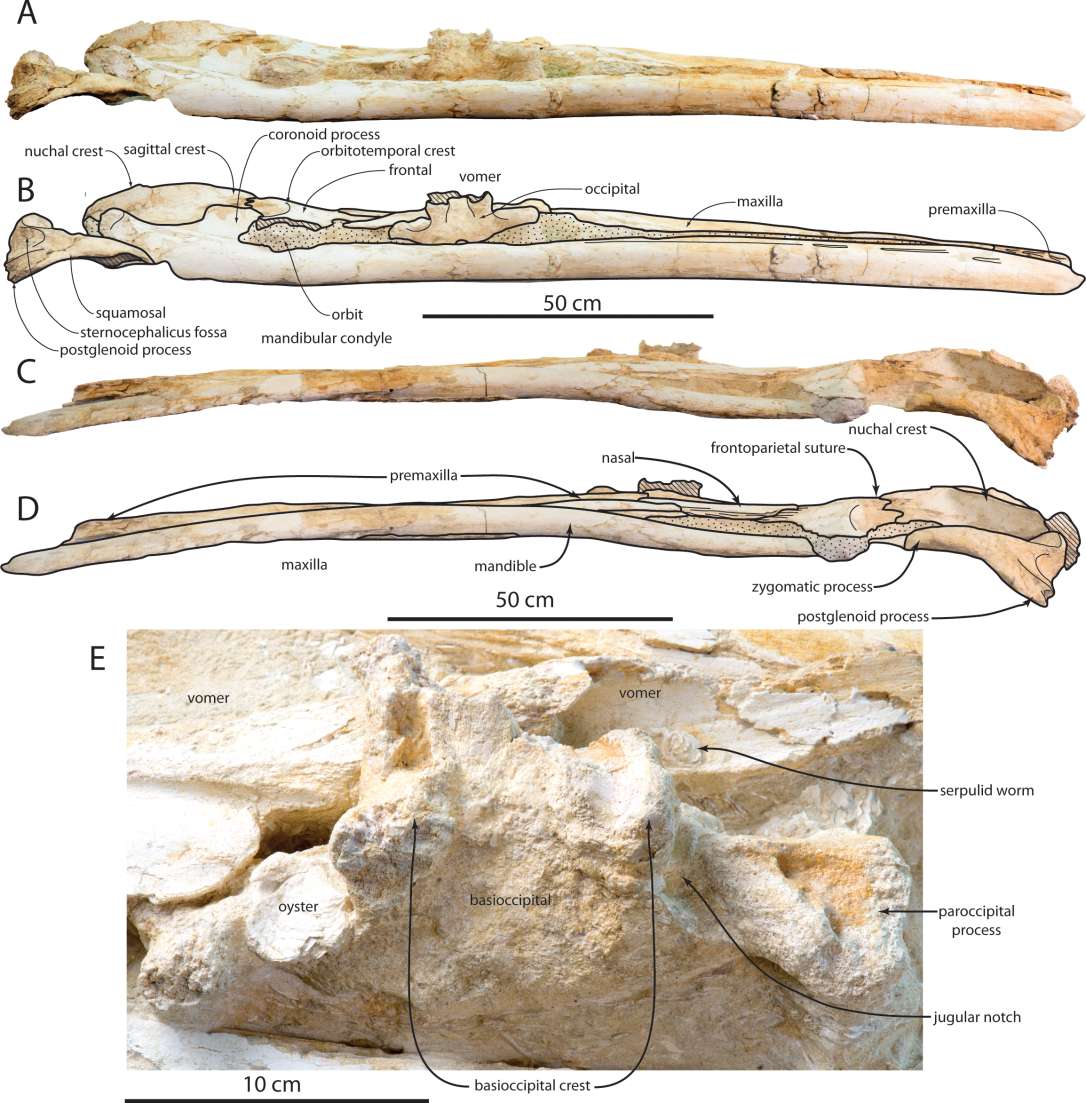
Holotype cranium (OU 22044) of *Waharoa ruwhenua*. (A) right lateral view; (B) interpretive line drawing; (C) left lateral view; (D) interpretive line drawing; (E) detail of basicranium in ventral view.

**Figure 6:**
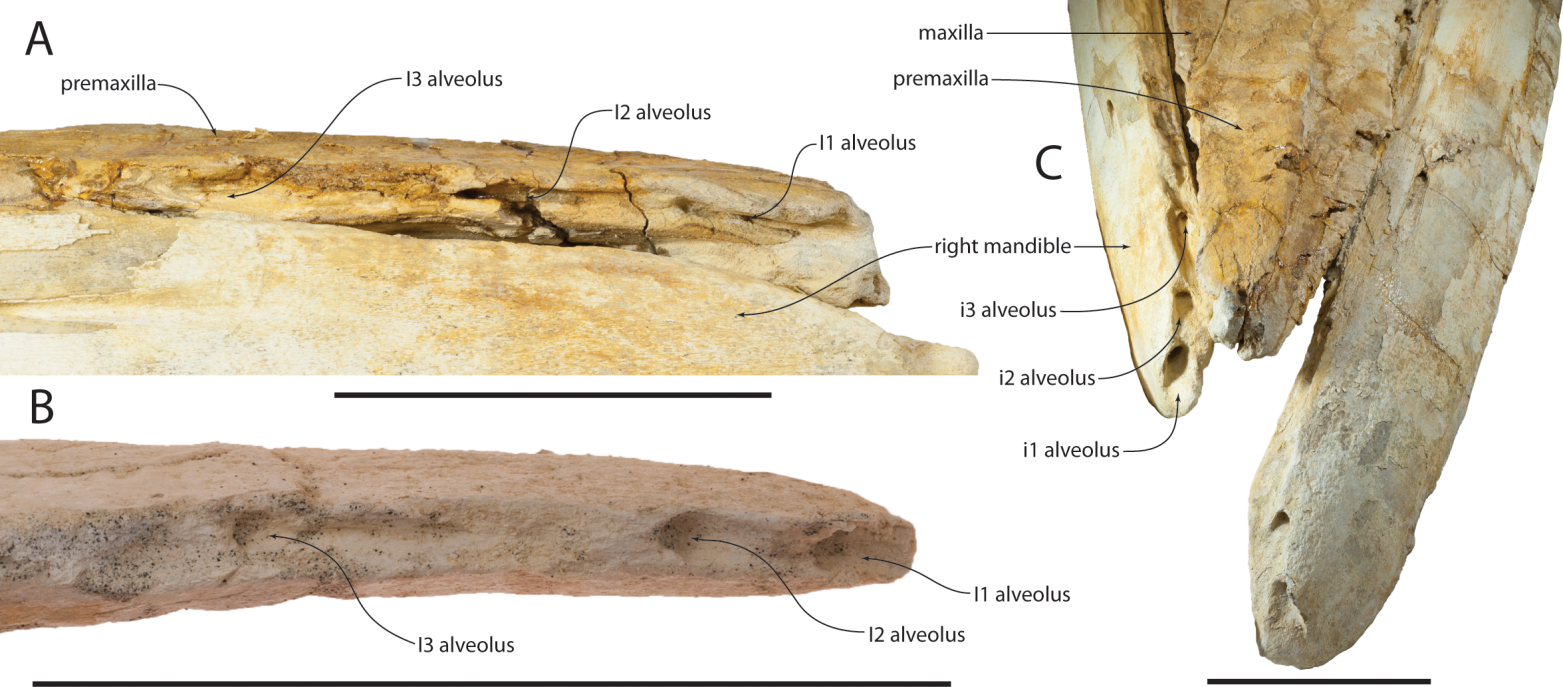
Vestigial maxillary and mandibular alveoli of *Waharoa ruwhenua*. (A) holotype (OU 22044) rostrum and mandible in right lateral view; (B) premaxilla of referred juvenile (OU 22163) in right lateral view; (C) holotype (OU 22044) rostrum and mandibles in anterodorsal view. Scale bar equals 10 cm.

**Figure 7:**
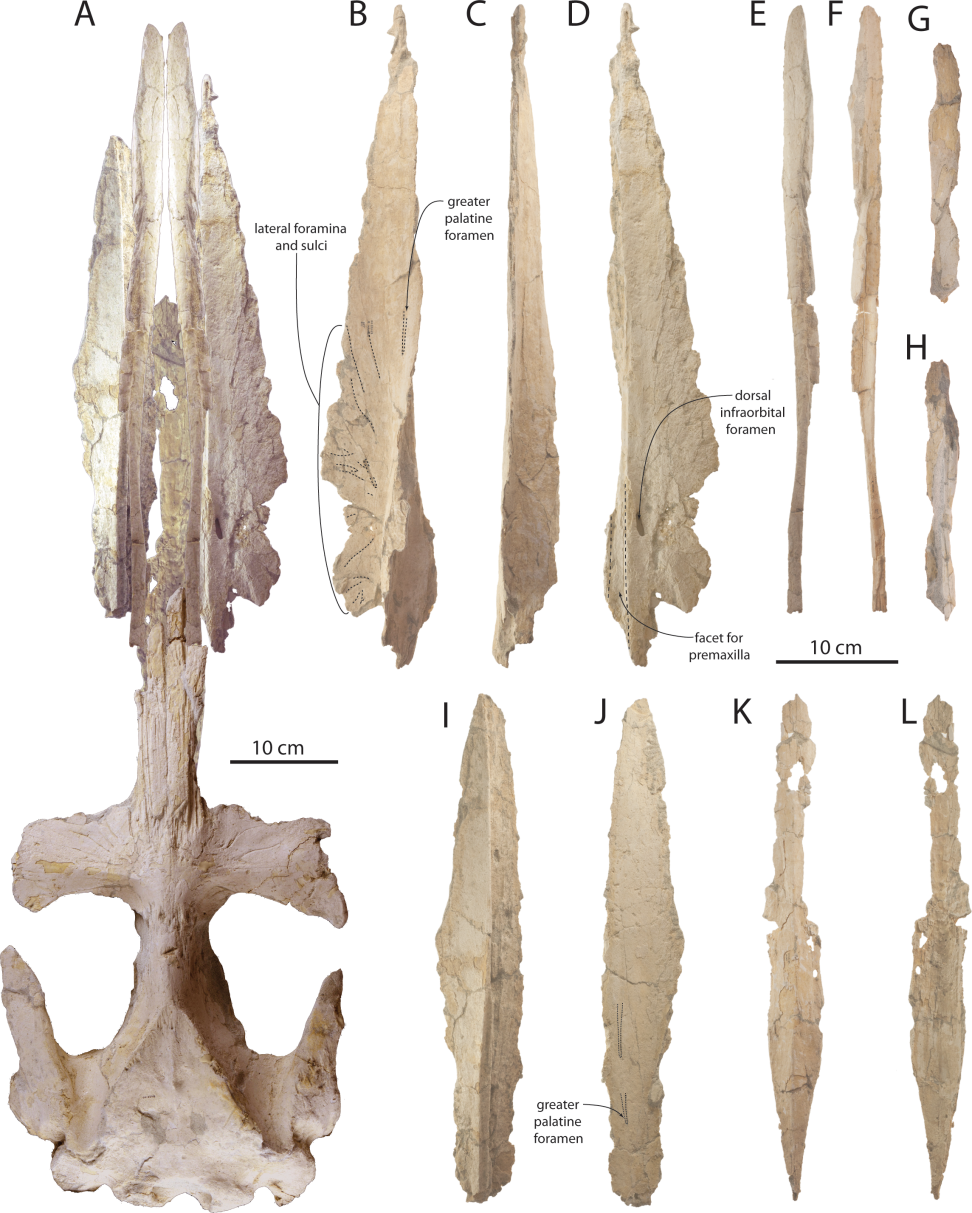
Referred large juvenile skull of *Waharoa ruwhenua* (OU 22163). (A) composite of cranium with rostral elements superimposed, with right premaxilla reflected; (B) right maxilla in ventral view; (C) right maxilla in lateral view; (D) right maxilla in dorsal view; (E) right premaxilla in dorsal view; (F) right premaxilla in ventral view; (G) left premaxilla in dorsal view; (H) left premaxilla in ventral view; (I) left maxilla in dorsal view; (J) left maxilla in ventral view; (K) vomer in ventral view; (L) vomer in dorsal view.

**Figure 8:**
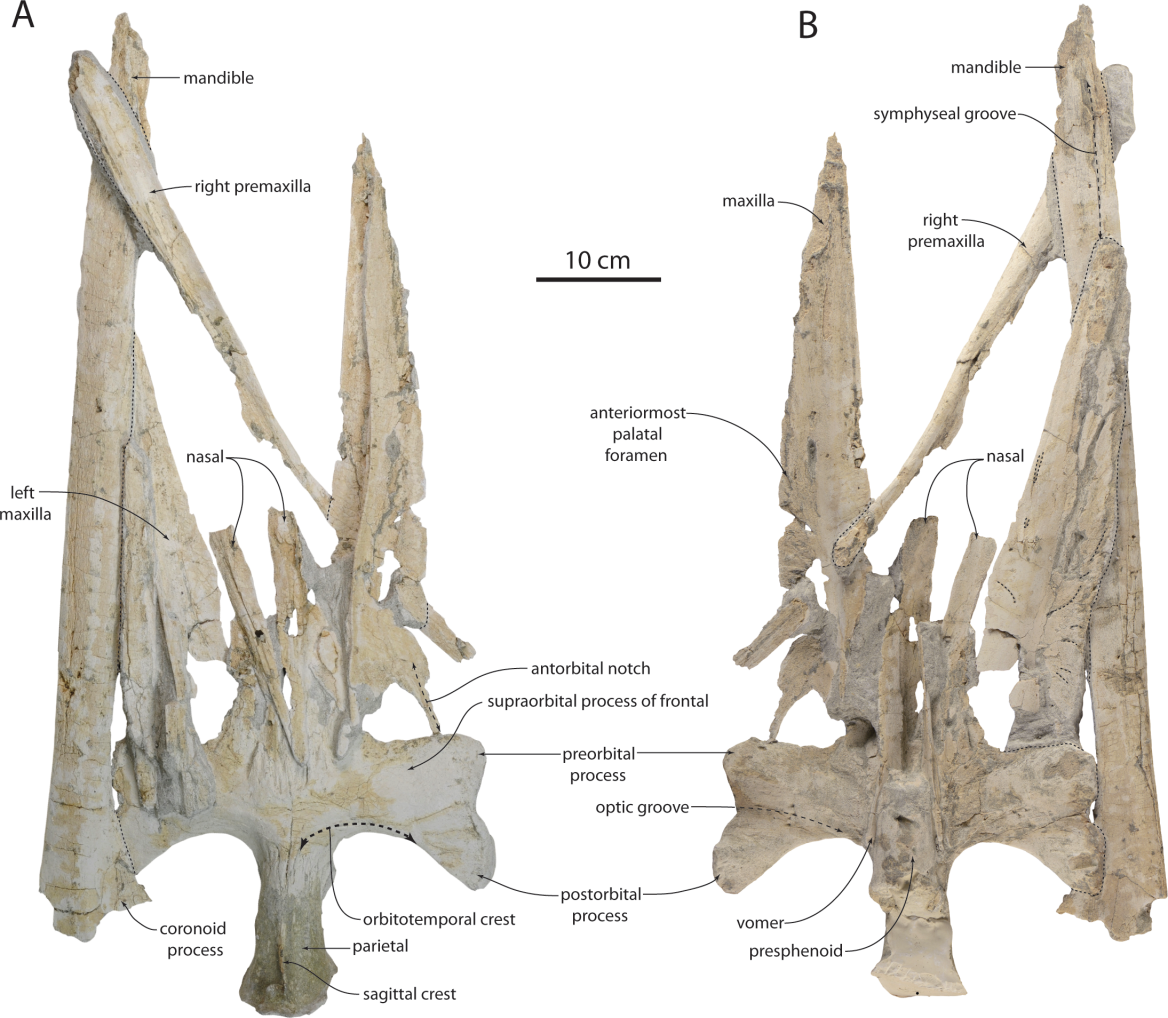
Referred small juvenile cranium and mandible of *Waharoa ruwhenua* (OU 22075). (A) dorsal view; (B) ventral view.

The posterior quarter of the premaxilla in OU 22044 (Figs. 3–6; Table 1) is missing, but complete in OU 22075 and 22163 (Figs. 7 and 8); the premaxilla is elongate, transversely slender, and anteriorly bears a flattened dorsal surface. In OU 22044 and 22163, several elongate parallel grooves are present on the lateral margin of the nasal; these grooves are contiguous with those present adjacent on the frontal forming the frontal-premaxilla suture. This indicates that the premaxilla medially overlapped and sutured to the nasal. In OU 22044 immediately anterior to the nasals, the premaxilla is transversely convex and bears a very slight longitudinally concave surface corresponding to the narial fossa. The narial fossa is transversely narrow and anteroposteriorly long, and subtle in comparison to other Eomysticetidae; the premaxillae are medially separated 56 cm anterior to the nasals, and medially contact anterior to this (but are not medially sutured together). Anterior to the narial fossa, the premaxilla gently widens transversely towards the anterior tip of the maxilla; the premaxilla forms the sharply tapered tip of the rostrum. Along the anteroventral edge of the premaxilla (anterior to the termination of the maxilla) in OU 22044 and 22163 are three anteriorly directed foramina present within a common trough (Fig. 6); these appear to be homologous with the structures reported by Okazaki (2012) as vestigial alveoli in *Yamatocetus canaliculatus*, and are identified as three incisor alveoli; preservation of these structures in OU 22075 is equivocal. The discovery of a single linguolabially flattened tooth near the maxilla of *Tokarahia* (OU 22081) raises the possibility that alveoli in *Waharoa* and *Yamatocetus* also housed adult teeth. In the disarticulated juvenile crania (OU 22075 and 22163), an elongate horizontal flange is present on the ventrolateral margin of the anterior two-thirds of the premaxilla (Figs. 7E and 7F). When the right premaxilla of OU 22163 is articulated with the maxilla, the horizontal flange is received by a medial groove on the maxilla. Posteriorly, the premaxilla is dorsoventrally flattened and splint-like; the anterior two-thirds of the premaxilla bears a longitudinal trough that deepens anteriorly.

**Table 1:** Measurements of *Waharoa ruwhenua* crania (in cm, to nearest mm).

Measurement	OU 22075	OU 22163	OU 22044
Condylobasal length	–	109.8(e)	183.3
Premaxilla length	57.0	70.9	133.1
Greatest transverse width of premaxilla	3.7	3.0	5.9
Transverse width of premaxilla at naris	1.3	1.1	2.9
Transverse width of bony naris	–	–	5(e)
Maxilla length	50.4	–	113.8
Greatest width of maxilla as preserved	7.2	8.0	12.1
Nasal length	20.5	22.4	36.1
Transverse width of anteriormost nasal	2.5	2.0	2.8
Transverse width of posteriormost nasal	1.3	1.9	3.1
Preorbital width of frontal	31.2	30.8*	–
Postorbital width of frontal	33.0	33.4	50.6*
Transverse width of ethmoid recess	10.3	10.4	–
Anteroposterior length of supraorbital process as preserved	12.4	11.0*	12.6*
Anteroposterior length of temporal fossa, frontal to subtemporal crest	–	15.7	22.3
Anteroposterior length of temporal fossa, frontal to nuchal crest	–	24.8	34.3
Length of parietal, occipital apex to frontoparietal suture	8.3	7.5	11.6
Bizygomatic width	–	32.4	27.6
Transverse width of squamosal at level of glenoid fossa	–	31.4	42.0
Exoccipital width	–	25.3	26.8
Anteroposterior thickness of paroccipital process	–	3.3	2.6*
Width of basioccipital crest	–	2.9	4.2
Width across basioccipital crests	–	9.2	11.6
Occipital shield width	–	17.2	26.0
Width across occipital condyles	–	13.5	–
Width of foramen magnum	–	5.6	–
Dorsoventral depth of foramen magnum	–	3.6	–
Anteroposterior length, exoccipital to anterior margin of supraorbital process	–	38.9	50.9(e)
Anteroposterior length, postglenoid process to anterior margin of supraorbital process	–	32.1	45.9
Anteroposterior length of occipital	–	19.9	–
Glenoid fossa, maximum diameter	–	8.0	12.7
Glenoid fossa, minimum diameter	–	5.4	9.3

**Notes.**

(e) denotes estimated measurement; asterisk (*) denotes incomplete measurement as preserved.

### Maxilla

The maxilla is narrow, triangular, and dorsally flat in lateral view (Figs. 3–5, 7 and 8; Table 1). Medially, the maxilla is tightly articulated with the premaxilla in OU 22044; posteriorly in this specimen, the premaxilla and nasal are slightly elevated with respect to the maxilla. Posteromedially, a longitudinal flat parallel sided furrow is present on the dorsal surface of the maxilla in OU 22163 (Fig. 7D) and articulates with the premaxilla. A single large dorsal infraorbital foramen is present lateral to this furrow (Fig. 7D). The lateral edge of the maxilla in all three specimens is posteriorly thin, and becomes dorsoventrally thicker anteriorly; this is most pronounced in OU 22044. The lateral edge is damaged in OU 22075 and 22163, but in OU 22044 appears to preserve a distinct alveolar groove with apparent alveoli as in the premaxilla of OU 22044 and 22163, and as in the maxilla of *Yamatocetus canaliculatus* (Okazaki, 2012). The lateral edge of the maxilla of OU 22075 and OU 22163 is damaged but appears to preserve an alveolar groove; preservation of individual alveoli is equivocal.

The ventral surface of the maxilla is flat anteriorly and posterolaterally, and in OU 22075 and 22163, the posteromedial surface of the maxilla descends ventrally to form a shallow longitudinal keel; in cross section, the keel is ventrally gently convex and smooth. In all three skulls, the anteriormost part of the palatal surface of the maxilla is barren and smooth; lateral foramina and sulci occur only on the posterior half of the exposed maxilla in OU 22044 and OU 22075 (Figs. 4 and 8) and the posterior two-thirds of the preserved maxilla in OU 22163 (Fig. 7B). In OU 22163 the anteriormost foramina are shallow, elongate, non-branching, straight, and directed anterolaterally approximately 15–20° from the sagittal plane. These open at about the anteroposterior midpoint of the preserved maxilla, and are positioned medially on the palate; similarly positioned foramina occur in *Aetiocetus weltoni* Barnes and Kimura, 1995 (Deméré & Berta, 2008), *Mammalodon colliveri* Pritchard, 1939; Fitzgerald, 2010 and *Janjucetus hunderi* Fitzgerald, 2006 (Fitzgerald, 2006: Fig. 1D); detailed dissection of *Eschrichtius robustus* Van Beneden & Gervais, 1868 has confirmed the homology of these anteromedial foramina as the greater palatine foramina (Ekdale, Deméré & Berta, 2015). Posteriorly, the lateral foramina are more variably oriented, shorter, and occasionally bifurcate. In OU 22163 and 22075, the majority of lateral sulci and foramina occur only on the horizontal portion of the maxilla, although in OU 22075 several small foramina are present posteromedially.

In OU 22075 and 22163 the maxilla bears a ventromedially descending flange, forming the lateral part of the longitudinal keel (Fig. 7B) that underlapped the vomer in life. The medial surface of the maxilla is complex and exposed only in the disarticulated rostrum of OU 22163; anteriorly, it consists of a transversely concave, medially facing smooth trough, which articulates with the anteriormost part of the premaxilla. In the middle section of each maxilla, the medial surface includes a nearly flat dorsomedially facing surface with numerous longitudinal grooves and ridges, and a deep, medially facing horizontal and longitudinal groove is present dorsally, ventrally adjacent to the dorsomedial ridge forming the medial margin of the dorsal surface of the maxilla. Manipulation of the right premaxilla and maxilla of OU 22163 indicates this horizontal groove receives the lateral flange of the premaxilla (see above). The internal choana is exposed posteriorly in OU 22163 as a ventromedially facing, anterodorsally directed and shallow trough. Ventral to the choana the articular surface for the vomer continues posteroventrally.

### Nasal

**Figure 9:**
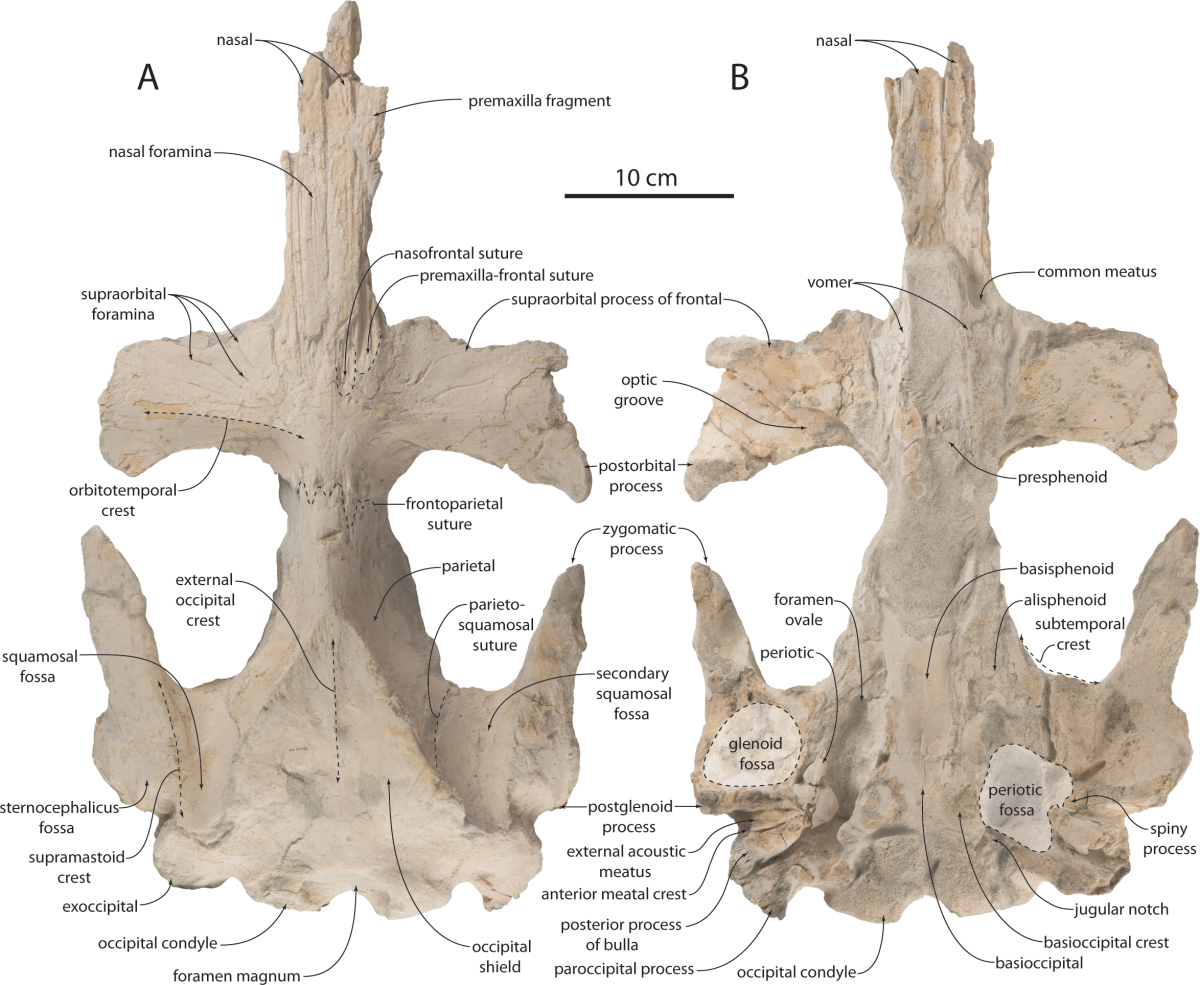
Referred large juvenile cranium of *Waharoa ruwhenua* (OU 22163). (A) dorsal view; (B) ventral view.

The nasal is elongate, rectangular, and dorsally flat (Figs. 3, 7–9 and Table 1). Several small foramina are present anteriorly. The anterior margin is unknown. The nasal anteromedially overlaps an anteriorly elongate, narrow, and triangular medial extension of the frontal. The lateral portion of the nasal bears elongate longitudinal grooves and ridges that are laterally contiguous with a similar grooved surface on the adjacent frontal for articulation with the premaxillae. It appears that the nasal process of the premaxilla overlapped and was sutured to the frontal and lateral part of the nasal, and extended nearly as far posterior as the posterior termination of the nasal with a posteriorly tapering end along the lateral side of the nasal. The ventral surface of the nasal of OU 22075 is transversely concave.

### Frontal

The supraorbital process of the frontal is horizontal and slightly (and gradually) depressed relative to the medial portion of the frontal (Figs. 3, 7–9 and Table 1). The low orbitotemporal crest is near the posterior margin. Numerous radially arranged foramina with elongate sulci are scattered on the dorsal surface of the supraorbital process. An elongate, anteromedially positioned triangular prong of the frontal extends anteriorly beneath the nasal. The orbital margin of the frontal is laterally concave in dorsal view and preserved best in OU 22075; the postorbital process is elongate and triangular. In OU 22075, the ventral part of the postorbital process is knob-like with a flattened posterior facet; the orbital margin is formed as a shallow but triangular notch, rather than evenly concave. The ventral face of the anteromedial part of the frontal is flat and set off from the optic canal by the anterolaterally curving preorbital crest. The optic canal widens laterally, and curves so that it is oriented anterolaterally proximally, but distally is oriented laterally; numerous laterally opening diploic foramina are present on the optic canal roof. The postorbital crest is low and is laterally contiguous with the postorbital process. The optic foramen is preserved as a fissure. Ventromedially, the frontal contacts the vomer along a parasagittally oriented planar suture (Fig. 9B). On the ventral surface of the anteromedial prong of the frontal, a poorly preserved common meatus (*sensu* Godfrey, Geisler & Fitzgerald, 2013) opens anteroventrally (Fig. 9B); a second potential meatus is present posterolateral to it. The posterior margin of the supraorbital process is posteriorly concave in dorsal view, and posterior to the supraorbital process the frontal forms the anteromedial margin of the temporal fossa, near the frontoparietal suture.

### Vomer

The vomer is elongate, thin-walled, and lanceolate with damaged lateral and anterior margins in all three specimens; it forms a shallow mesorostral groove but a deep palatal keel is not developed (Figs. 3, 4 and 7K–7L). The vomer of OU 22163 (Figs. 7K–7L) is posteriorly triangular and attenuate in ventral outline with a well-developed horizontal ridge with a dorsally adjacent longitudinal furrow for the articulation of the pterygoid. In OU 22044, the vomer exhibits dorsally ascending, parasagittally oriented sheet-like vomerine wings. Disarticulation in all specimens precludes assessment of posterior articulation with other basicranial elements or the degree of ventral exposure between the maxillae.

### Parietal

The parietal forms most of the shallowly concave medial wall of the temporal fossa, and anteriorly meets the frontals in the interorbital region along an irregular, anastomosing, roughly transverse-anterodorsally trending suture positioned within the anterior quarter of the temporal fossa (Figs. 3, 8, 9 and Table 1). The nuchal crest slightly overhangs the parietal in dorsal view. The parietal forms an elongate and transversely narrow interorbital region; the parietals are fused medially but do not form a sharp sagittal crest in the interorbital region. Posteriorly the parietal joins the squamosal in a sinuous suture that descends anterolaterally (in dorsal aspect). The parietal appears to contact remnants of the alisphenoid at the ventral extremity of the temporal fossa; a large portion of the medial wall of the temporal fossa is missing bone on either side posterior to the frontoparietal suture, confusing details of the parietal-alisphenoid contact. A separate interparietal is not evident in OU 22044 or 22163.

### Sphenoid

**Figure 10:**
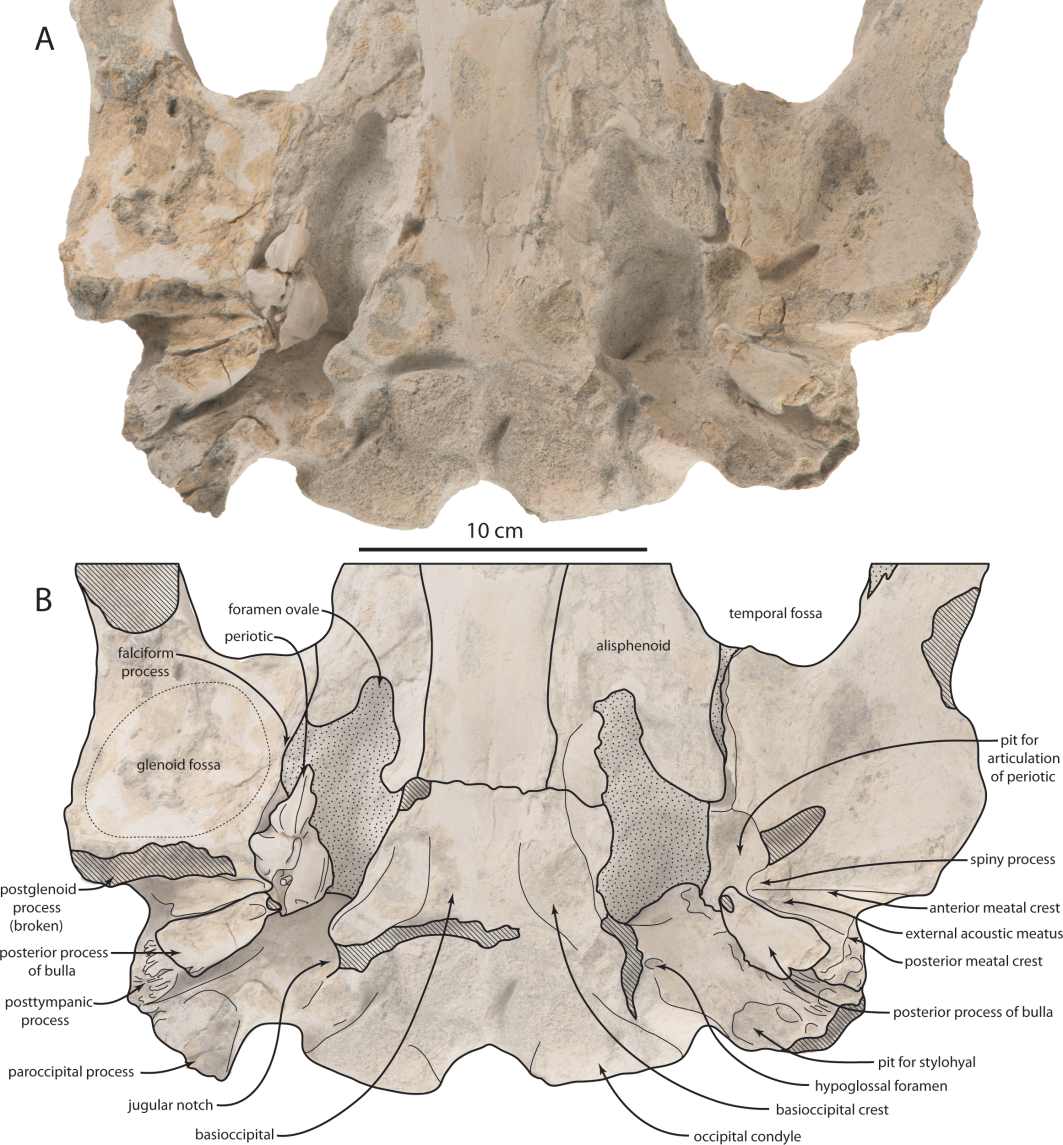
Basicranium of large juvenile of *Waharoa ruwhenua* (OU 22163) in ventral view. (A) photograph; (B) interpretive line drawing.

Details of the sphenoid are evident only in OU 22163 (Fig. 10). The basisphenoid is slightly narrower than the basioccipital crests, and is ventrally rectangular; a low, linear and parasagittally oriented crest extends anteriorly from the basioccipital crest. The basioccipital-basisphenoid suture is open and unfused. It is unclear whether any pterygoid remains owing to poor preservation. The alisphenoid ascends from this crest and forms a triangular horizontal sheet that articulates laterally with the anteroventral portion of the squamosal; contacts with other bones in the ventral part of the temporal fossa are equivocal owing to damage. The foramen ovale is preserved as a circular notch in the posterior margin of the alisphenoid.

### Occipital

The occipital is incompletely preserved in OU 22044, and fragments of the lateral margins of the supraoccipital remain, and the exoccipitals and basioccipital remain in articulation, but disarticulated from the braincase (Fig. 3 and Table 1). The occipital of OU 22163 is well-preserved and complete (Figs. 7, 9 and Table 1). The occipital shield of OU 22044 would have been slightly transversely concave and triangular in shape (based on the parietal); the occipital shield of OU 22163 appears somewhat more transversely flattened. A low external occipital crest is present along the anterior portion of the shield in OU 220163; a pair of occipital fontanelles is present in OU 22163, the left fontanelle being somewhat taphonomically enlarged. The apex of the shield appears somewhat more acute in OU 22044 than in 22163. In OU 22044 and 22163, the lateral edge of the occipital shield is convex in dorsal view, making the widest part of the shield just anterior to the posterior termination of the nuchal crest. The nuchal crest overhangs the temporal fossa to a greater degree in OU 22044 than in OU 22163.

The basioccipital is relatively wide in comparison to the width of the exoccipital in OU 22044; the basioccipital is a roughly rectangular plate with large, inflated and anteromedially oriented basioccipital crests (Fig. 5C). Lateral to the basioccipital crest, an ascending longitudinal flange forms the medial margin of the periotic fossa. The paroccipital process is anteroposteriorly thickened (Figs. 5C and 9B), and a large anteroventral fossa is present in OU 22163 for articulation with the stylohyal (Uhen, 2004); in this specimen, there is additionally a posteroventrally directed accessory spine on the paroccipital process, ventrally adjacent to the fossa. The anterior surface of the paroccipital process is concave where it receives the posterior processes of the periotic and tympanic bulla. In OU 22163 the left hypoglossal canal opens approximately 15 mm from the ventral margin of the exoccipital, and the right opens 30 mm from the ventral margin; both canals open ventrolaterally. The paroccipital process extends posterolaterally in ventral view (Figs. 5C and 9B), unlike *Tohoraata raekohao* and *Tohoraata waitakiensis* (Boessenecker & Fordyce, 2015b). Dorsal condyloid fossae are not developed in OU 22163 (Fig. 9A). The foramen magnum is oval and dorsoventrally shallow, as are the condyles. A condyloid pedicle is not developed, and the condyles bear a punctate texture.

### Squamosal

Both squamosals of the holotype are disarticulated from the braincase (Fig. 3 and Table 1); the left squamosal may be more confidently oriented with respect to the braincase. The zygomatic process is elongate, and lacks a supramastoid crest; it is circular in cross-section and evenly convex dorsally. The supramastoid crest is restricted to the posterior of the squamosal as a small anterior extension of the nuchal crest, but terminates far posterior to the temporal fossa. In lateral view, the zygomatic process is dorsally convex and ventrally concave; the anterior half of the zygomatic process curves anteroventrally. Deeply incised grooves are present on the medial surface of both squamosals, interpreted here to be taphonomic as only damaged bone surfaces are evident within, although it is possible that bioerosion preferentially degrades this part of the squamosal owing to a histological difference. In OU 22163, the zygomatic process is medially bowed in dorsal view (Fig. 9A), a feature shared with *Tokarahia kauaeroa* and *Tokarahia lophocephalus* (Boessenecker & Fordyce, in press).

The ventral surface of the zygomatic process is flat, and widens posteriorly toward the shallowly concave oval glenoid fossa (Figs. 4, 9B and 10); the glenoid fossa is clearly encircled by a low ridge. The long axis of the glenoid fossa is oriented anteromedially. A tympanosquamosal recess is absent, and the glenoid fossa abuts the falciform process of the squamosal. Dorsal to the postglenoid process and anterior meatal crest, the postglenoid notch comprises the lateral portion of the external acoustic meatus, which forms an elongate, laterally widening trough; it is defined posteriorly by a low and laterally diminishing posterior meatal crest. Dorsal to the posterior meatal crest, a large posterolaterally facing sternocephalicus fossa is present; the sternocephalicus fossa occupies 75% of the dorsoventral height of the posterior squamosal. Medial to the sternocephalicus fossa, a deep pit receives the lateral extremity of the anterolaterally curving paroccipital process. The shallow secondary squamosal fossa (Sanders & Barnes, 2002) forms an anteroposteriorly directed trough between the dorsal margin of the zygomatic process and the nuchal crest.

### Periotic

**Figure 11:**
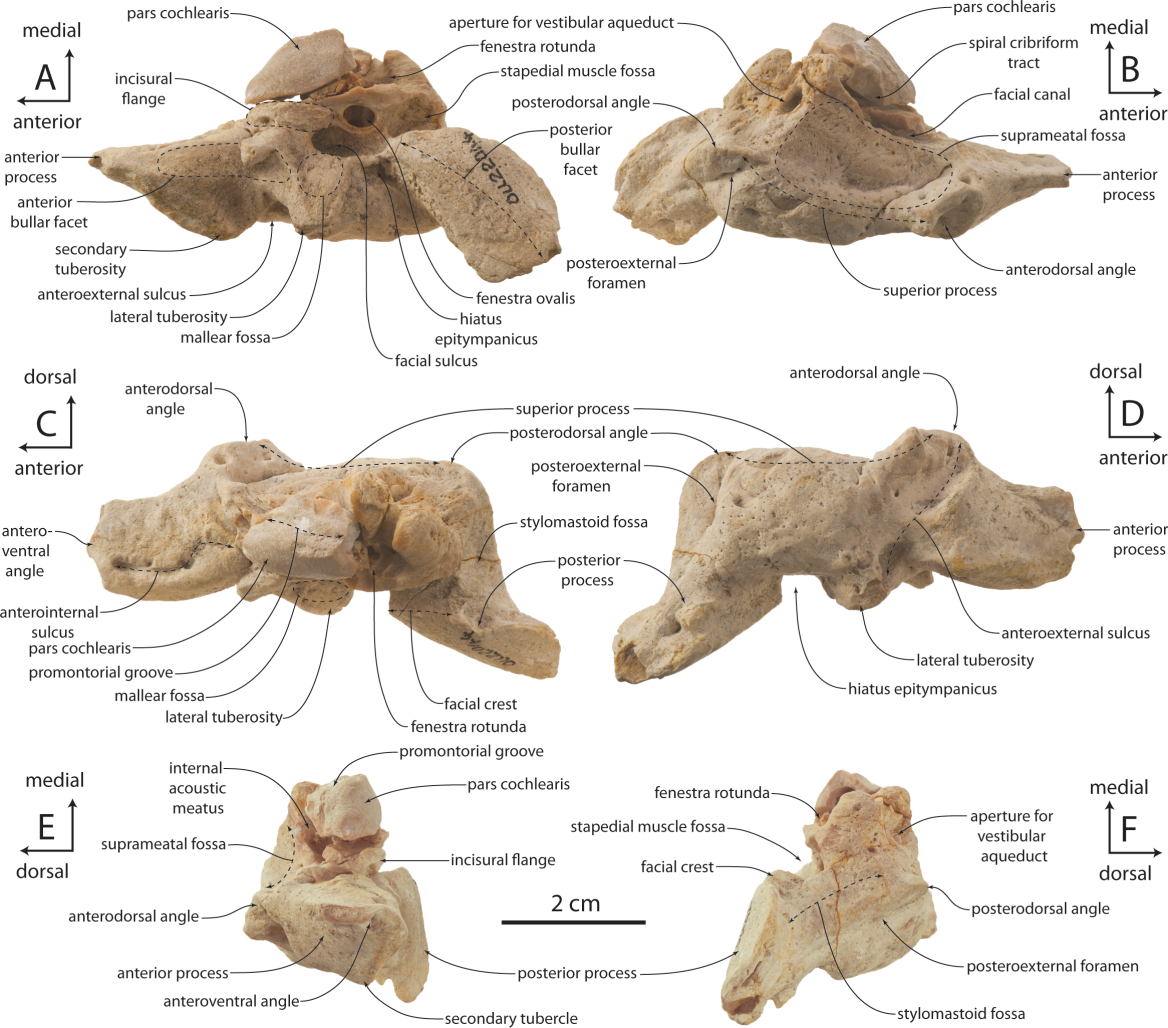
Right periotic of *Waharoa ruwhenua* holotype (OU 22044). (A) ventral view; (B) dorsal view; (C) medial view; (D) lateral view; (E) anterior view; (F) posterior view.

**Figure 12:**
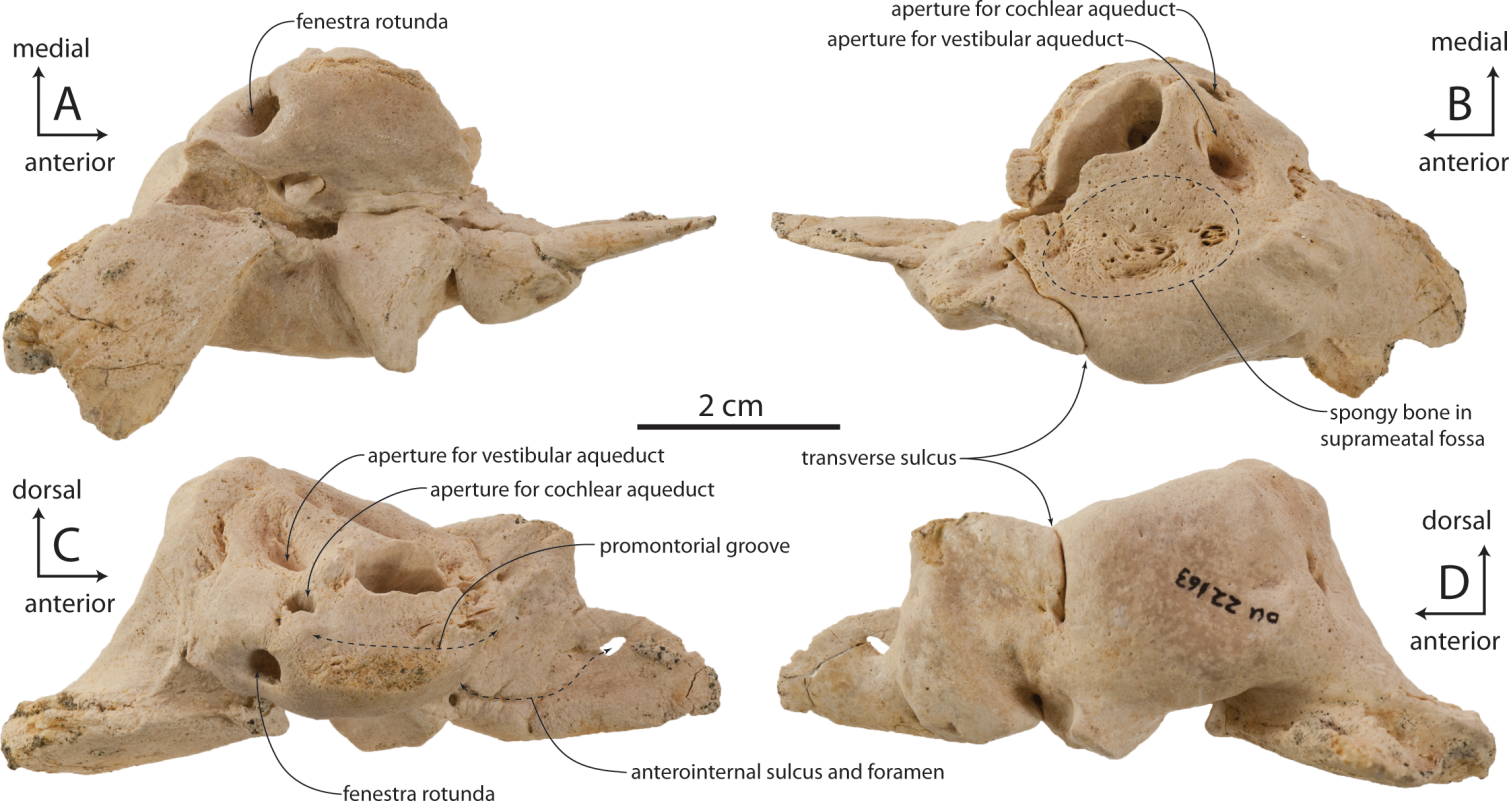
Left periotic of large juvenile of *Waharoa ruwhenua* (OU 22163). (A) ventral view; (B) dorsal view; (C) medial view; (D) lateral view.

**Figure 13:**
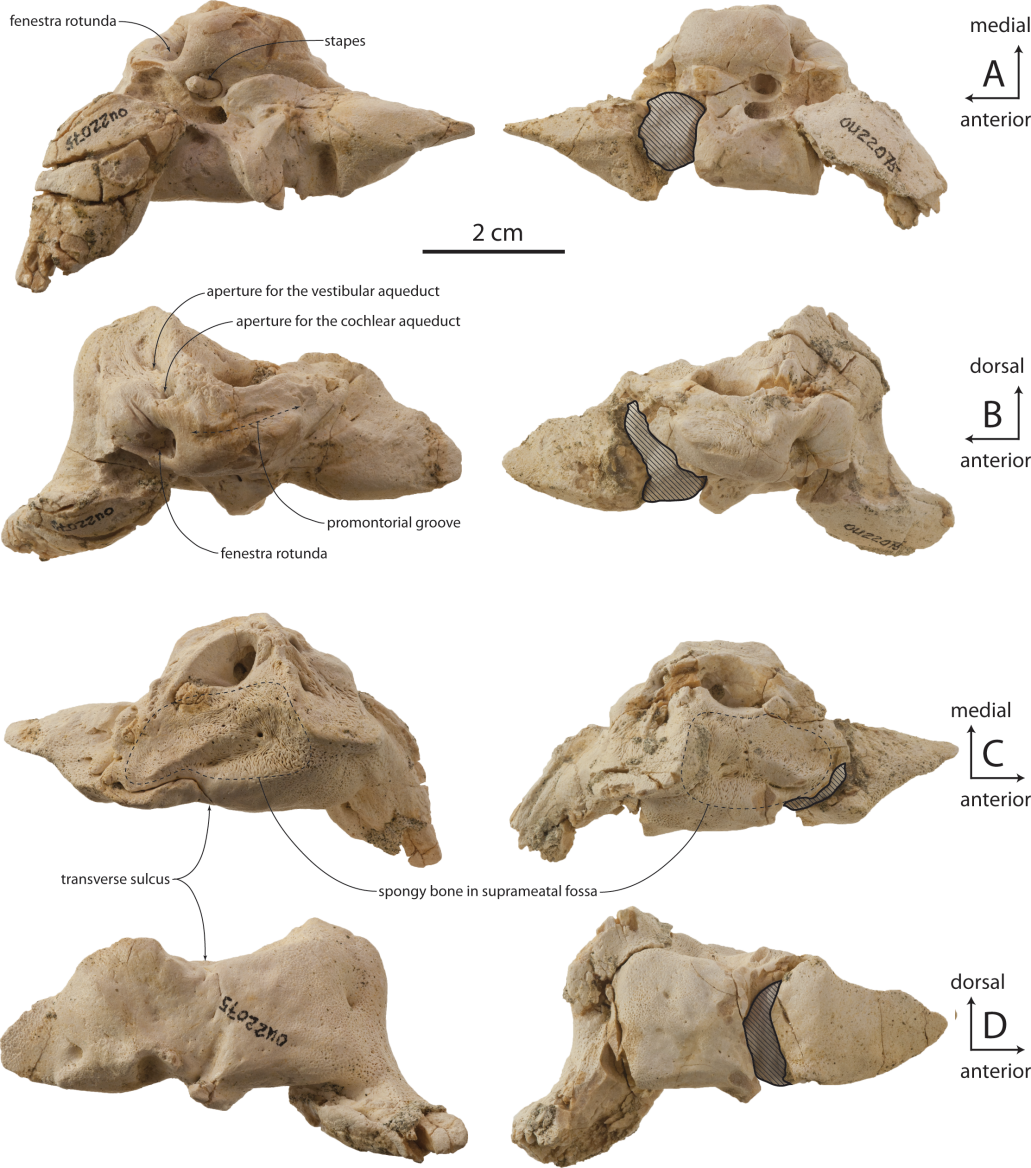
Periotics of small juvenile of *Waharoa ruwhenua* (OU 22075). (A) ventral view; (B) dorsal view; (C) medial view; (D) lateral view.

The holotype right periotic is well-preserved but has a damaged pars cochlearis (Fig. 11 and Table 2). The periotic is relatively small and exhibits a gracile body and short anterior and posterior processes (82% and 100% of pars cochlearis length, respectively). The anterior process is transversely swollen posteriorly where a prominent, laterally situated secondary tuberosity is present anterior to the lateral tuberosity; the secondary tuberosity is separated from the lateral tuberosity by deep pit on the lateral surface, which anteroventrally excavates the lateral tuberosity. A second, shallow ventrally facing fossa is present on the ventrolateral surface of the anterior process; the ventromedial surface forms a transversely narrow, anteriorly tapering, and longitudinally convex anterior bullar facet for articulation of the anterior process. Anteriorly the anterior process tapers transversely into a triangular, bladelike process; in OU 22044 and 22163, the medial surface bears a narrow and finely incised sulcus which terminates anteriorly in a small foramen that completely pierces the anterior process (Figs. 12C and 12D); such a foramen is absent in OU 22075 (Fig. 13B). The anterodorsal angle is positioned relatively far posteriorly, at the level of the anterior margin of the pars cochlearis. The margin between the anterodorsal and anteroventral angles is concave. In OU 22075 and 22163 (Figs. 12 and 13), a broad trough is instead present; these troughs, and the fossa in OU 22044, are interpreted as the anterolateral sulcus.

**Table 2:** Measurements of periotics of *Waharoa ruwhenua*, in mm, to nearest 0.01 mm.

Measurement	OU 22075	OU 22163	OU 22044
Greatest anteroposterior length	69.37	71.74	66.52
Anteroposterior length of pars cochlearis	32.28	28.87	30.52
Dorsoventral depth of pars cochlearis	15.52	12.52	–
Transverse width of pars cochlearis	10.62	12.72	–
Anteroposterior length, anterior margin fenestra ovalis to anterior pars cochlearis	17.44	14.75	13.57
Anteroposterior length, anterior margin fenestra rotunda to anterior pars cochlearis	23.79	21.88	18(e)
Anteroposterior length of anterior process	20.20	24.39	18.66*
Transverse width of anterior process	15.45	10.67	14.67
Dorsoventral depth of anterior process	21.49	22.29	20.02
Anteroposterior length, anterior process to anterodorsal angle	29.21	24.52	22.76*
Anteroposterior length, anterior process to posterodorsal angle	29.34	24.36	23.09*
Greatest anteroposterior length of internal acoustic meatus	15.15	18.23	18.57
Transverse width of internal acoustic meatus	7.39	6.54	–
Depth of crest between foramen singulare and spiral cribriform tract from meatal rim	6.50	6.54	–
Depth of crista transversa from meatal rim	–	7.66	–
Length of stapedial muscle fossa	8.59	9.25	8.19
Greatest length of posterior bullar facet	33.66	30.83*	27.49*
Maximum transverse width of posterior bullar facet	17.42	18.07	15.51

**Notes.**

(e) denotes estimated measurement; asterisk (*) denotes incomplete measurement as preserved.

The lateral tuberosity is triangular, laterally directed, and lies lateral to a shallow mallear fossa (Figs. 11A, 12A and 13A). The facial canal opens ventrally just medial to the mallear fossa; in OU 22075, the canal is smaller than and does not open further anterior than the fenestra ovalis; in OU 22163, the canal is of similar size but opens slightly further anterior than the fenestra ovalis. Lastly, in OU 22044, the ventral opening of the facial canal is much larger than the fenestra ovalis (151% of transverse width of fenestra ovalis), and opens much further anterior to the fenestra ovalis. In all three specimens, an oval incisural flange (*sensu* Boessenecker & Fordyce, 2015b) is closely appressed and laps medially onto the base of the pars cochlearis anterior to the fenestra ovalis; it is demarcated laterally by a fine sulcus, which is more deeply incised in OU 22075.

**Figure 14:**
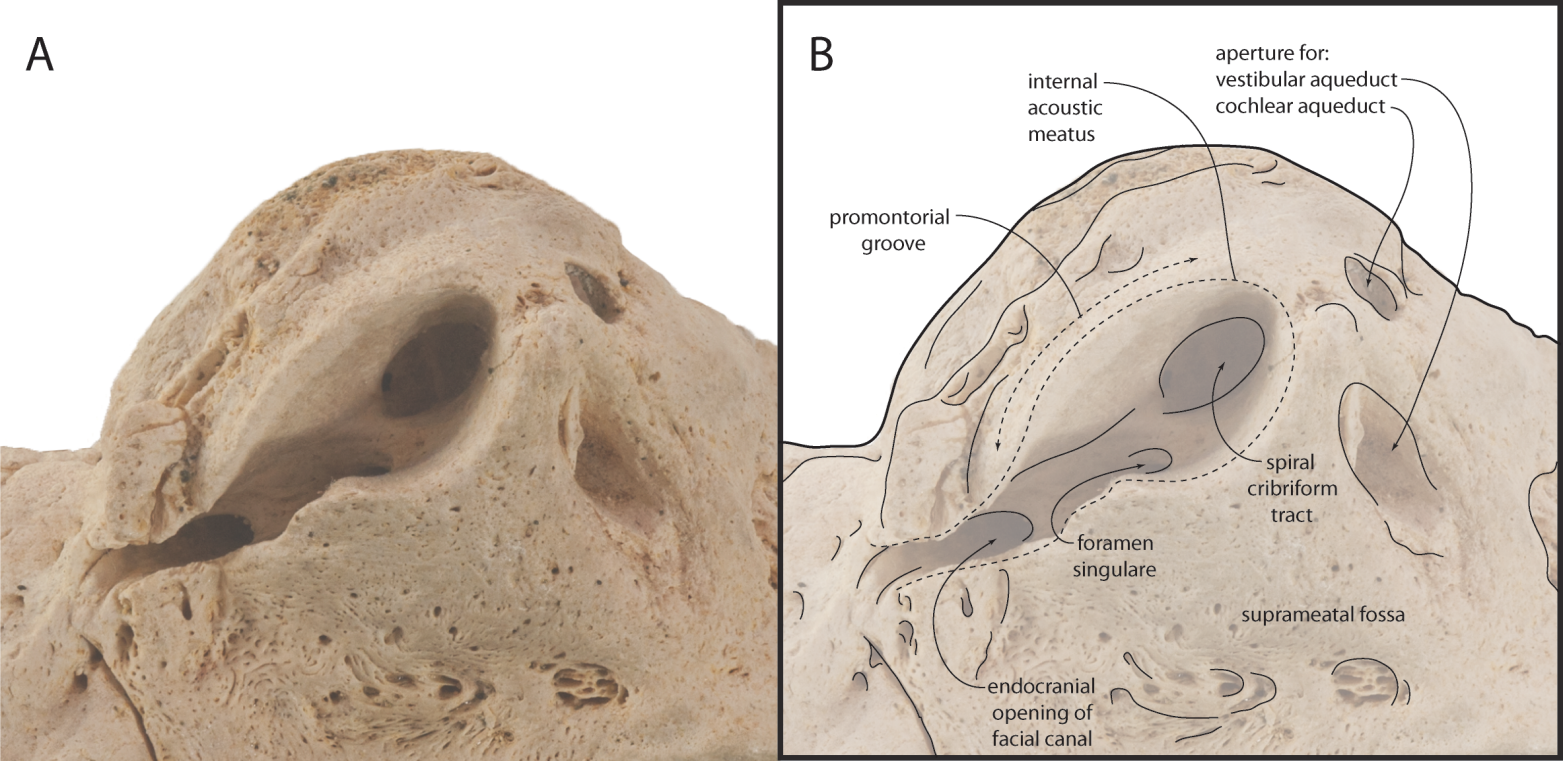
Detail of dorsal face of large juvenile periotic of *Waharoa ruwhenua* (OU 22163) in dorsal view. (A) photograph; (B) interpretive line drawing.

The pars cochlearis is anteroposteriorly elongate, hemispherical, and dorsoventrally shallow (Figs. 11–13); the internal acoustic meatus is anterolaterally directed and teardrop-shaped. The endocranial opening of the facial canal is anterior to the spiral cribriform tract and foramen singulare, which are separated by a prominent crest within the meatus (Fig. 14). The opening for the cochlear aqueduct is small and circular, while that of the vestibular aqueduct is larger and oval-shaped; both are set into a slight fossa and positioned posterior to the internal acoustic meatus. Lateral to the meatus lies the shallow and relatively small suprameatal fossa, which is bounded laterally by the discontinuous and low superior process. The superior process is reduced to low ridge with two apices (the anterodorsal angle and posterodorsal angle).The caudal tympanic process is a swollen tubercle. The shallow stylomastoid fossa is bordered ventrally by the facial crest of the posterior process. The stapedial muscle fossa is separated from the stylomastoid fossa by a sharp crest; it is deeply concave and finely sculptured, and not clearly separated from the facial sulcus.

### Accessory ossicle

**Figure 15:**
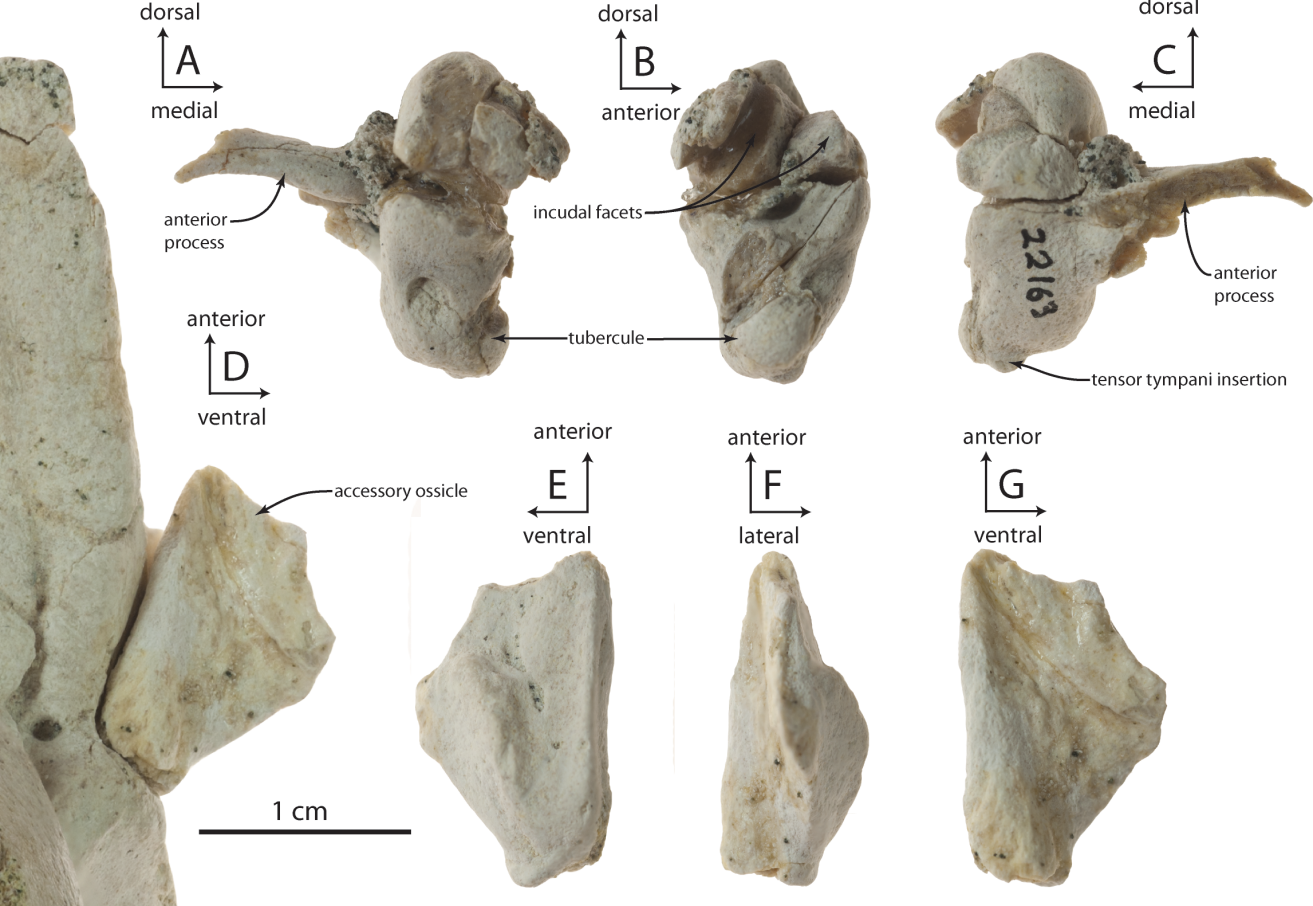
Left accessory ossicle and left malleus of large juvenile of *Waharoa ruwhenua* (OU 22163). (A) malleus in posterior view; (B) malleus in medial view; (C) malleus in anterior view; (D) accessory ossicle in articulation with periotic in medial view; (E) accessory ossicle in lateral view; (F) accessory ossicle in ventral view; (G) accessory ossicle in medial view.

OU 22163 preserves an isolated left accessory ossicle (Figs. 15D–15G). It is small (13 mm long anteroposteriorly), subtriangular in medial or lateral view, and transversely compressed. Dorsally the ossicle bears a flattened, lanceolate facet for articulation with the anterior bullar facet of the periotic, while the ventral edge of the ossicle is flattened and bladelike. Medially a longitudinal trough is developed, and laterally a slight longitudinal ridge is present. Articular relationships between the accessory ossicle and outer lip of the tympanic bulla are uncertain, but the ossicle tightly articulates with the posterior half of the anterior bullar facet of the periotic (Fig. 15D).

### Malleus

OU 22163 includes fragmentary left and right mallei (Figs. 15A–15C), while the right tympanic bulla of OU 22075 and the isolated left tympanic bulla OU 22140 both preserve mallei in articulation; no malleus is preserved with the holotype. The malleus is separated from the sigmoid process of the bulla by a minute gap. Anteriorly, the sulcus for the chorda tympani is continuous from the outer lip onto the anterior process of the malleus, and culminates medially in a blind fossa on the anteromedial surface of the malleus. In medial view, the malleus is suboval in shape with its long axis directed vertically. The anteroposteriorly narrower but globular tubercule extends anteroventrally, and bears a minute rugosity at its ventral apex for the tensor tympani insertion. Between the tubercule and the articular surfaces for the incus, an oblique anteroventrally descending ridge is developed on the medial surface of the malleus, emanating from below the posterior incudal articular surface. The malleus differs little from that of archaeocetes such as *Dorudon atrox* (Uhen, 2004: Fig. 47A–47C), except in possessing a bulbous and short rather than conical and long tubercule. Relative to extant mysticetes (*Balaenoptera*, *Caperea*), the malleus is absolutely smaller and has a proportionally smaller anterior process with a narrower sulcus for the chorda tympani, but shares a globose tubercule.

### Tympanic bulla

**Figure 16:**
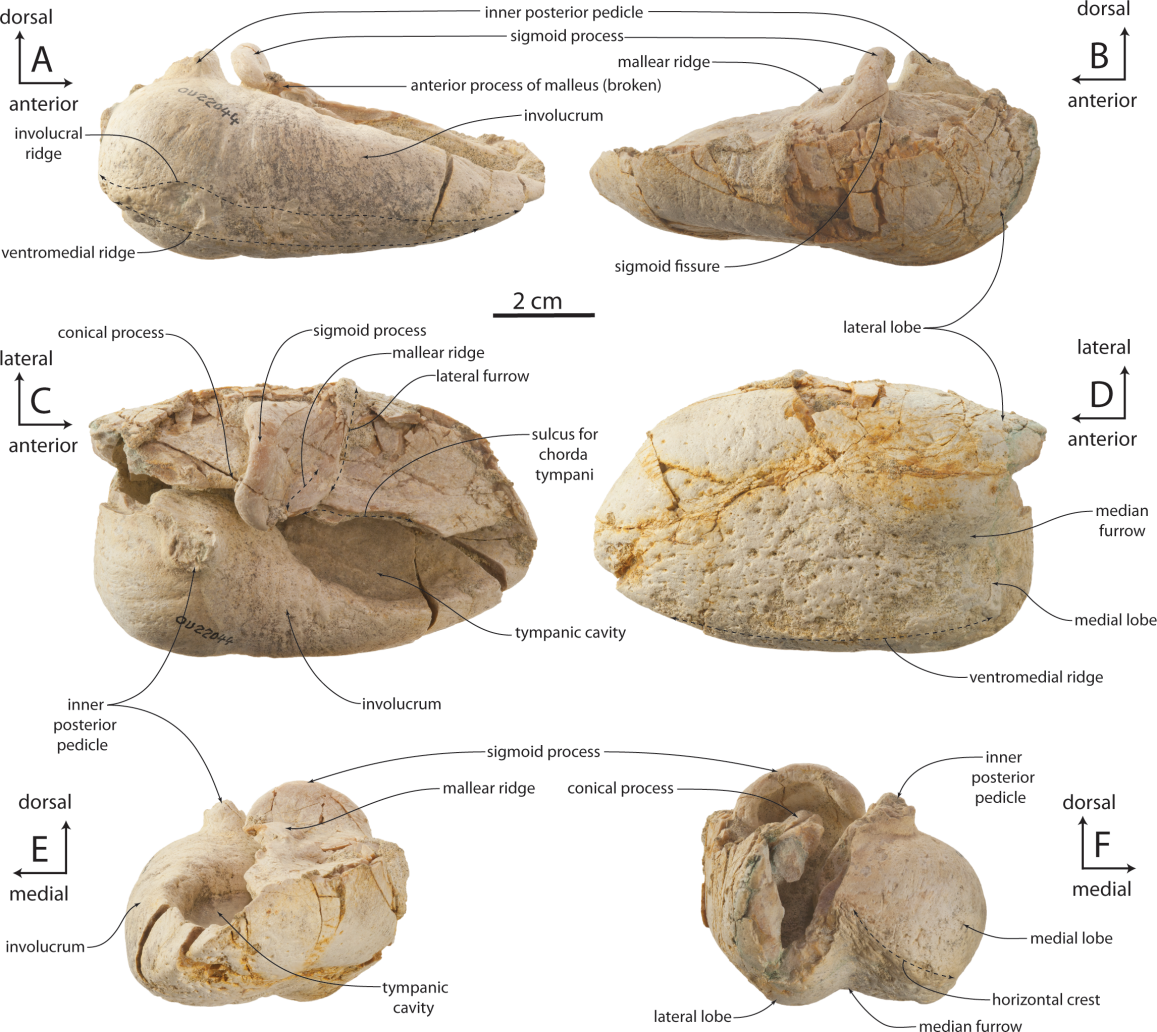
Right tympanic bulla of *Waharoa ruwhenua* holotype (OU 22044). (A) medial view; (B) lateral view; (C) dorsal view; (D) ventral view; (E) anterior view; (F) posterior view.

**Figure 17:**
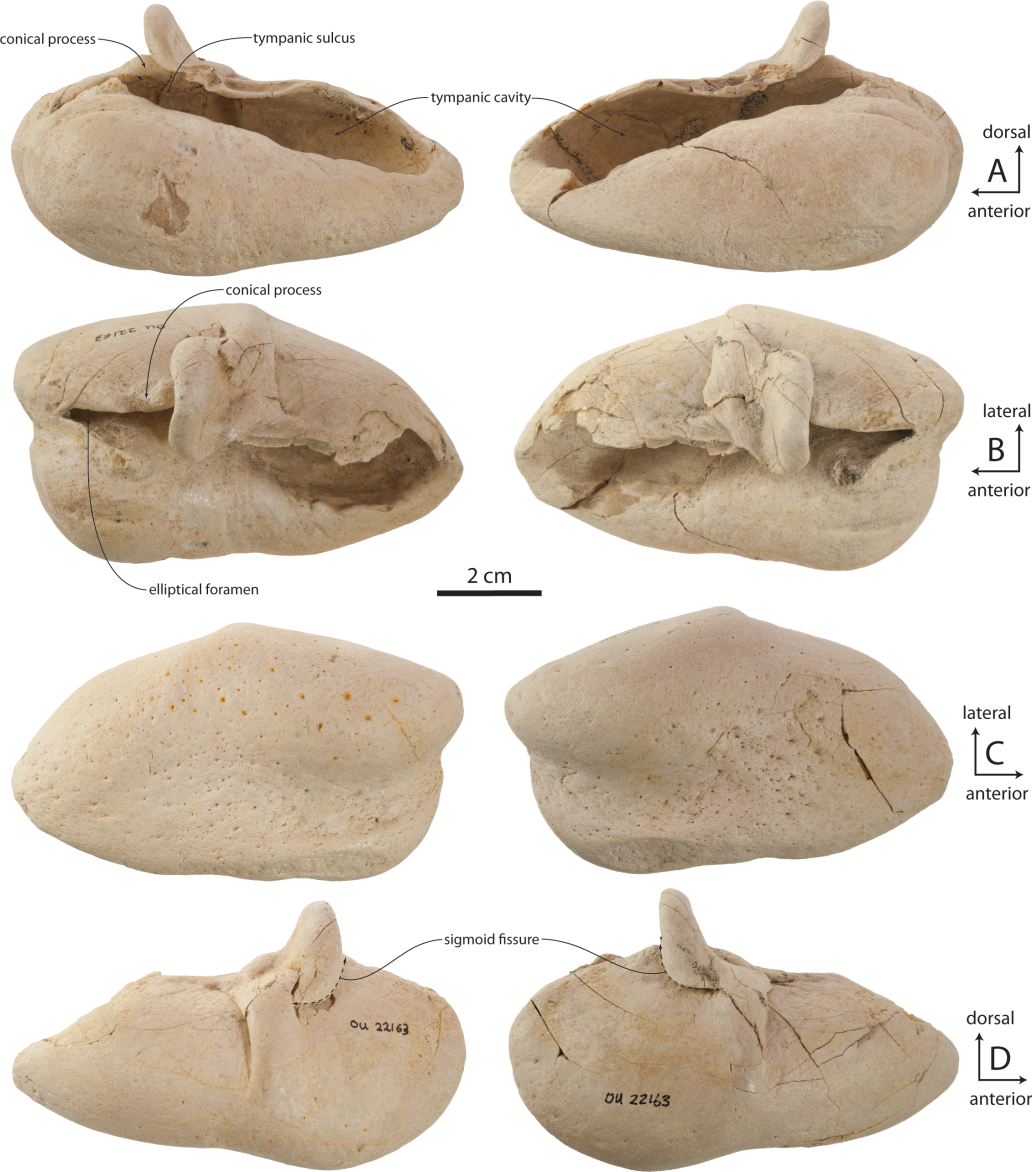
Tympanic bullae of large juvenile of *Waharoa ruwhenua* (OU 22163). (A) medial view; (B) dorsal view; (C) ventral view; (D) lateral view.

**Figure 18:**
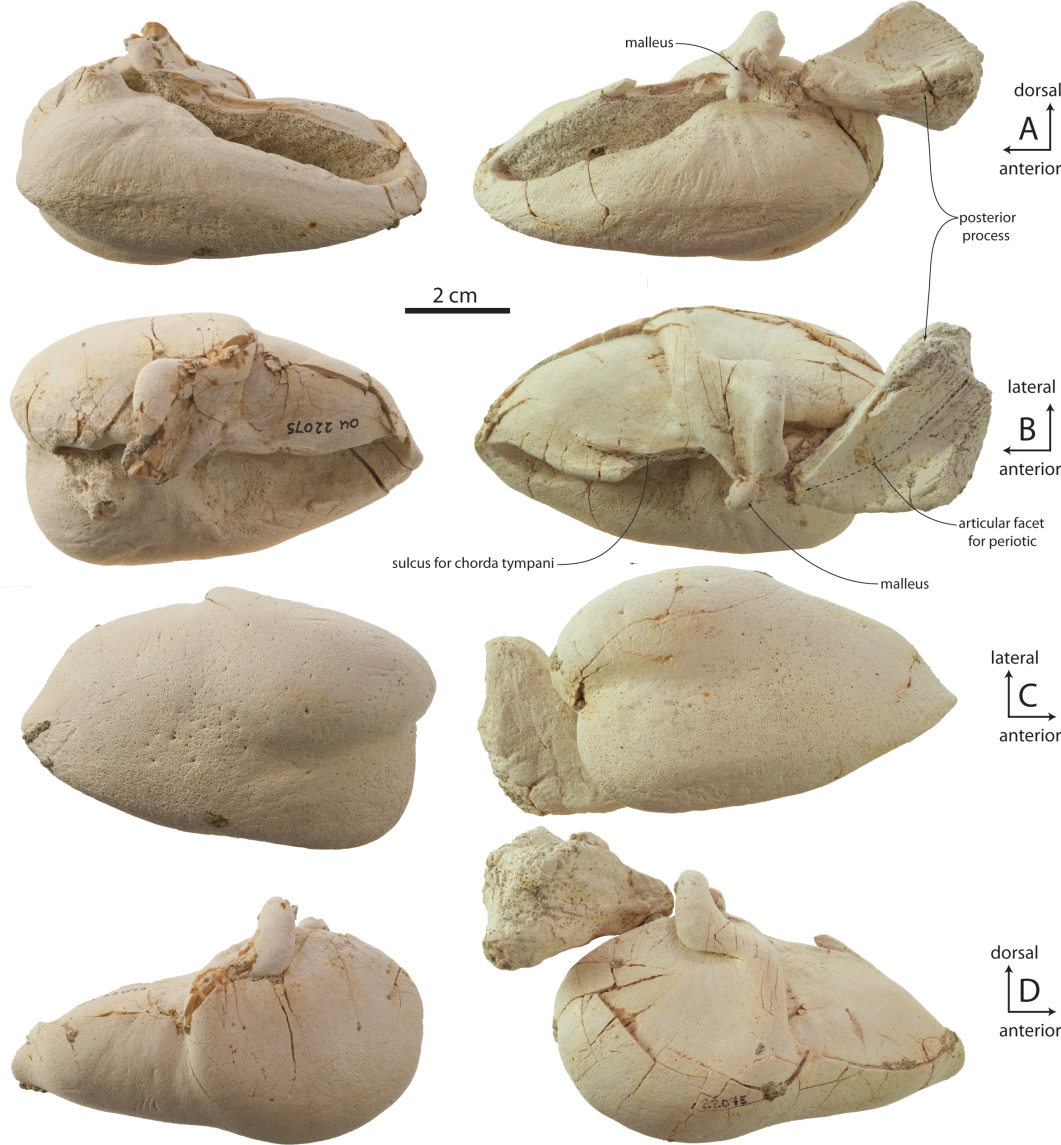
Tympanic bullae of small juvenile of *Waharoa ruwhenua* (OU 22075), with right malleus and posterior process in articulation. (A) medial view; (B) dorsal view; (C) ventral view; (D) lateral view.

**Figure 19:**
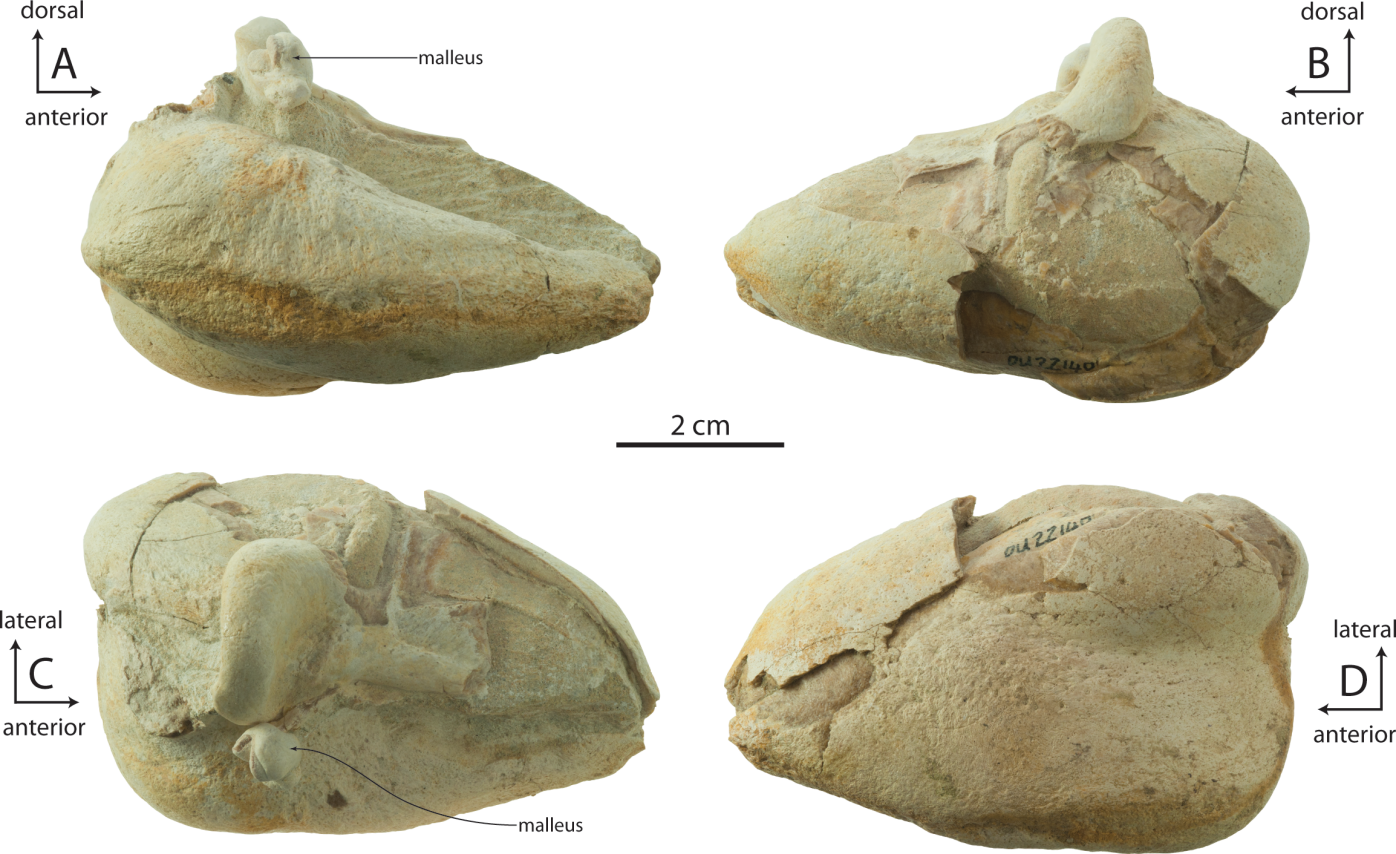
Isolated referred left tympanic bulla of juvenile *Waharoa ruwhenua* (OU 22140) with articulated malleus. (A) medial view; (B) lateral view; (C) dorsal view; (D) ventral view.

The tympanic bulla is relatively small and bears a crushed outer lip and broken, incomplete lateral lobe (Figs. 16–19 and Table 3). The bullae of OU 22044 and OU 22163 are proportionally longer and more anteroposteriorly elongate than OU 22075 and 22140. The involucrum is gracile and dorsoventrally shallow; in dorsal view, the involucrum transversely tapers anteriorly. The inner posterior pedicle is developed as a swollen tubercle. The lateral lobe is damaged, but appears to have extended further posterior than the medial lobe as in OU 22075, 22140, and 22163. A distinct ventromedial ridge is developed on the medial lobe, and in the anterior 1/3 of the bulla is confluent with the involucral ridge. The involucral ridge is discernable along the ventral edge of smooth, faintly striated bone; along the posterior two-thirds of the involucrum the involucral ridge is dorsally retracted from the ventromedial ridge. In all four specimens, a rough, oval and medially oriented facet is developed along the posterior half of the ventromedial ridge; this facet would have approached the lateral edge of the basioccipital crest in life. In medial view, the outer posterior prominence (= posterior portion of lateral lobe) is visible descending below the inner posterior prominence (= posterior portion of medial lobe). In dorsal or ventral view, the median furrow of all four specimens appears lack a deeply incised groove as in *Eomysticetus whitmorei* and *Tokarahia kauaeroa* (Figs. 16–19). In posterior view, the medial lobe bears a ventromedially trending transverse crest. In posterior view, the medial and lateral lobes of the holotype and OU 22163 are approximately equal in transverse width, but in OU 22075 and OU 22140 the medial lobe is slightly transversely wider than the lateral lobe, similar to the condition in basilosaurids, aetiocetids, mammalodontids, and the eomysticetid *Tohoraata raekohao* (Boessenecker & Fordyce, 2015b). The elliptical foramen is broken in the holotype, but open in OU 22163, 22075, and 22140 as a V-shaped incision between the conical process and the inner posterior pedicle. A faint oblique ridge just posterior to the conical process marks the position of the reduced outer posterior pedicle in OU 22163 and 22075.

**Table 3:** Measurements of tympanic bullae of *Waharoa ruwhenua*, in mm to nearest 0.01 mm.

Measurement	OU 22140	OU 22075	OU 22163	OU 22044
Greatest anteroposterior length	70.79	77.93	87.84	88.26*
Greatest transverse width	–	46.55	48.36	50.70
Dorsoventral depth of involucrum anterior to inner posterior pedicle	26.99	28.72	31.06	32.32
Dorsoventral depth at sigmoid process	42.56	44.70	51.45	–
Anteroposterior length of tympanic cavity	63.33	67.02	73.29	73(e)
Anteroposterior length of tympanic cavity anterior to malleus	45.09	49.43	49.11	51.93
Anteroposterior length, dorsal lateral furrow to posterior edge of lateral lobe	38.29	39.17	48.06	46.7*
Transverse width of lateral lobe	–	20.35	23.41	–
Transverse width of medial lobe	23.48	23.10	22.67	25.18
Anteroposterior length between posterior edge of elliptical foramen and anterior edge of inner posterior pedicle	17.63	23.0(e)	18.22	–
Transverse width of sigmoid process	21.52	21.16	22.52	24.42
Length of posterior process	–	43.90	–	45.32

**Notes.**

(e) denotes estimated measurement; asterisk (*) denotes incomplete measurement as preserved.

The anterior part of the conical process is obscured dorsally by the sigmoid process, which extends far medial to the conical process. A fossa on the ventral side of the sigmoid process bears a blind lateral end, and is separated from the tympanic cavity by a horizontal shelf. The sigmoid fissure forms a horizontal cleft anteroventrally. In OU 22075, a posteriorly directed tongue-shaped flange is positioned on the medial edge of the outer lip and approximately halfway between the sigmoid process and the anterior margin of the bulla (Fig. 18B); the sulcus for the chorda tympani transitions from a medially facing groove posteriorly to a dorsally positioned shallow groove anteriorly, dorsally distinguishing the flange from the outer lip of the bulla. A broken surface anterior to the chorda tympani sulcus is present instead in the holotype, OU 22163, and the left bulla of OU 22075; the relationship between the accessory ossicle and this flange are unclear, but both may be homologues of the fused anterior pedicle in Crown Mysticeti.

The lateral furrow is dorsally shallow and filled with matrix, and is otherwise crushed in OU 22044 (Figs. 16B and 16C). In OU 22163 and 22075, the outer lip is not crushed, and the lateral furrow is developed as a crease between the smoothly convex anterior lobe of the bulla and a vertical transverse ridge just anterior to the sigmoid process (Figs. 17 and 18). The tympanic cavity in the holotype and OU 22163 bears a transverse ridge on its ventral surface at about the level of the sigmoid process; dorsolaterally a second ridge corresponds to the lateral furrow, dividing the cavity into anterior and posterior chambers. The isolated bulla OU 22140 is smaller even than OU 22075 but shares similar proportions (Fig. 19).

### Mandible

**Figure 20:**
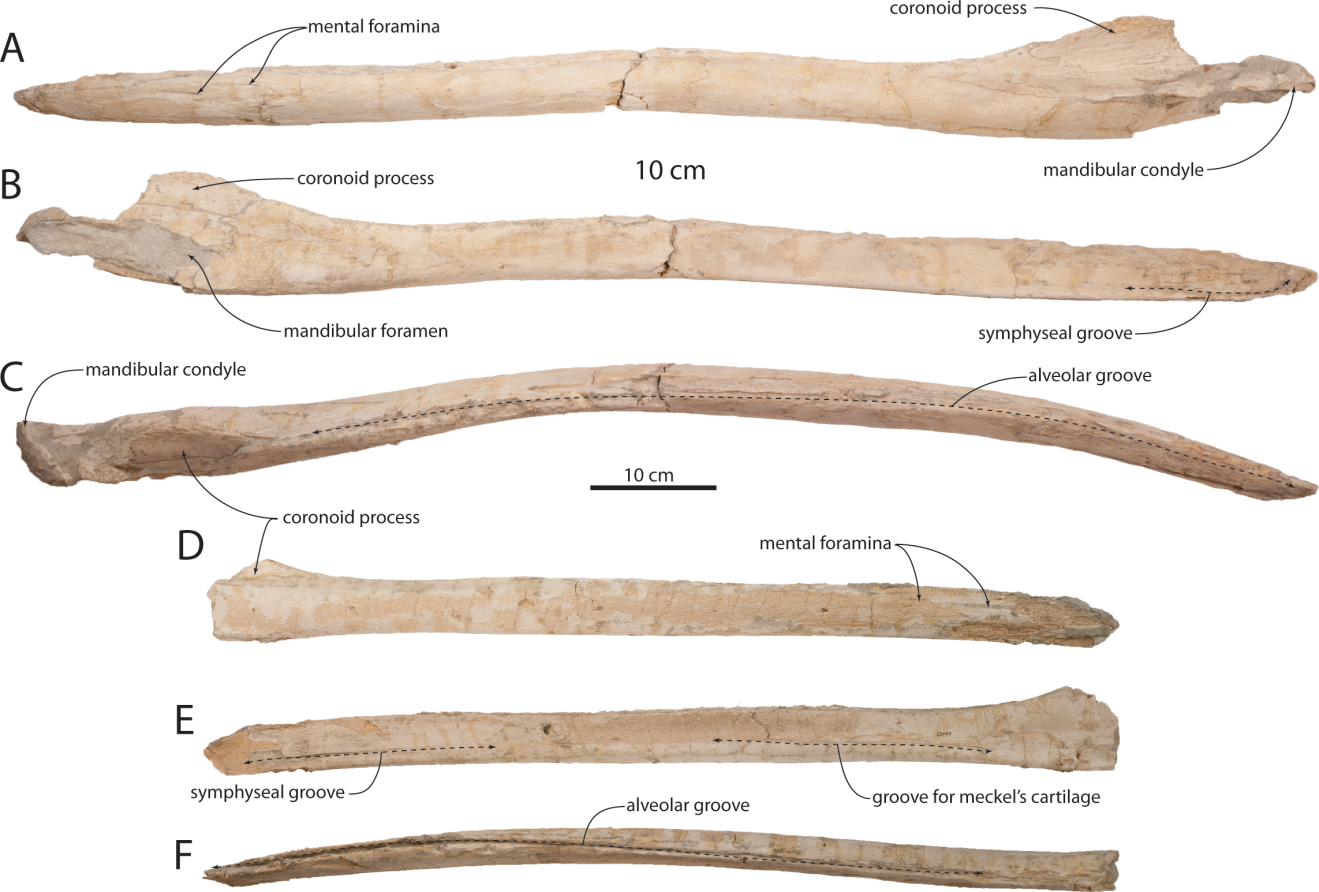
Juvenile mandibles of *Waharoa ruwhenua*. (A) large juvenile (OU 22163) in lateral view; (B) same, medial view; (C) same, dorsal view; (D) small juvenile (OU 22075) in lateral view; (E) same, medial view; (F) same, dorsal view.

The mandible is elongate and slender and possesses parallel dorsal and ventral margins for over 80% of its length (Figs. 3–5, 7, 20 and Table 4). The mandibles of OU 22044 are somewhat distorted, undistorted in OU 22163. In dorsal view, the mandible of OU 22163 is gently laterally bowed and evenly curved along its length so that the mandibular condyle and short neck conform to the curvature of the arc. The horizontal body has an oval cross-section with a slightly flattened medial surface; the ventral margin is smoothly convex in cross-section and lacks a sharp ventral crest. The apex of the mandible is positioned approximately midway between the dorsal and ventral margins; the anterior end of the mandible is acutely pointed and triangular or lanceolate in lateral view, and lacks the dorsoventrally expanded subrectangular profile of some Aetiocetidae, Cetotheriidae, and Balaenopteroidea. A shallow symphyseal groove is developed medially along the anterior portion of the horizontal body; the groove is positioned on the ventral 1/3 of the mandible and is aligned nearly parallel with the ventral margin and gradually descends posteriorly. Anteriorly, the terminus of the groove bends sharply anterodorsally; the groove is accentuated by a subtle elevation positioned immediately dorsal. The symphyseal groove extends posteriorly to a similar degree in OU 22163, but in OU 22075, the groove is developed as a longitudinal cleft in the bone surface and extends posteriorly to the level of the anterior edge of the coronoid process (Fig. 20E). In OU 22075, broken surfaces demonstrate that this fissure extends internally as a physical gap, interpreted here as a retention of the groove for the Meckel’s cartilage into early postnatal ontogeny. Gingival foramina are absent in all specimens.

**Table 4:** Measurements of mandibles of *Waharoa ruwhenua* (in cm, to nearest mm).

Measurement	OU 22075	OU 22163	OU 22044
Total length as preserved	75.7	104.6	173.2
Dorsoventral depth at coronoid process	–	11.8	20.3
Greatest depth anteriorly	4.2	5.0	7.1
Length of mandible anterior to coronoid	65.2	78.9	130.1
Dorsoventral depth of mandibular foramen	–	6.5	13.6
Anteroposterior length of symphyseal groove	10.0	11.6	10.6

A series of mental foramina with elongate anteriorly directed sulci open on the dorsal quarter of the lateral surface of the anterior two-thirds of the horizontal body (Fig. 5); the sulci lengthen anteriorly. Six mental foramina are present on the right mandible; the sulci and foramina are positioned within a longitudinal dorsolateral furrow. The dorsal crest of the mandible is transversely expanded to accommodate a narrow (1 cm wide) vestigial alveolar groove, which is present along the entire horizontal body and terminates at the anterior margin of the coronoid process. Along most of the horizontal body, the groove is shallow and bears many small anteriorly directed foramina and sulci. Anteriorly on the mandible where the dorsal margin descends anteroventrally to the apex, three large anteriorly directed foramina are interpreted as alveoli for the first, second, and third lower incisors (Fig. 6C). Okazaki (2012) interpreted similar foramina along the dorsal margin of the mandible as vestigial alveoli.

The mandible deepens posteriorly immediately anterior to the coronoid process, which is large, tongue-shaped with an arcuate dorsal margin, transversely flattened, and dorsolaterally flaring; the coronoid process is approximately equivalent to the height of the horizontal body and roughly as anteroposteriorly long as deep. In OU 22163, the coronoid process is subtriangular and gradually increases in height posteriorly and has a more steeply oriented posterior margin so that the coronoid apex is positioned in the posterior third of the process; in this regard, OU 22163 primitively retains a plesiomorphic basilosaurid-like condition into early ontogeny. The coronoid process is not preserved in OU 22075. The dorsolateral margin of the mandible is transversely concave at the level of the coronoid process. The mandibular condyle is poorly preserved in the holotype and crushed in OU 22163, but appears to have been posteriorly facing and hemispherical, but excavated anteromedially by the cavernous mandibular fossa. The mandibular foramen bears an arcuate anterior margin positioned roughly at the level of the coronoid apex (Figs. 4 and 20). The angular process is incomplete but appears to have been dorsoventrally plate-like and delicate as in *Yamatocetus* and *Tohoraata raekohao*.

### Atlas

**Figure 21:**
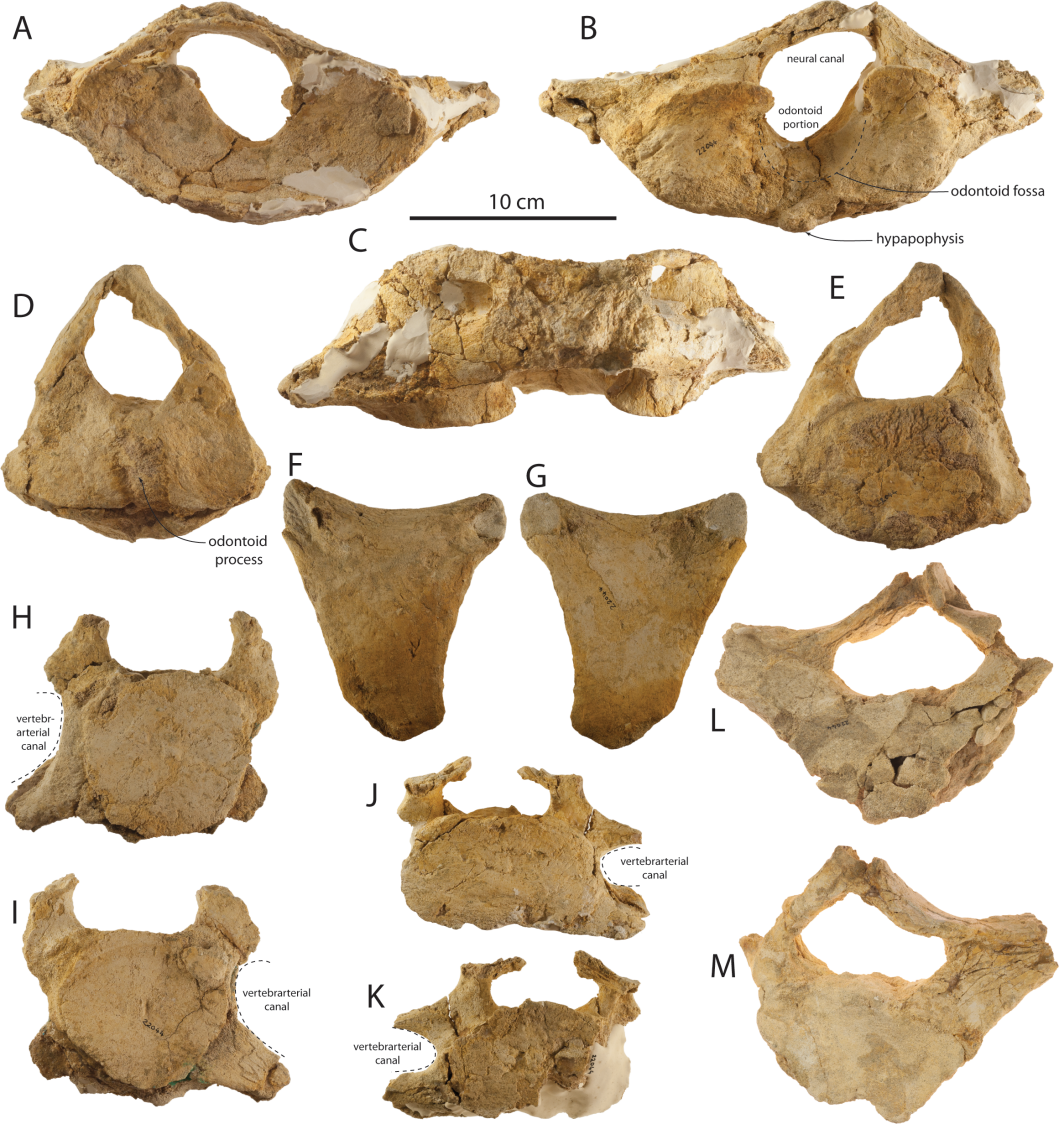
Holotype (OU 22044) postcrania of *Waharoa ruwhenua*. (A) atlas in anterior view; (B) same, posterior; (C) same, dorsal; (D) axis in anterior view; (E) same, posterior; (F) sternum in ventral view; (G) same, dorsal; (H) Ca (possible C3-5) in anterior view; (I) same, posterior; (J) Cb (possible C5-6) in anterior view; (K) same, posterior; (L) thoracic vertebra in anterior view; (M), same, posterior.

**Figure 22:**
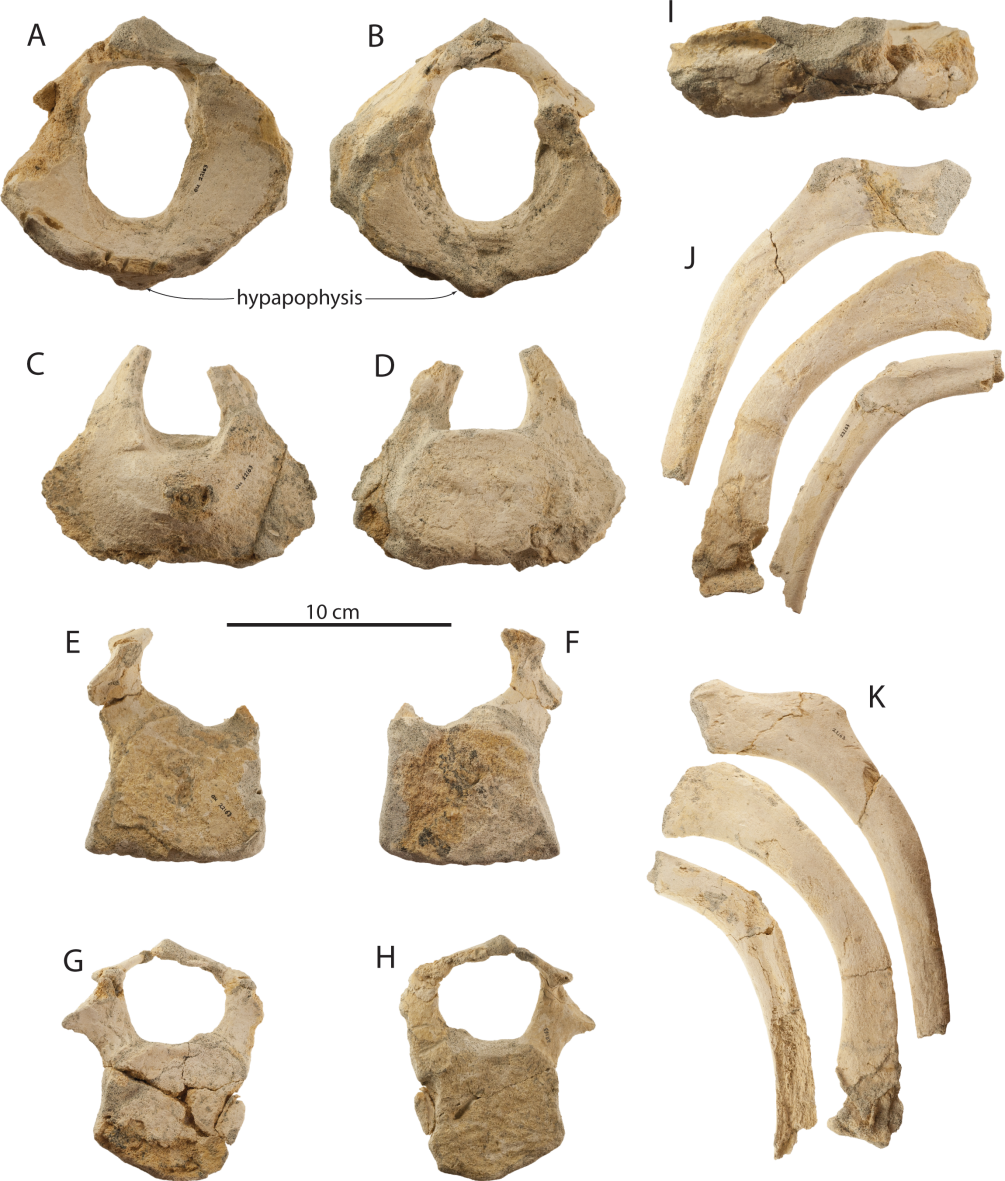
Postcrania of large juvenile (OU 22163) of *Waharoa ruwhenua*. (A–H), cervical vertebrae in anterior (left) and posterior view (right); (I) atlas in dorsal view; (J) ribs in ?anterior view; (K) ribs in ?posterior view.

Atlases are preserved for the holotype (OU 22044) and the large juvenile (OU 22163). The holotype atlas (Figs. 21A–21C and Table 5) is anteroposteriorly thick and in anterior view is oval in outline with the exception of the transverse processes. The oval-shaped vertebral foramen is proportionally small and occupies less than two-thirds of the dorsoventral height of the atlas. The anterior articular surfaces are wide, dorsoventrally deep, transversely concave, and are ventromedially contiguous. In lateral view, the atlas is slightly anteroposteriorly narrower ventrally and widens toward the dorsal margin of the anterior articular surface; the condyloid and axial margins are at a slight angle. The transverse process is relatively small and subconical in shape. The posterior articular surfaces are flat to slightly transversely convex and bear a shallow odontoid fossa immediately ventral to the vertebral foramen. Tubercles for the transverse ligament constrict the vertebral foramen, dividing it into the odontoid portion (ventrally) and the neural foramen (dorsally) of approximate equal dorsoventral depth. The neural arch is small, dorsoventrally thin, lacks an obvious neural spine, and is anterolaterally perforated by a pair of transverse foramina. In comparison to other eomysticetids (*Eomysticetus whitmorei*, *Tokarahia kauaeroa*, *Tokarahia lophocephalus*), the atlas of *Waharoa ruwhenua* is relatively small and gracile. The atlas of OU 22163 (Fig. 22A and Table 5) differs in being anteroposteriorly shorter than the holotype adult, having less deeply concave anterior articular facets, and a dorsoventrally deeper and proportionally larger vertebral foramen. A posteroventrally positioned small hypapophysis is present in OU 22044 and 22163.

**Table 5:** Vertebral measurements of *Waharoa ruwhenua* (in mm, to nearest mm).

Element	Body, a/p length	Body, transverse width	Body, d/v depth	Width of vertebra at transverse process	Vertebral foramen, transverse width	Vertebral foramen, d/v depth
OU 22044 C1	73	161	86	236	57	54
OU 22044 C2	51	128	82	–	34	38
OU 22044 C_a_	29	90	79	–	58	–
OU 22044 C_b_	38	48	–	–	54	–
OU 22044 T?	38	58	–	–	71	41
OU 22163 C1	34	126	93	–	48	67
OU 22163 C2	38	59	65	–	34	–
OU 22163 C_a_	16	61	62	–	44	35
OU 22163 C_b_	18	68	70	–	28	–
OU 22075 C2	32	152	–	–	24	–
OU 22075 C7	12	72	71	140	66	33
OU 22075 T1	19	76	61	143	64	38
OU 22075 T2	30	99	61	106	34	37
OU 22075 T3	27	86	44	119	52	29
OU 22075 T4	26	84	45	106	48	31
OU 22075 T5	24	88	53	128	56	32
OU 22075 T?	27	59	56	112	42	36
OU 22075 L_a_	44	86	54	–	–	–
OU 22075 L_b_	34	87	74	–	38	27
OU 22075 L_c_	36	95	79	–	32	28
OU 22075 L_d_	37	84	66	146	31	–
OU 22075 Ca1-2	34	97	86	208	20	22

**Notes.**

a/p anteroposterior d/v dorsoventral depth

### Axis

**Figure 23:**
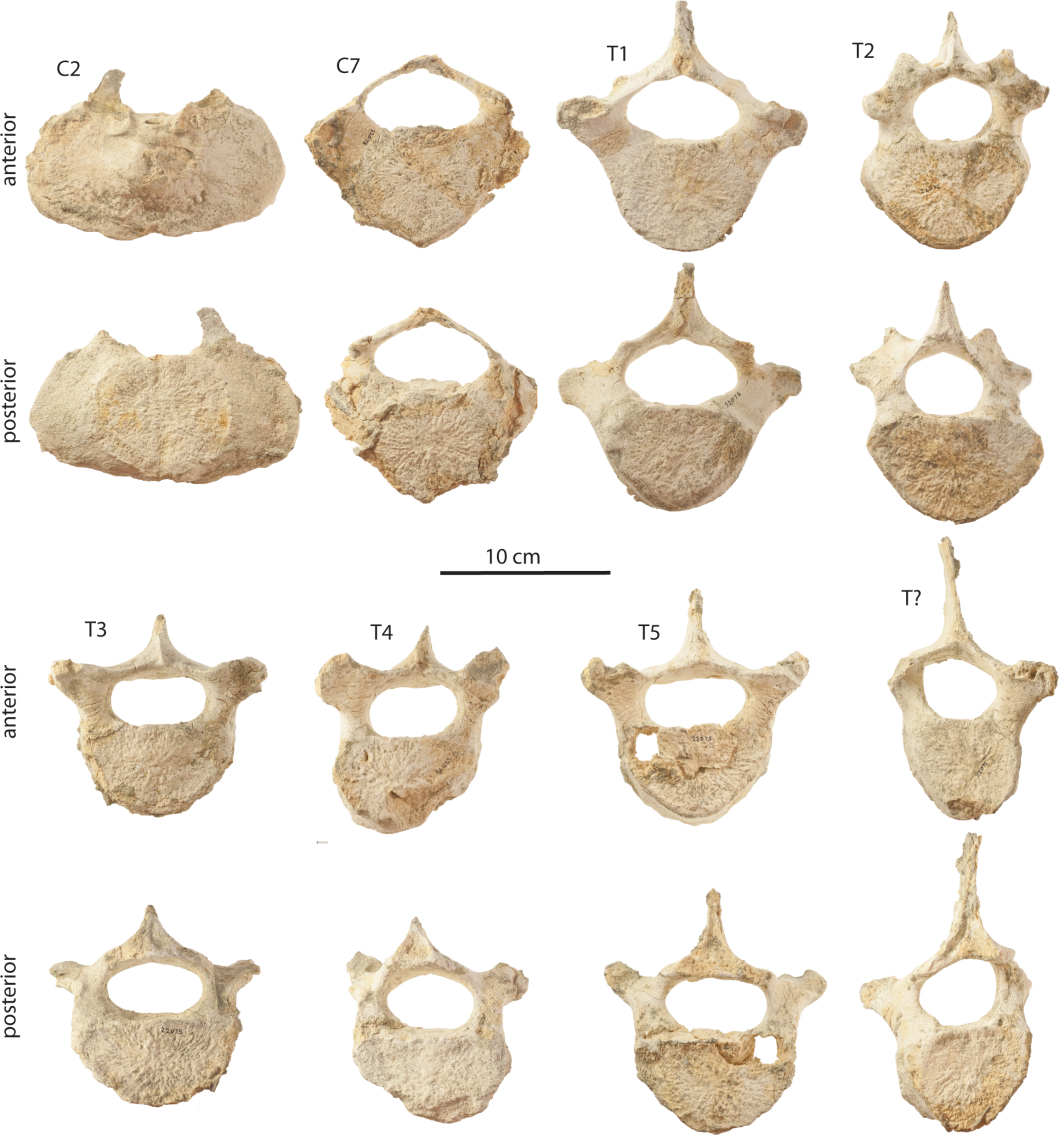
Cervical and thoracic vertebrae of small juvenile (OU 22075) of *Waharoa ruwhenua*. C denotes cervical, T denotes thoracic; see description for vertebral number assignment.

Axes are preserved for the holotype (OU 22044) and both juveniles (OU 22163 and 22075). The holotype axis (Figs. 21D, 21E and Table 5) is poorly preserved and is missing the transverse processes and neural spine. The odontoid process is low, transversely broad, and convex; the anterior articular surface is otherwise flat. The posterior epiphysis is partially fused and the posterior articular surface is oval. The neural arch is high, transversely narrow, delicate, and defines a large triangular vertebral foramen with a flat ventral margin. The axis of OU 22163 (Fig. 22A and Table 5) possesses an incomplete but dorsoventrally deep neural arch; postzygapophyses are incomplete but do not appear to have projected dorsolaterally as in *Yamatocetus*. The well-preserved axis of OU 22075 (Fig. 23 and Table 5) has a transversely wide oval outline in anterior view with a centrally positioned and low odontoid process with a narrow vertical cleft. In OU 22075, the anterior articular surfaces bear a punctate texture, and the posterior epiphysis is missing. A hypapophysis is not developed in any specimen, and in OU 22075, a shallow ventral median notch is present. Transverse processes do not appear to have been ossified in OU 22075, as smooth surfaces are present laterally rather than fractured transverse process bases.

### C3–C7

Posterior cervical vertebrae (Figs. 21H–21K, 22A, 23 and Table 5) of uncertain position are preserved for the holotype (OU 22044) and the large juvenile (OU 22163), and a C7 is preserved for the small juvenile (OU 22075). Two undetermined cervical vertebrae of the holotype, C_a_ and C_b_, possibly represent a C3–5 and a C5–6 (respectively). These vertebrae exhibit an oval-shaped body with ventrolaterally directed parapophyses and transverse processes defining a small vertebrarterial canal (∼one-half to one-third depth of the body); the body of C_a_ appears to be dorsoventrally shallower than C_b_. Both vertebrae preserve partial neural arches with an anteroposteriorly flattened pedicle, and C_a_ additionally preserves a dorsoventrally flattened and delicate lamina with a shelf-like postzygapophysis defining a dorsoventrally shallow and transversely wide vertebral foramen. The anterior epiphyses of C_a_ and C_b_ are fully fused and the posterior epiphyses are partially fused. Two cervical vertebrae possibly representing C3-6 are preserved in OU 22163, and differentiated as C_a_ and C_b_; these differ from the holotype cervicals in being more anteroposteriorly flattened, and C_a_ of OU 22163 bears a body that is near pentagonal and equilateral in shape. C_a_ and C_b_ of OU 22163 appear to have possessed dorsoventrally deeper vertebrarterial canals, a transversely narrower pedicle, and a nearly equilateral subcircular vertebral foramen. Cervical epiphyses of OU 22163 are unfused. The C7 of OU 22075 (Fig. 23) is similarly anteroposteriorly flattened and also exhibits a pentagonal body with a pointed ventral apex but lacks a hypapophysis. The C7 differs from other posterior cervicals in lacking a vertebrarterial canal and possessing a large, subrectangular, anteroposteriorly flattened and plate-like transverse process. The delicate lamina defines a subtriangular and relatively large vertebral foramen. Both epiphyses are unfused.

### Thoracic vertebrae

A single thoracic vertebra is preserved for the holotype adult (OU 22044), and two partial, unprepared thoracic vertebrae are preserved in the large juvenile (OU 22163); OU 22075 preserves six thoracic vertebrae including T1, T2, T3?, T4?, T5?, and a posterior thoracic of uncertain position (Figs. 21L, 21M, 23 and Table 5). T1 of OU 22075 is somewhat similar to C7 in proportion and in possessing transverse processes positioned at or just above the dorsal margin of the body, but differs in having a circular and slightly anteroposteriorly thicker body, a more oval-shaped vertebral foramen, tubercular prezygapophyses on the transverse processes, a more robust (but still anteroposteriorly thin) pedicle, and a short but distinct neural spine. The transverse processes are slightly anteriorly inclined, and the neural arch is delicate but possesses a less flattened lamina than C7. Shelf-like postzygapophyses are present and project somewhat dorsally so that they are visible on the dorsal margin of the lamina in anterior view. Bilateral fossae are present on the posterior surface of the neural spine and medial portion of the lamina. T2 of OU 22075 (Fig. 23) is similar in size to T1 but differs principally in having an anteroposteriorly thicker body and nearly cylindrical, robust pedicles which are positioned dorsally so that the widest part of the body is positioned ventrolateral to the pedicles. The neural arch encircles a small, circular vertebral foramen. The transverse processes are more robust and elevated than in T1, with large tubercle-like prezygapophyses positioned immediately dorsomedial to the transverse process. The lamina is thick and possesses medially placed postzygapophyses near the posteroventral terminus of the neural spine. T3?, T4?, T5?, and T? of OU 22075 (Fig. 23) differ from T2 in being somewhat anteroposteriorly thicker, lacking obvious prezygapophyses, possessing a transverse process that is more dorsally elevated and anteriorly thrusted, and a pedicle that is more dorsoventrally elongate, and an anteroposteriorly longer lamina. All thoracic vertebrae of OU 22163 and OU 22075 have unfused epiphyses.

### Lumbar vertebrae

**Figure 24:**
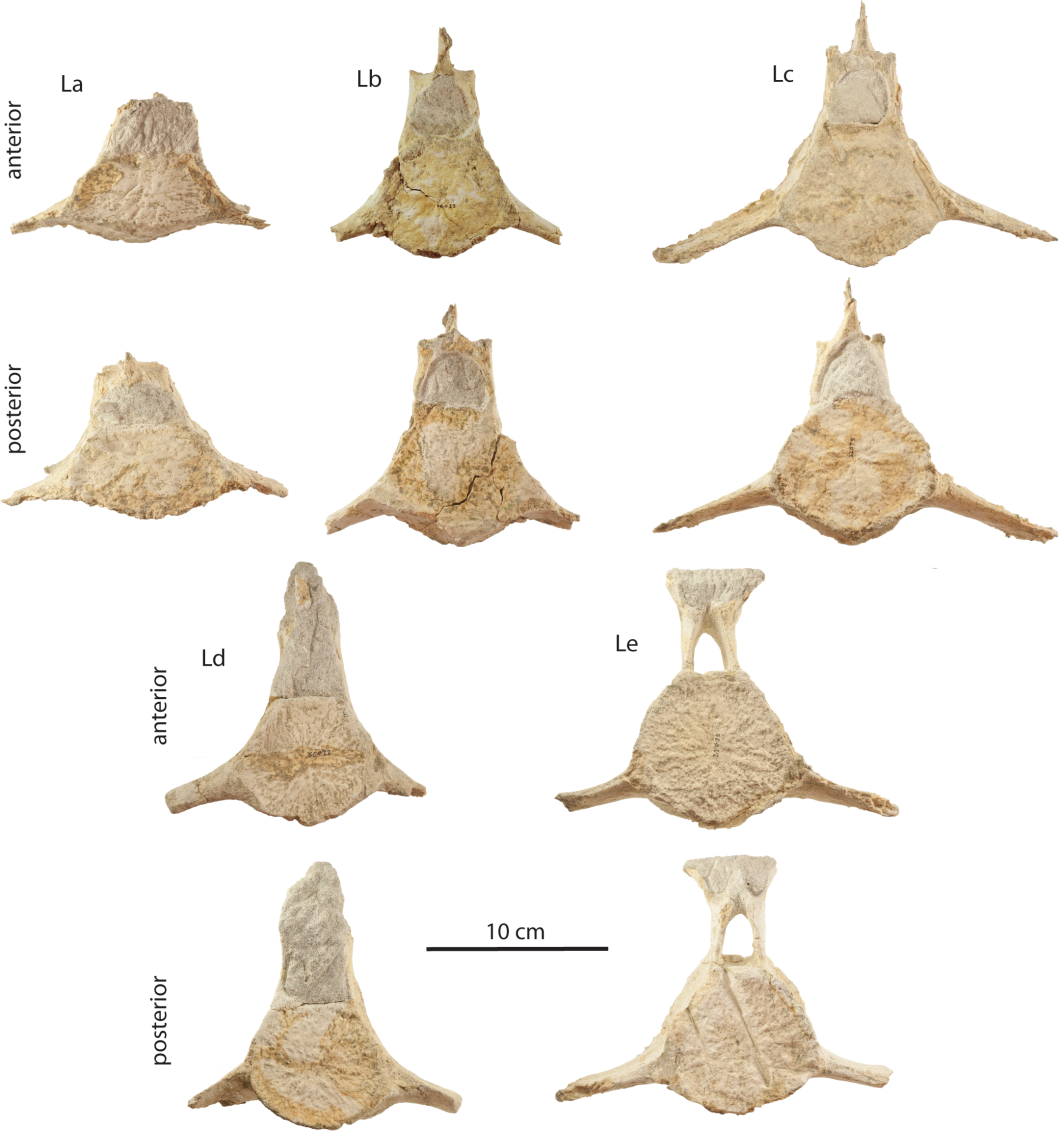
Lumbar vertebrae of small juvenile (OU 22075) of *Waharoa ruwhenua*. Letter arbitrarily denotes hypothesized relative anteroposterior position, with La being furthest anterior and Le being furthest posterior in column.

Four nearly complete lumbar vertebrae of uncertain position and several additional fragments are preserved in OU 22075 (Fig. 24 and Table 5). These vertebrae are characterized by a pentagonal body that is anteroposteriorly thicker than the posterior thoracic vertebrae, and ventrally positioned, dorsoventrally flattened, elongate, and ventrolaterally oriented transverse processes. The neural arch is much narrower than the body and defines a small subrectangular vertebral foramen. Small prezygapophyses are present dorsally and are dorsally directed; the neural spine is transversely narrow and dorsally short, with postzygapophyses reduced to roughened facets on its posteroventral terminus. One posterior lumbar differs from the others in having a larger and more circular body, a transversely narrower neural arch with a short neural spine and smaller vertebral foramen, and by possessing dorsolaterally flaring prezygapophyses. This vertebra is morphologically similar to a tentatively identified anterior caudal in *Eomysticetus whitmorei* (Sanders & Barnes, 2002: Fig. 20B) but lacks obvious ventral processes for articulation of a chevron and is thus better interpreted as a posterior lumbar.

### Ribs

**Figure 25:**
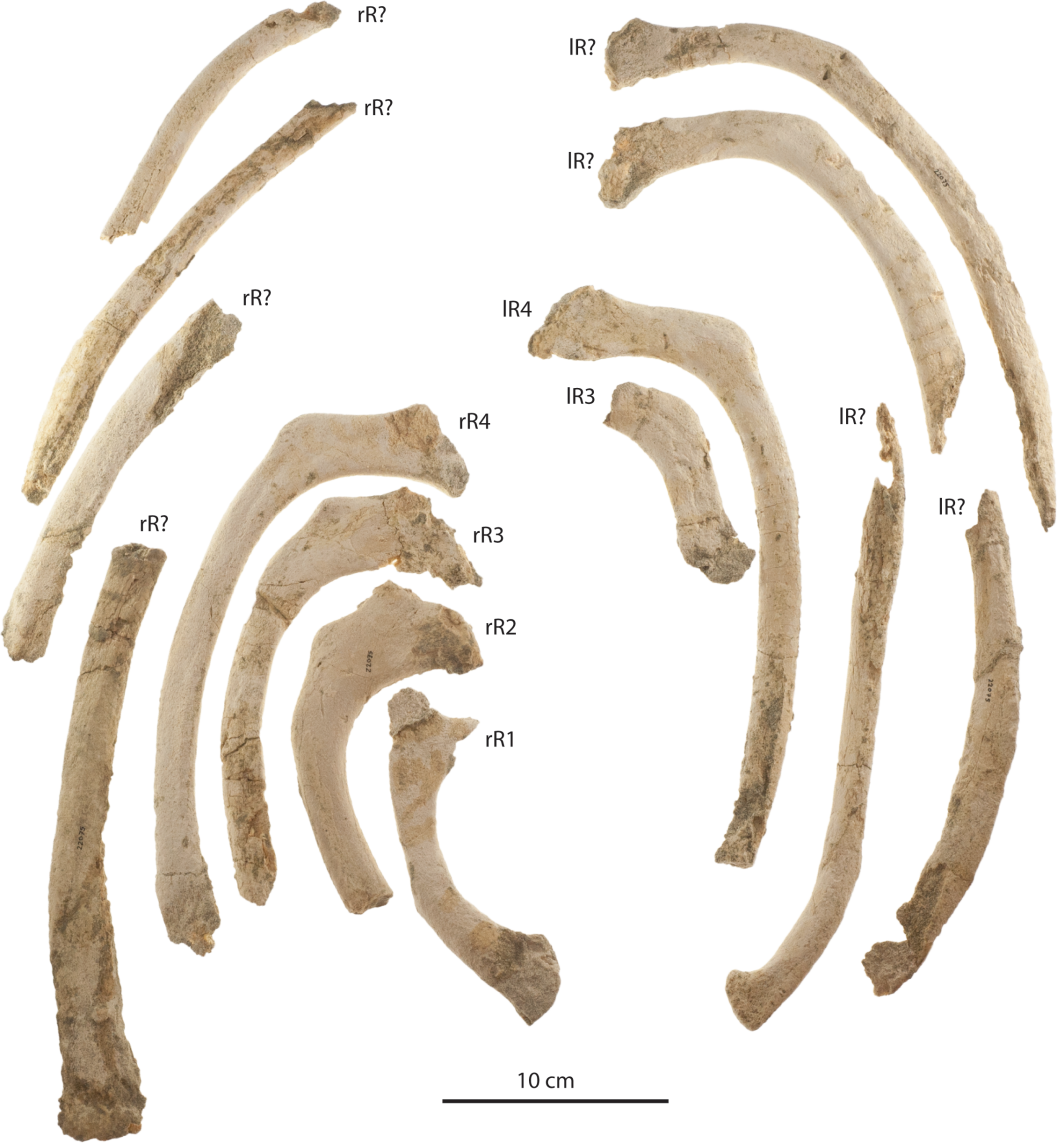
Left (lR) and right (rR) ribs of small juvenile (OU 22075) of *Waharoa ruwhenua* in anterior view.

Several partial ribs of uncertain position (Figs. 22C and 22D) are preserved with the holotype adult (OU 22044) and large juvenile (OU 22163). Some preserve a dorsoventrally expanded and anteroposteriorly flattened proximal end; rib shafts of OU 22044 and 22163 are narrow and have an oval cross section. The small juvenile (OU 22075) preserves parts of 16 ribs, including right R1, right R2, left R3, left R4, and various other ribs of uncertain position (Fig. 25). R1–R4 possess a dorsoventrally expanded and anteroposteriorly flattened, subrectangular proximal end. R1 lacks a secondary tubercle but possesses a tubercle positioned dorsal to the incomplete capitulum. R2–R4 possess a dorsolaterally positioned secondary tubercle that is further distally positioned and separated from the tubercle in sequentially posterior ribs. R1–R4 are relatively short and highly curved, and progressively become longer and straighter; isolated ribs and fragments lacking a proximal end are much longer and straighter and presumably represent posterior ribs. The distal end of R1 is transversely flared and bears a convex articular facet; at least one posterior rib bears a distal but unexpanded facet, and the articular surface of both ribs bears a punctate surface texture, likely for cartilage. All ribs of all three specimens lack obvious pachyostotic inflation.

### Sternum

A sternum is present only in the holotype (OU 22044; Figs. 21F and 21G). The triangular sternum consists of a single element and is dorsoventrally flattened. The anterior and lateral margins are concave. Anteriorly, a pair of bilateral fossae are present on the ventral side of the anterolateral tips for articulation with a single pair of ribs. In lateral view, the sternum is dorsally concave and ventrally convex; posteriorly the sternum tapers to a rectangular sheet without any apparent facets for additional ribs or other sternal elements. The sternum is 109 mm wide and is 120 mm long. In dorsal and ventral view the sternum is somewhat asymmetrical, although this may be caused by diagenetic deformation.

### Scapula

**Figure 26:**
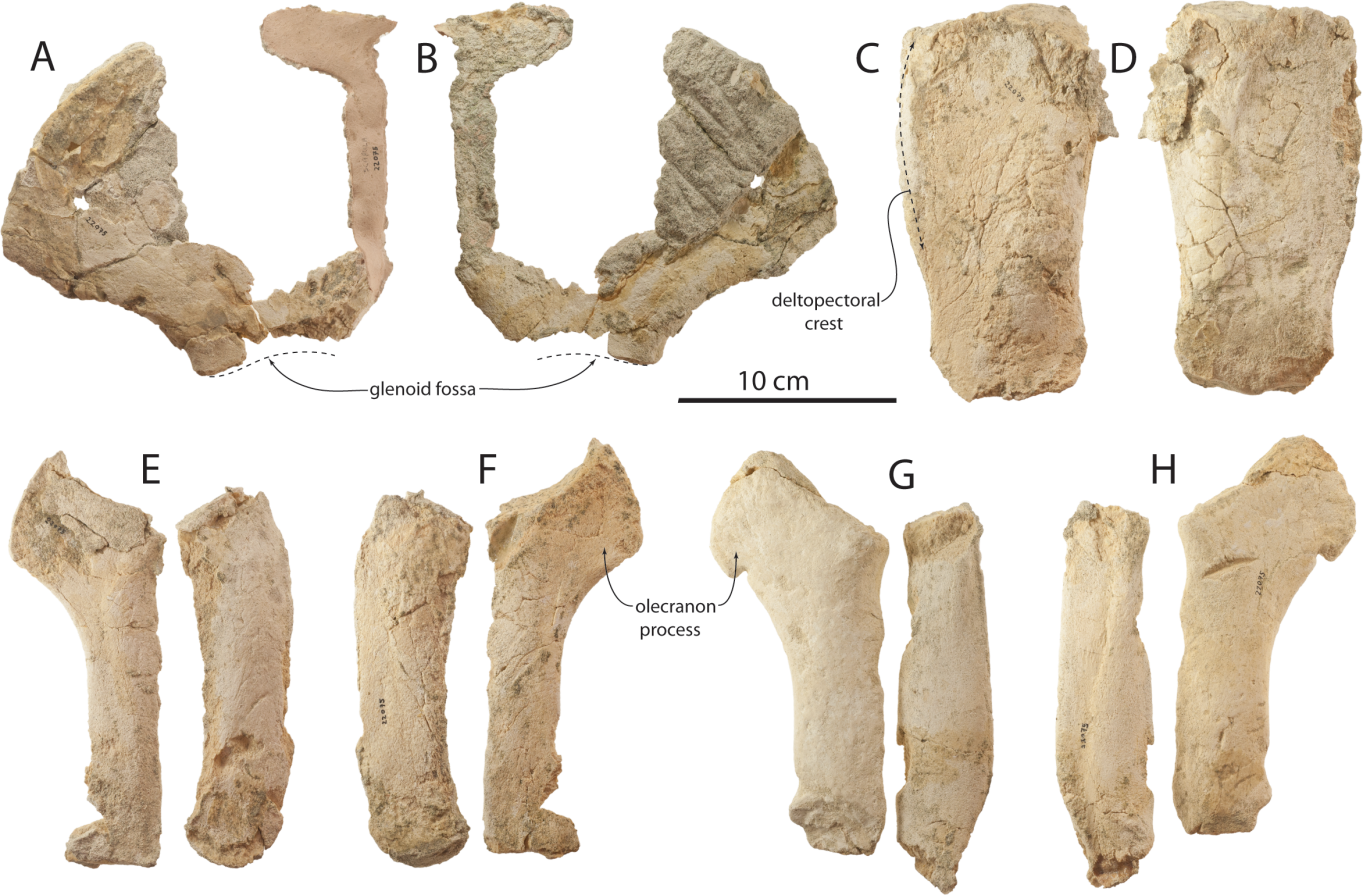
Forelimb elements of small juvenile (OU 22075) of *Waharoa ruwhenua*. (A) scapula, medial or lateral; (B) scapula, reversed; (C) left humerus, lateral; (D) left humerus, medial; (E) left ulna and radius in medial view; (F) same, lateral view; (G) right ulna and radius in lateral view; (H) same, medial view.

A fragmentary scapula is preserved in OU 22075; it lacks the distal end and anterior border, and it is unclear which side it represents (Figs. 26A and 26B). The dorsal border is arcuate with a slightly concave posterior margin, similar in outline to the holotype scapula of *Tokarahia kauaeroa*. As in other eomysticetids, the posterodorsal corner of the scapula lacked a pointed apex. Damage has removed any evidence of the acromion and coracoid processes.

### Humerus

Partial humeri are preserved for OU 22075, including a left humeral diaphysis and a fragmentary right humerus (Figs. 26C, 26D and Table 6). The shaft is transversely compressed and subrectangular in lateral view; the humerus is slightly transversely thicker posteriorly, but also appears to be diagenetically compacted. The deltopectoral crest occupies three-quarters of the length of the diaphysis. The proximal and distal epiphyses are unfused and missing, but the shape of the distal epiphyseal surface is suggestive of two articular facets offset by an angle (as in *Tokarahia kauaeroa* and *Yamatocetus*) rather than a continuous arcuate surface as in archaeocetes. The proportions of the humerus in lateral view is shorter in comparison to *Yamatocetus* and *Eomysticetus*. No obvious muscle attachments or olecranon fossa are present, perhaps owing to the young age of OU 22075.

**Table 6:** Measurements (in mm) of forelimb elements of small juvenile of *Waharoa ruwhenua* (OU 22075).

Measurement	OU 22075, left	OU 22075, right
Humerus, length of diaphysis	159	–
Humerus, anteroposterior depth at deltopectoral crest	81	–
Humerus, greatest transverse width	27	–
Radius, greatest length	152	153
Radius, greatest transverse width	16	36
Radius, anteroposterior depth at distal end	39	26
Radius, anteroposterior depth at proximal end	40	26
Ulna, greatest length	171	164
Ulna, length of diaphysis to humeral articulation	133	130
Ulna, anteroposterior length at olecranon process	64	73
Ulna, anteroposterior depth at distal end	39	46
Ulna, greatest transverse width	14	24

### Radius

Complete left and right radii are preserved in OU 22075 (Figs. 26E–26H and Table 6). The right radius is transversely wider than the left and composed of denser, osteosclerotic bone; the surficial texture of the right radius is less spongy than the left. The shaft is transversely narrow with a lensoidal cross-section and a sharp interosseous crest; on the right radius, a distinct longitudinal furrow is developed anteriorly along the distal half of the medial surface. The radius is nearly straight but gently anteriorly bowed; the radius is slightly anteroposteriorly broader in its distal half and becomes narrower toward the distal end. The radius is transversely narrower proximally and thickens distally.

### Ulna

Complete left and right ulnae are preserved for OU 22075 (Figs. 26E–26H and Table 6). Like the radius, the right ulna is also transversely inflated and composed of dense osteosclerotic bone, while the left ulna is more gracile and composed of less dense spongy bone typical of the rest of the skeleton; this suggests a pathologic condition for the right forelimb. The shaft is transversely compressed with an oval-lensoidal cross-section and a sharp interosseous crest, and slightly widens distally in the left ulna. Unlike the radius, the shaft is straight in lateral view. The olecranon process is developed as a fan-shaped process that is proximally subtriangular in the left ulna and more bluntly shaped in the right ulna. The distal olecranon does not form a posterior or distal apex in either ulna, but on the right ulna a distinct notch (as in *Yamatocetus*; Okazaki, 2012: Fig. 26) is developed near the distal termination of the olecranon process. On the left ulna, the shaft gradually transitions to the olecranon process. The humeral articular facet is transversely flat but anteroproximally concave in lateral view; the facet faces dorsomedially.

### Rib histology

**Figure 27:**
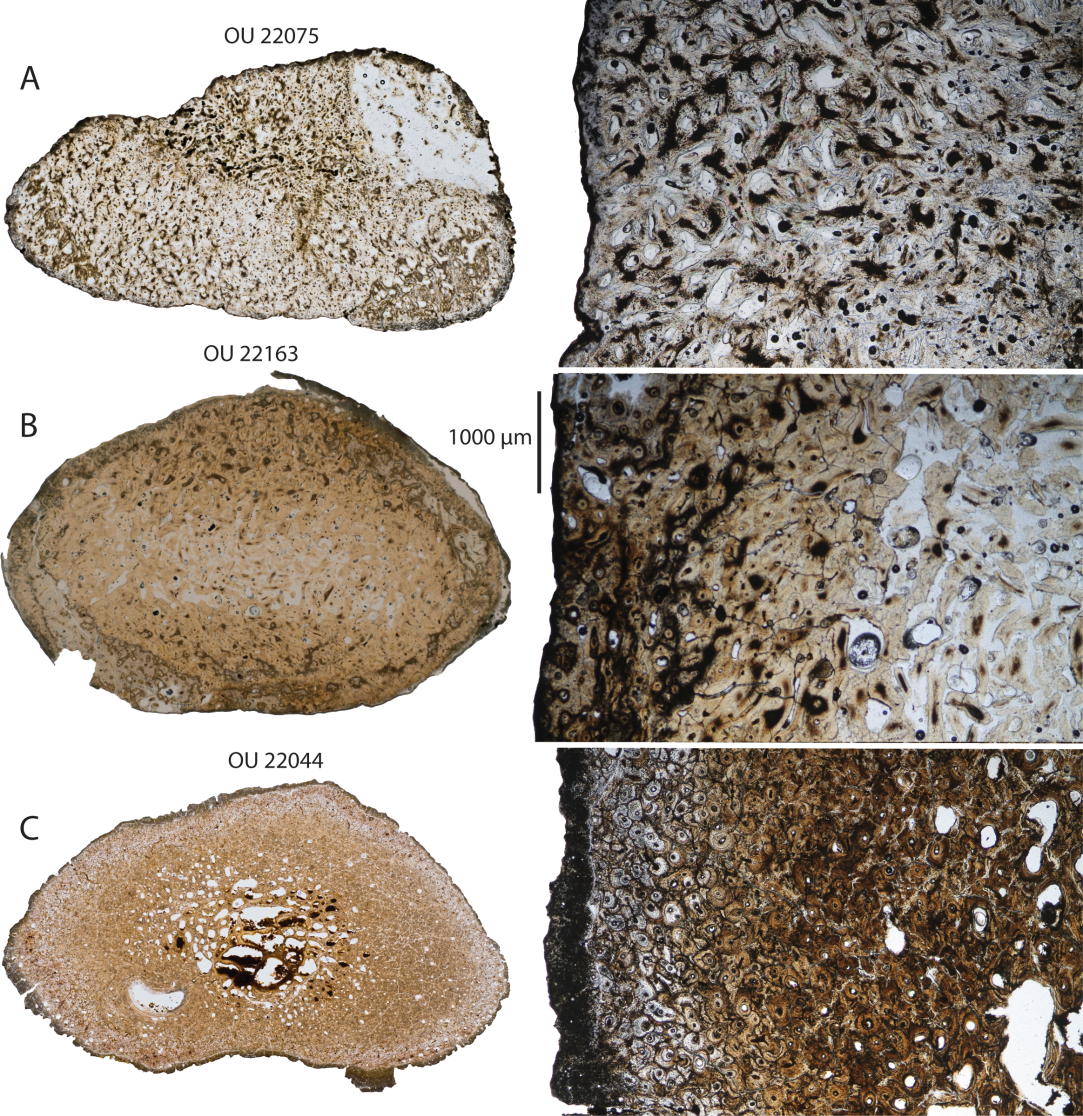
Osteohistology of *Waharoa ruwhenua* ribs. (A) small juvenile (OU 22075); (B) large juvenile (OU 22163 slide B); (C) holotype adult (OU 22044). Whole cross-sections in left column, photomicrographs in right column.

Four ribs were sectioned for histological examination (Fig. 27), including one rib from OU 22044 and 22075 and two for OU 22163, one of which (slide A) was sufficiently altered as to prohibit ready microscopic examination and require a second rib to be sectioned (slide B). All sections were taken from incomplete ribs and thus cardinal anatomical orientation (anterior, posterior, medial, lateral) are unknown.

The smaller juvenile (OU 22075) has a subtriangular rib with a thick cortex and no discrete medullary zone (Fig. 27A). In contrast, the larger juvenile (OU 22163) has a thick cortex (approximately 1.5–2 mm thick in slide A, and 2–3.5 mm in slide B) and an oval medullary zone with its long axis parallel to that of the rib cross section (Fig. 27B); no discrete single cavity is developed, but there is a series of large, separated longitudinal vascular channels (325–565 µm diameter). The cross-section of the adult (OU 22044; Fig. 27C) is similar to that of OU 22163 but with absolutely larger longitudinal vascular channels (200–1,500+ µm diameter). Both juveniles (OU 22075 and 22163) possess outer cortices that are composed nearly entirely of highly disorganized woven bone. In OU 22075, the entire cortex is composed of woven bone with radially oriented vascular channels, with a transition to longitudinal vascular channels towards the center. In OU 22163, primary osteons are oriented generally longitudinally but some radial vascular channels exist, although the extreme radial architecture that characterizes OU 22075 is not present. The outermost cortex of OU 22163 includes sparse longitudinally oriented secondary osteons (110–260 µm diameter); they are typically separate and only rarely cross-cut one another. The inner cortex of OU 22163 is entirely composed of woven bone. Most trabeculae within the medullary zone are fractured, but some possess endosteal laminae.

The sectioned rib of OU 22044 (Fig. 27C) is reniform in cross-section and is missing most of the periosteum; the cortex is proportionally thicker (approximately 3.5–6 mm, adjusted for missing periosteum) and the oval-shaped medullary zone is proportionally smaller than in OU 22163 but similarly composed of separate longitudinal vascular channels. The inner and outer cortices are almost entirely composed of densely overlapping secondary osteons and fragments thereof (90–320 µm diameter). In some regions fragments of non-remodeled lamellar bone exist; osteocyte lacunae here are parallel with circumferential lamellae. Three or more dark bands, potentially representing lines of arrested growth (LAGs), are present within these interstitial fragments of lamellar bone. The inner cortex of OU 22044 includes somewhat larger secondary osteons (200–365 µm) and vascular channels of similar diameter (250–370 µm) with endosteal lamellae, the former likely representing vascular channels nearly completely filled by endosteal lamellae. Trabeculae within the medullary zone consist primarily of endosteal lamellae formed circumferentially around their respective vascular channels.

## Comparisons

*Waharoa* possesses several features which unequivocally identify it as an eomysticetid, including an extremely elongate rostrum, extremely elongate nasals, frontal with anteromedial projection, high sagittal crest, longitudinally rotated zygomatic processes that extend anterior to the occipital shield and lack supramastoid crests, distinct secondary squamosal fossa, and a discontinuous and low superior process of the periotic with anterior and posterior apices. Owing to possession of clear eomysticetid features and phylogenetic placement of *Waharoa*, comparisons are restricted to other Eomysticetidae. *Waharoa* shares with *Tokarahia* a nasal that is laterally overlain by and sutured to the premaxilla (unknown in *Tohoraata*). The frontal is anteroposteriorly shorter than *Yamatocetus*, but similar to *Eomysticetus*, *Tohoraata*, and *Tokarahia*; unlike the northern hemisphere taxa, *Waharoa* possesses a series of foramina with elongate and radiating sulci in the supraorbital process of the frontal. Sutural surfaces potentially indicate that the premaxilla extended slightly posterior to the nasal, unlike all other eomysticetids. The zygomatic process of *Waharoa*, like *Tokarahia*, is medially bowed, differing from the condition in *Eomysticetus*, *Micromysticetus*, and *Yamatocetus*; the squamosal of *Waharoa* further differs from the northern hemisphere eomysticetids in possessing a trough-like shallow secondary squamosal fossa with a medial ridge. The periotic of *Waharoa* shares a secondary tuberosity of the anterior process with *Tohoraata*, to the exclusion of all other eomysticetids; the concave anterodorsal margin of the anterior process is shared also with *Tokarahia* and *Tohoraata*. *Waharoa*, *Tohoraata* and *Tokarahia* share a triangular anterior process, unlike the rectangular shape in *Eomysticetus* and *Micromysticetus*. The posterior process of the periotic is relatively shorter than in *Tohoraata* and *Tokarahia*; however, the posterior bullar facet is smooth (unlike *Eomysticetus* and *Micromysticetus*) and transversely convex and lacking two facets (differing from *Eomysticetus* and *Micromysticetus*). The periotic of *Waharoa* is otherwise absolutely smaller than all other eomysticetids. The tympanic bulla of *Waharoa* is similar in overall morphology with most other eomysticetids but is proportionally narrower (transversely) and shallower (dorsoventrally) than all other eomysticetids. The lateral lobe is ventrally inflated and clearly visible in medial view below the medial lobe, unlike almost all other stem mysticetes. It further differs from *Tohoraata* in having lateral and medial lobes of equal transverse width.

The mandible of *Waharoa* is longitudinally straight in lateral view, unlike the downturned mandibular terminus of *Eomysticetus* and *Micromysticetus* and upturned terminus in *Yamatocetus*; like *Micromysticetus* and *Tokarahia* the terminus is lanceolate in lateral aspect, unlike the subrectangular profile in *Yamatocetus*. The atlas exhibits a subtriangular transverse process, unlike the rectangular process in *Tohoraata*, *Tokarahia*, and *Yamatocetus*; medial tubercles form a Fig. 8 shaped vertebral foramen as in *Tohoraata*, but unlike other eomysticetids. The axis lacks dorsolaterally projecting postzygapophyses, like *Tohoraata* and *Tokarahia* but unlike *Micromysticetus* and *Yamatocetus*.

## Phylogenetic Relationships

**Figure 28:**
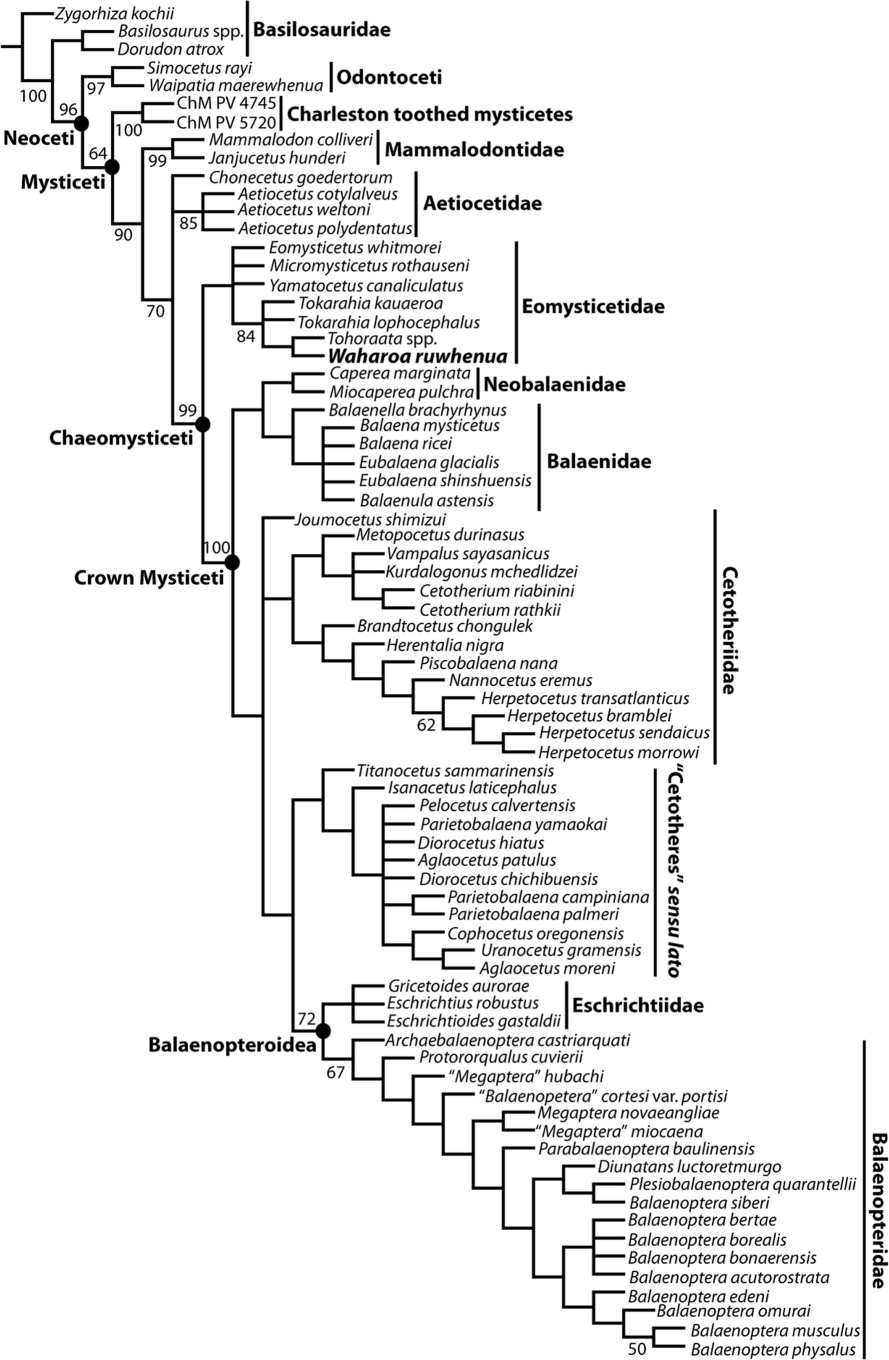
Phylogenetic relationships of *Waharoa ruwhenua*. (A) strict consensus of 88 equally parsimonious trees (1,417 steps, CI: 0.361, RI: 0.812) recovered under equal weighting with bootstrap support values included. (B) single most parsimonious tree (122 steps, CI: 0.357, RI: 0.808) recovered under implied weighting, with bootstrap values included.

Cladistic analysis recovered *Waharoa ruwhenua* as an eomysticetid with strong support (Fig. 28). Eomysticetid monophyly was poorly supported under equal weighting (49%); six synapomorphies supported eomysticetid monophyly, including triangular anteromedial projection of frontal present (77:1), high sagittal crest present (93:0, reversal), supramastoid crest absent on zygomatic process (118:2), zygomatic process with parallel lateral and medial margins (125:1), secondary squamosal fossa present (127:1), and superior process of periotic discontinuous with anterior and posterior apices (161:1). Similar to Boessenecker & Fordyce (in press), a southern hemisphere eomysticetid clade including *Tohoraata*, *Tokarahia*, and *Waharoa* was recovered with strong support (84%); six synapomorphies supported this clade including medially bowed zygomatic processes (132:1), incisural flange present on periotic (168:1), posteroexternal foramen developed as a fissure (175:1), posterodorsal angle of periotic formed as right angle (178:1), concave anterodorsal margin of anterior process of periotic (179:1), and anterior internal acoustic meatus transversely “pinched” by projections of meatal rim (205:1). Resolution was poorer for more exclusive clades; a sister taxon relationship between *Tohoraata* and *Waharoa* was weakly supported (48%), and supported by five synapomorphies including a subvertical to posteroventrally projecting postglenoid process (121:1), alignment of the facial canal, internal acoustic meatus, and aperture for the cochlear aqueduct (153:1), tubercle present laterally on anterior process of periotic (181:1), anterior margin of fenestra rotunda overlaps fenestra ovalis in ventral view (204:1), and shallow suprameatal fossa (218:1). Inclusion of *Waharoa ruwhenua* within the analysis did not affect topology of the remainder of the cladogram, and a more exhaustive discussion of broader mysticete relationships as revealed by this matrix is given in Boessenecker & Fordyce (in press).

## Discussion

### Ontogeny

The available sample of specimens of *Waharoa ruwhenua* permits examination of the ontogeny and osteological development of an archaic mysticete for the first time. Few studies have addressed skeletal ontogeny in extant mysticetes (Bisconti, 2001; Walsh & Berta, 2011; Nakamura, Kato & Fujise, 2012; Nakamura & Kato, 2014), and is a virtually unstudied aspect of fossil mysticetes.

### Cranial sutures, mandibular morphology, and vertebral fusion

Fusion of cranial sutures is frequently used to estimate the age of modern and fossil mammals, including cetaceans (Perrin, 1975; Uhen, 2004; Chen et al., 2011). However, unlike most terrestrial mammals, the rostral elements of extant mysticete crania remain unfused throughout ontogeny; with the exception of the divisions of the occipital bone, sutures of the braincase in extant mysticetes are already at least partially ossified at birth (Walsh & Berta, 2011). Occipital ossification can be used to evaluate the relative age of OU 22163 and OU 22044. In the juvenile OU 22163, the basioccipital-basisphenoid synchondrosis is unfused and open but the basioccipital-exoccipital sutures are fused (Fig. 10). The occipital-parietal and frontal-parietal sutures are closed and corrugated; similarly, the squamosal-parietal suture is visible as an anastomosing suture line. Lastly, although the supraoccipital-exoccipital suture is not visible, persistent occipital fontanelles are present in the occipital shield (fontanelles are present in some extant adult odontocetes, such as *Mesoplodon* and phocoenids). Damage has obscured some of the holotype (OU 22044) sutures, but the basicranial fragment preserves a fused exoccipital-basioccipital suture; the occipital-parietal suture and frontal-parietal sutures are open, and the frontals have slipped anteriorly by about 2 cm (Fig. 3). Braincase sutures thus corroborate size-based identification of OU 22163 as a juvenile. Punctate texture on the occipital condyles of OU 22163 is further suggestive of juvenile status (Aguirre-Fernández & Fordyce, 2014). The occipital region of OU 22075 is not preserved. The nasofrontal suture is of possible use in *Waharoa*. In OU 22163 and 22044, the nasal bears a corrugated suture with the underlying frontal (Figs. 3 and 9), whereas in OU 22075 the nasals are slightly disarticulated and the suture is open (Fig. 8), identifying OU 22075 as an even younger juvenile. The median frontal suture is unfused in all three specimens, a unique feature amongst mysticetes; similarly the parietal–occipital and frontoparietal sutures are also unfused.

The symphyseal groove of cetaceans is a developmental vestige of the groove for the Meckel’s cartilage (Mead & Fordyce, 2009). In the mandible of the smallest specimen (OU 22075), the symphyseal groove extends far posteriorly and is continuous with a linear groove that can be traced along the entire length of the medial surface of the mandible (Fig. 20E). The symphyseal groove is present in adult aetiocetids and all adult Chaeomysticeti, but absent in mammalodontids and the Charleston toothed mysticetes (Fitzgerald, 2006; Fitzgerald, 2010; Fitzgerald, 2012); similarly, a groove is developed in juvenile odontocetes but lost during postnatal ontogeny (Mead & Fordyce, 2009). An extensive groove for the Meckel’s cartilage is absent in OU 22163, but the symphyseal groove is proportionally longer than in the holotype (OU 22044), indicating the gradual loss of this feature during postnatal ontogeny in *Waharoa*.

Vertebral epiphyseal fusion also has implications for ontogeny (Moran et al., 2015). All preserved vertebrae of the smaller specimens of *Waharoa* (OU 22075, 22163) have unfused vertebral epiphyses (Figs. 22–24), whereas the holotype (OU 22044) possesses fused vertebral epiphyses on the cervical and thoracic vertebrae (Figs. 21H–21M), suggesting maturity relative to OU 22075 and 22163. While not directly relating to epiphyseal fusion, the axis of OU 22075 bears a punctate texture on the atlantal articular surface as in extant juvenile cetaceans (Aguirre-Fernández & Fordyce, 2014). Other postcranial elements of OU 22075 (humerus) possess unfused epiphyses, further supporting identification as a juvenile.

Other aspects of the skeletal ontogeny of *Waharoa ruwhenua* are noteworthy. A sagittal crest is prominent in the adult (OU 22044), but absent in the large juvenile (OU 22163); a low but sharp sagittal crest is also present in the small juvenile. This suggests ontogenetic elaboration of the sagittal crest; similarly, the nuchal crests of the large juvenile (OU 22163) are relatively lower and less bladelike than in OU 22044. This probably corresponds to ontogenetic development of the temporalis musculature, and could reflect increase in size of the muscle in order to stabilize an increasingly more elongate beam-like mandible (see Functional implications of Rostromandibular Ontogeny). The atlas notably increases in anteroposterior length during postnatal ontogeny (Figs. 21 and 22); the atlas of adult eomysticetids is relatively massive and anteroposteriorly elongate but generally similar in proportion to that of basilosaurid archaeocetes. Modern mysticetes have anteroposteriorly flattened cervical vertebrae, and in some species some or all of the cervicals are fused (Balaenidae, *Caperea*, *Balaenoptera borealis* Lesson, 1828). The posterior cervicals are thinner than the atlas, but proportionally thicker than their counterparts in modern species. The anteroposteriorly thin atlas of the large juvenile of *Waharoa ruwhenua* implies that cervical thinning in later diverging mysticetes is a paedomorphic (rather than peramorphic) trait. This is surprising, as fusion of cervical vertebrae may be a peramorphic trait as it occurs late in *Balaena mysticetus* Linnaeus, 1758 postnatal ontogeny (Moran et al., 2015).

### Ontogenetic implications of osteohistology

Osteohistology presents a useful method towards inferring ontogenetic age in fossil vertebrates (Woodward, Padian & Lee, 2013). Many vertebrates preserve cyclically zoned bony tissues such as lines of arrested growth (LAGs) which can be used to accurately reconstruct the absolute ontogenetic age (in years) of fossil vertebrates (Woodward, Padian & Lee, 2013). Growth banding has been identified in tympanic bullae of extant mysticetes (Klevezal & Mitchell, 1971; Olsen, 2002) as well as mandibles (Olsen et al., 2003), although the accuracy of these skeletochronologic methods is problematic for mysticetes and less accurate than ear plugs (Olsen, 2002; Olsen et al., 2003). The possible growth bands in OU 22044 are too remodeled to permit convincing interpretation (Fig. 27C) Tissue organization and the extent of bone remodeling is a less accurate but useful method of determining relative (but not absolute) ontogenetic age (Woodward, Padian & Lee, 2013; Kerley, 1965). In general, the bones of rapidly growing juvenile mammals are characterized by disorganized woven bone, which can grade into more organized lamellar or remodeled bone during later growth. The rib of the smallest juvenile (OU 22075) is completely composed of woven bone with radially organized vascular channels (Fig. 27A). The holotype (OU 22044) retains some interstitial fragments of non-remodeled lamellar bone but the cortex is otherwise composed entirely of overlapping secondary osteons (Fig. 27C), indicating that the majority of the cortex is completely remodeled. The rib of the larger juvenile (OU 22163) is also composed mostly of disorganized woven bone, but there is a thin layer of laminar bone and sparse (non-overlapping) secondary osteons occur within the outer cortex (Fig. 27B). In terms of bone remodeling, osteohistology clearly identifies the largest specimen (OU 22044) as the most remodeled and therefore ontogenetically oldest, and the non-remodeled smallest specimen (OU 22075) as the ontogenetically youngest. Incipient bone remodeling in OU 22163 further indicates a slightly older ontogenetic age than OU 22075. Osteohistology thus supports the identification of OU 22075 as a young juvenile, OU 22163 as a somewhat older juvenile, and OU 22044 as an adult.

Cortical bone thickness (see Beatty & Dooley, 2009, for methods) is highest in the smaller juvenile (100%, OU 22075), lowest in the larger juvenile (47.4-55.55%, OU 22163) and intermediate in the adult (71.6%, OU 22044). The simplest interpretation of this pattern is that the medullary zone forms via resorption of vascular channels during early postnatal ontogeny, a process which has not yet started at the age at which OU 22075 died, but had occurred by age at which OU 22163 died. Slightly thicker cortex in the adult (OU 22044) may represent addition of lamellar bone during later ontogeny without a change in size of the medullary zone. Whether this pattern has any functional significance remains unclear. It must be stressed because incomplete rib fragments were sampled (although straight sections likely to be distal rib fragments), some of these differences in cortical bone thickness may represent variation between ribs or variation along the proximodistal axis (as in basilosaurids; De Buffrenil et al., 1990; Houssaye et al., 2015).

## Functional Morphology

### Rostral kinesis, palatal morphology, baleen, and dentition

Rostral articulations in the skull of *Waharoa ruwhenua* are intermediate between earlier diverging mysticetes (and archaeocetes) and extant mysticetes. Basilosaurids and most toothed mysticetes bear firmly closed or fused rostral sutures, indicating the rostrum was akinetic, while the rostral elements in all extant mysticetes are loosely articulated and open. In extant mysticetes the fronto-premaxillary and fronto-maxillary sutures are corrugated and closed (but not fused even in old age) and reduced to the rostral base. In *Waharoa ruwhenua*, maxillo-frontal contact was reduced to a small triangular facet lateral to the premaxilla. The maxilla-premaxilla suture is loose, but does bear some sculpturing (Fig. 9A), suggesting a largely open suture but perhaps slightly more rigid than extant mysticetes. The elongate nasals are set upon equally elongate anterior prongs of the frontal with which they share a corrugated articulation; in the ontogenetically oldest individual (OU 22044, holotype), the nasals have slipped anteriorly by 2 cm, indicating this suture had not yet fused. The premaxilla articulates with the dorsolateral surface of the nasal and medial part of the frontal, which bear a longitudinally corrugated articular surface. Anterior to the supraorbital process, the premaxilla would have dorsally overlapped the nasal, and the nasal articulated ventrally with the anterior prong of the frontal. The elongate corrugated nasofrontal and premaxilla-nasofrontal articulation indicates a somewhat more rigid rostrum in *Waharoa ruwhenua* than in extant mysticetes; nevertheless, this condition is substantially more flexible than that in toothed mysticetes, and represents the phylogenetically earliest occurrence of a kinetic rostrum among mysticetes.

The rostrum of *Waharoa ruwhenua* and all other eomysticetids is notable for its extreme length and narrow transverse width (Figs. 3, 7 and 8). Similar to balaenopterids, the rostrum of *Waharoa* is longitudinally flat (Fig. 5A and 5B) and not arched as in balaenids, *Caperea*, and *Eschrichtius*; the lateral margins of the palate are nearly parallel for most of their length as seen in dorsal view, unlike most extant mysticetes. Diagenetic compaction cannot be ruled out, but if arched, the rostrum of *Waharoa* would likely have only been gently arched as in *Balaenoptera*. The only mysticetes with flat or gently-arched rostra approaching the elongate proportions of eomysticetids are cetotheriids such as *Cetotherium*, *Piscobalaena*, and *Herpetocetus*. However, cetotheriids exhibit a strange craniomandibular joint differing from eomysticetids (Boessenecker, 2011; Gol’din, Startsev & Krakhmalnaya, 2014; El Adli, Deméré & Boessenecker, 2014) and an elongate, narrow rostrum may not reflect convergent feeding behavior.

The palatal morphology of *Waharoa ruwhenua* is also intermediate between toothed and extant mysticetes. *Waharoa ruwhenua* has a vascularized palate unlike most toothed mysticetes, and bears considerably more and larger palatal foramina and sulci than *Aetiocetus* spp. and *Chonecetus goedertorum* Barnes & Furusawa, 1995, in Barnes et al., 1995 (e.g., Deméré et al., 2008); likewise, *Waharoa ruwhenua*, and other eomysticetids, differ from extant mysticetes in possessing a shallowly convex rather than deep and sharp longitudinal keel (unknown in OU 22044). *Waharoa ruwhenua* differs from all extant mysticetes in having maxillae with lateral palatal foramina and sulci (osteological correlates of baleen; Deméré et al., 2008) present only on the posterior two-thirds of the palate. The anteriormost foramina on the palate of *Waharoa* are a bilateral pair of larger foramina with deeply entrenched anteriorly directed sulci (OU 22163, 22075); they are positioned medially and thus are not contiguous with the lateral palatal foramina. Similar foramina exist in *Janjucetus* and *Aetiocetus* (Fitzgerald, 2006; Deméré & Berta, 2008). Studies of extant *Eschrichtius* palatal structures indicate that these are the greater palatine foramina (Ekdale, Deméré & Berta, 2015) and thus in *Waharoa*, only the lateral palatal foramina are functionally related to the presence of baleen. Absence of well-developed of osteological correlates of baleen in the anterior 1/3 of the palate bears implications for feeding ecology (see below).

**Figure 29:**
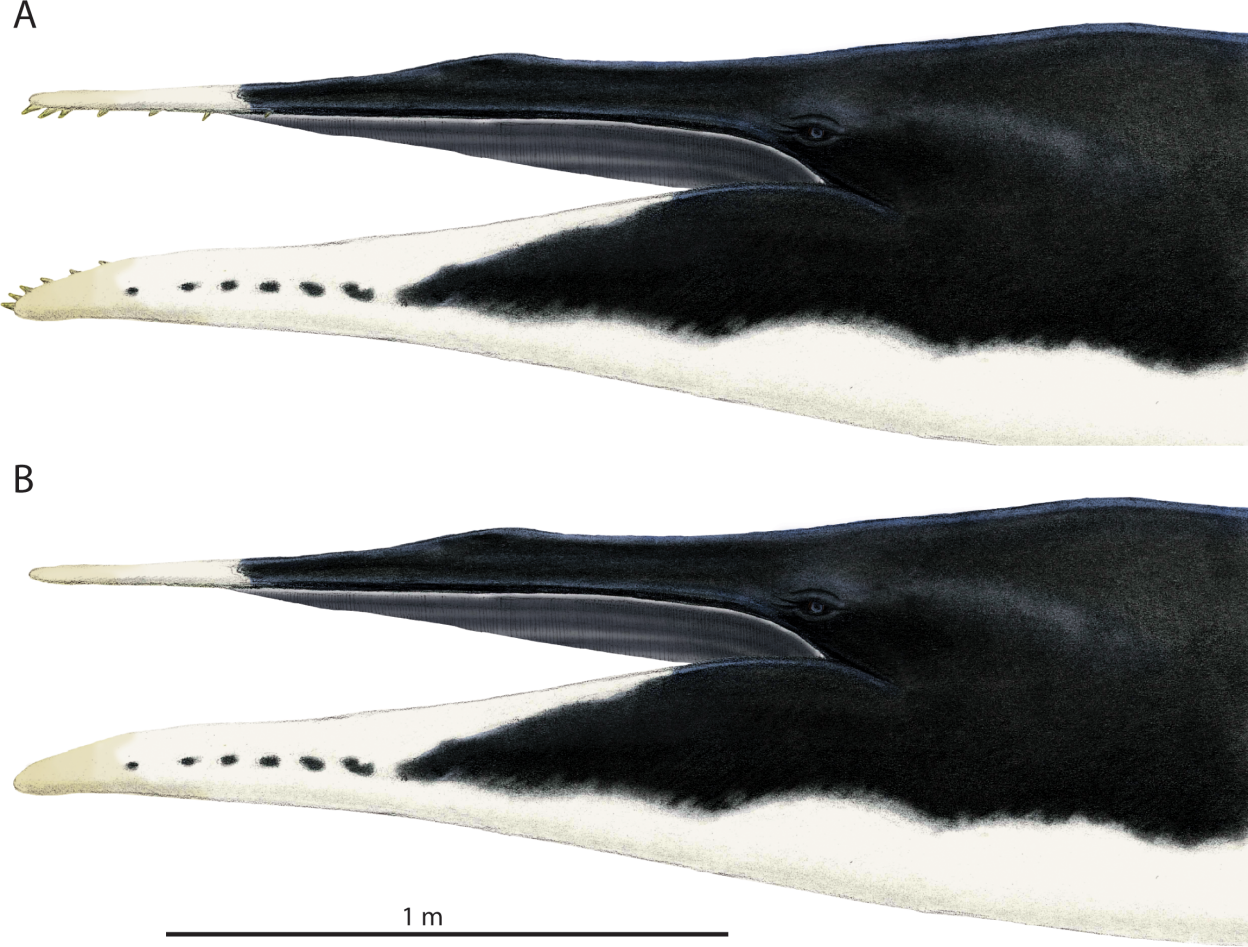
Alternative life restorations of *Waharoa ruwhenua*. (A) with erupted permanent dentition; (B) without dentition.

The premaxilla, maxilla, and mandible of *Waharoa ruwhenua* differ from all extant postnatal mysticetes in preserving well-developed (possibly vestigial) alveoli: 6–13 upper alveoli and at least 3 mandibular alveoli (Fig. 6). The premaxilla bears three circular-oval alveoli, corresponding to the primitive number of teeth present in toothed mysticete premaxillae (Barnes et al., 1995; Fitzgerald, 2006; Deméré & Berta, 2008). Several dorsoventrally shallow alveoli (at least three, possibly up to ten) are present along the anterior alveolar margin of the maxilla. Three alveoli are also present dorsally at the anterior tip of the mandible; posteriorly the morphology of the alveolar groove is incompletely prepared. Likewise, alveoli were reported in *Yamatocetus canaliculatus* by Okazaki (2012), who interpreted these structures as housing adult mineralized teeth despite the lack of preserved teeth. All three carefully prepared skulls of *Waharoa ruwhenua* lack associated teeth, but a single putative tooth was recovered from matrix associated with the palate of the eomysticetid *Tokarahia* sp., cf. *T. lophocephalus* (OU 22081; Boessenecker & Fordyce, in press). The possible presence of adult teeth in *Tokarahia* suggests that the alveoli of *Waharoa* (and *Yamatocetus*) did in fact house adult teeth. Further evidence suggesting adult dentition in *Waharoa* and *Yamatocetus* includes the anterior thickening of the lateral edge of the maxilla; the posterior edge of the maxilla is very thin and easily damaged in all known eomysticetids (except where intact in *Yamatocetus*). Anterior thickening of the maxilla may have afforded sufficient structural support for a functional dentition. All alveoli are circular-oval or dorsoventrally flattened, small, and anteriorly inclined (mandibular, premaxillary alveoli) or laterally directed (maxillary alveoli), and are only present along the anterior third of the rostrum where osteological correlates of baleen (lateral foramina and sulci) happen to be absent. If such a rudimentary dentition were present, it would have served an uncertain role in feeding (see below). Notably, there is no ontogenetic decrease in the size of the alveoli relative to the dimensions of the premaxilla (Figs. 6A and 6B). It is unclear whether adult teeth were present (as in *Tokarahia*) and erupted in *Waharoa ruwhenua* (Fig. 29A), present but unerupted, or absent (Fig. 29B), and discovery of more complete material is required to further address this issue.

### Ontogeny of the auditory apparatus

Cetaceans possess adaptations in the basicranium, tympanoperiotic complex, and mandible for directional hearing underwater (Nummela et al., 2007). Included within these modifications is an increase in the size, volume, and density of the tympanoperiotic complex (De Buffrenil, Dabin & Zylberberg, 2004; Nummela et al., 2007; Cozzi et al., 2012; Lancaster et al., 2015). Modern cetaceans have tympanoperiotic complexes that are nearly completely ossified at birth and grow considerably slower during postnatal ontogeny (Oishi & Hasegawa, 1995; De Buffrenil, Dabin & Zylberberg, 2004; Cozzi et al., 2012; Lancaster et al., 2015). Although the stepwise accumulation of auditory adaptations for aquatic existence is well known through study of archaeocete basicrania (Nummela et al., 2007), it is unclear when precocial development of enlarged, densely mineralized tympanoperiotics evolved amongst cetaceans.

**Figure 30:**
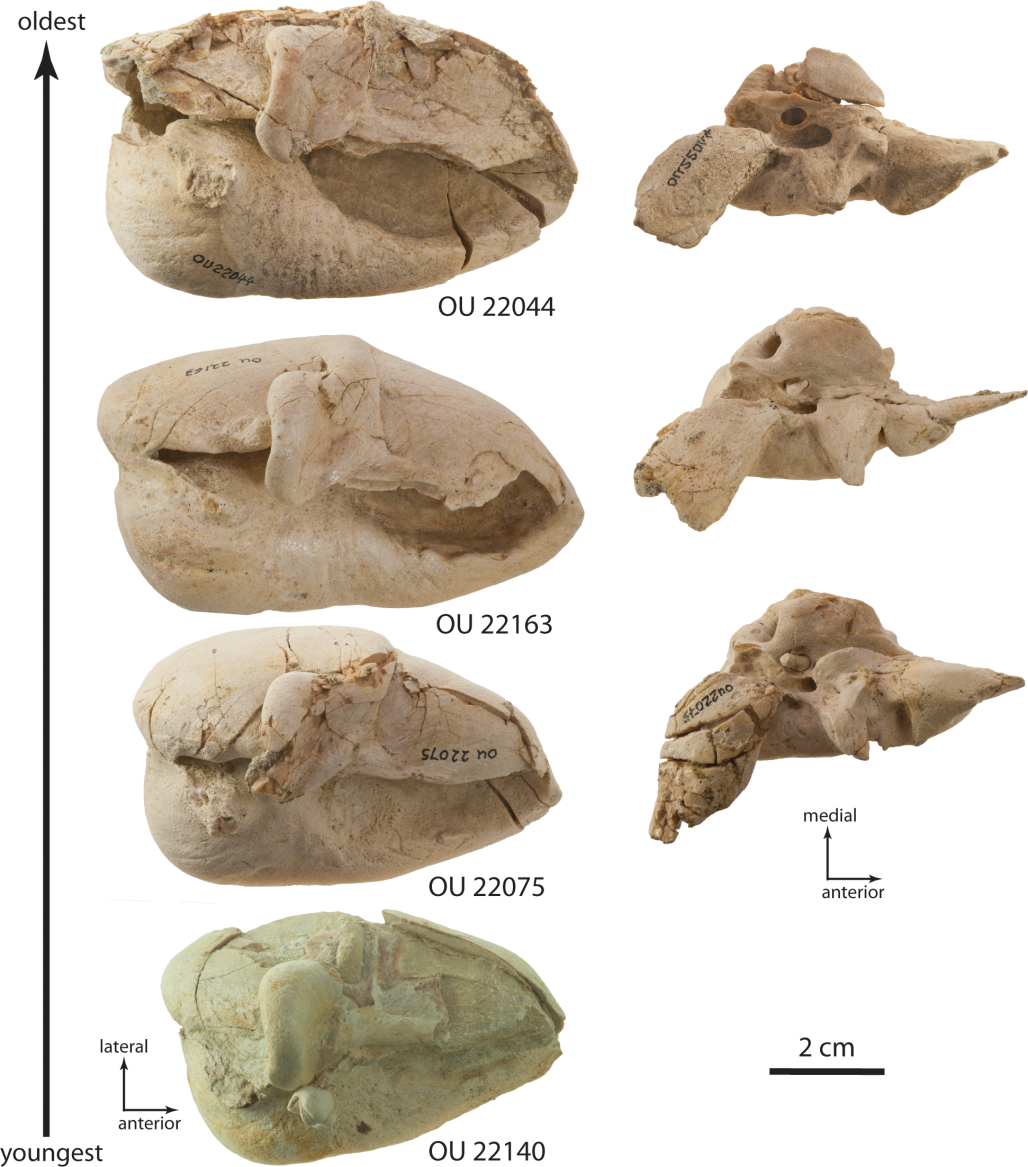
Tympanoperiotic ontogeny of *Waharoa ruwhenua*.

The ontogenetic series for *Waharoa ruwhenua* includes well-preserved tympanic bullae and periotics (Figs. 11–13, 16–19 and 29) for two juveniles (OU 22075, 22163) and an adult (OU 22044). The juvenile periotics are nearly identical to the adult in dimensions and morphology, and differ only in the morphology of the ventral opening of the facial canal, the morphology of the posteroexternal foramen, and the histology of the suprameatal region. The visible change from surficial woven cancellous bone in the suprameatal fossa (OU 22075, 22163) to smooth cortical bone (OU 22044) in *Waharoa* reflects the last stages of ossification of the periotic. The widening and anterior extension of the ventral opening of the facial canal (Fig. 30) is less easily interpreted, but correlates well with the postnatal elongation of the rostrum in *Waharoa*. The facial canal transmits the facial nerve, which innervates superficial muscles of the head, posterior belly of the digastric, stapedius, stylohyoideus, platysma, and taste buds of the palate and anterior two-thirds of the tongue (Evans & de Lahunta, 2013); the facial nerve may increase in size during ontogeny to accommodate the rapidly growing feeding apparatus. Less certain is the significance of the ontogenetic change of the posteroexternal foramen from a small circular pore into an elongate fissure. These subtle ontogenetic changes in the periotic contrast markedly with the rearrangement of endocranial foramina of the periotic and extreme elongation of the compound posterior process in *Balaenoptera* (Bisconti, 2001); similar changes in endocranial foramina are known in *Herpetocetus bramblei* Whitmore & Barnes, 2008 (Boessenecker & Geisler, 2008).

Like the periotic, juvenile specimens of *Waharoa ruwhenua* possess near-adult size tympanic bullae (Fig. 30). However, unlike the periotics, the tympanic bullae of OU 22075 and OU 22163 are slightly smaller than the holotype adult. Because OU 22075 and 22163 are relatively small and exhibit osteohistology indicative of juvenile status relative to OU 22044, this gradual size increase in the tympanic bulla may be identified as an ontogenetic trend. The tympanic bullae of *Waharoa ruwhenua* were relatively large during early postnatal ontogeny (79–86% of adult size in OU 22140 and 22075, respectively), but still grew slightly larger after birth. In comparison, extant odontocete tympanoperiotics are adult-sized at birth and do not appear to increase in size (Kasuya, 1973; De Buffrenil, Dabin & Zylberberg, 2004: Fig. 2A); extant *Balaenoptera acutorostrata* Lacepede and (Lacépède, 1804) tympanic bullae follow a similar pattern (Oishi & Hasegawa, 1995: Fig. 2). Near-adult size of the tympanic bulla and adult size of the periotic during early postnatal ontogeny indicate precocial development of the auditory apparatus in *Waharoa ruwhenua*, and the earliest occurrence of this innovation amongst fossil cetaceans. Because *Waharoa ruwhenua* is an early neocete and extant odontocetes and mysticetes share this trait (e.g., Oishi & Hasegawa, 1995: Fig. 2; De Buffrenil, Dabin & Zylberberg, 2004: Fig. 2), early ossification of the tympanoperiotic is probably a common feature of Neoceti. Published figures and measurements of the basilosaurid *Dorudon atrox* also indicate that juveniles possessed relatively large tympanic bullae (Uhen, 2004: Fig. 27; appendix IVB), perhaps indicating that precocial development of enlarged, dense tympanoperiotics is apomorphic even at the level of the clade Pelagiceti.

**Figure 31:**
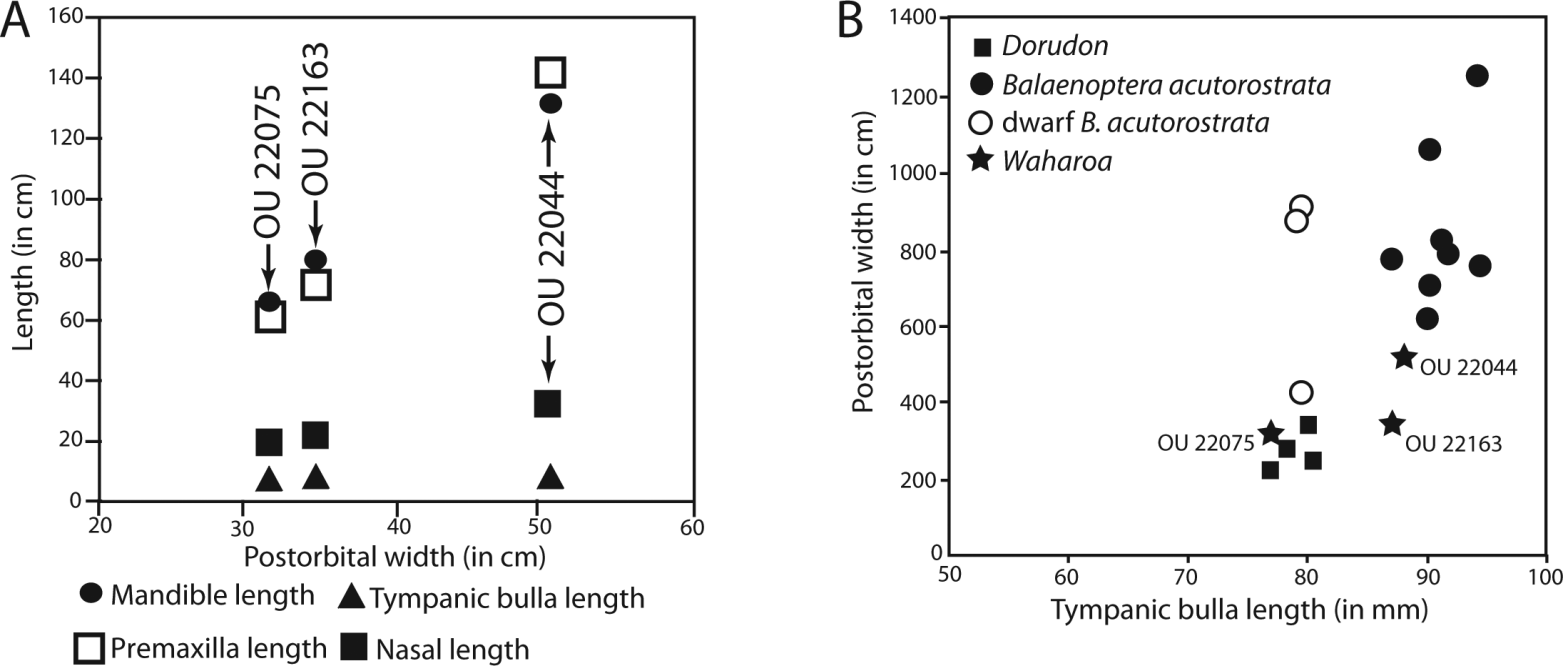
Ontogenetic change in rostral, mandibular, and tympanic bulla length in *Waharoa ruwhenua*. (A) Relative length of premaxilla, nasal, mandible, and tympanic bulla as compared to postorbital width. (B) Length of tympanic bulla relative to postorbital width of *Dorudon atrox* (measurements from Uhen (2004)), *Balaenoptera acutorostrata* and *B. bonaerensis* (Omura, 1957; Omura, 1975; and specimens reported by Watson & Fordyce, 1993), dwarf *Balaenoptera acutorostrata* (Arnold, Marsh & Heinsohn, 1987; Paterson et al., 2000; Secchi et al., 2003), and *Waharoa ruwhenua*. Bizygomatic width measurements from literature used instead of postorbital width for *Balaenoptera acutorostrata* to maximize available samples.

The juvenile tympanic bulla are relatively large and therefore likely functionally mature. The slight increase in size from 68 to 84 mm from juvenile to adult suggests a wider variation in tympanic bulla size for stem mysticetes than recorded by Oishi & Hasegawa (1995, for *Balaenoptera acutorostrata*) who reasonably indicated bulla length as a useful criterion for identification of isolated mysticete bullae. Measurements of bulla length relative to skull width (Fig. 31B) show a wider range in bulla size than extant minke whales. The variation in size amongst *Waharoa ruwhenua* suggests that isolated stem mysticete bullae of near identical morphology but differing in length by up to 2 cm may well be referable to the same species. Additional studies of extant mysticete auditory ontogeny and tympanoperiotic variation, particularly focusing on gray whales (*Eschrichtius*) and right whales (Balaenidae) are necessary to more precisely evaluate methods of isolated tympanoperiotic identification for Neogene mysticetes.

### Craniomandibular morphology and feeding

*Waharoa ruwhenua* shares a similarly rectilinear and flat rostrum with extant balaenopterids, but appears to have lacked several adaptations for lunge feeding present in modern rorquals. The squamosal of *Waharoa ruwhenua* has a concave glenoid fossa (Figs. 4, 9 and 10) with distinct margins; obviously concave glenoid fossae are present in other Eomysticetidae for which the squamosal is well-preserved (*Eomysticetus carolinensis* Sanders & Barnes, 2002, *Eomysticetus whitmorei*, *Micromysticetus rothauseni*, *Tohoraata raekohao*, *Tokarahia lophocephalus*, and *Yamatocetus canaliculatus*; also present in *Cetotheriopsis lintianus* Meyer, 1849), in addition to Aetiocetidae, Mammalodontidae, *Horopeta*, archaeocetes, and extant Balaenidae (right whales; *Balaena mysticetus*, *Eubalaena* spp.). However, in extant gray whales (*Eschrichtius robustus*) and rorquals (*Balaenoptera*, *Megaptera*) and nearly all fossil mysticetes included within the “Thalassotherii” of Bisconti, Lambert & Bosselaers (2013), the glenoid fossa is indistinct and flat or convex and inflated. Extant balaenids are known to primitively retain a synovial craniomandibular joint (Eschricht & Reinhardt, 1866; Van Beneden, 1882; Lambertsen, Ulrich & Straley, 1995; Lambertsen et al., 2005), while extant balaenopterids instead possess a fibrocartilaginous craniomandibular joint pad (Beauregard, 1882; Schulte, 1916; Lambertsen, Ulrich & Straley, 1995); a similar fibrocartilaginous joint with a vestigial synovial capsule is present in *Eschrichtius* (Johnston et al., 2010; El Adli & Deméré, 2015). The fibrocartilaginous joint pad permits extreme movement of the mandible relative to the limited movement allowed by a synovial joint, including dislocation of the craniomandibular joint and lateral displacement of the posterior mandible, longitudinal twisting of the mandible, and opening of the mouth to over 90° during lunge feeding (Lambertsen, Ulrich & Straley, 1995). Owing to the phylogenetic distribution of synovial craniomandibular joints and concave glenoid fossae in extant mysticetes, a distinct glenoid fossa is here proposed as a bony correlate of a synovial joint capsule for the craniomandibular joint. The converse, however, may not be universal as juvenile *Caperea marginata* possesses an indistinctly concave glenoid fossa and a synovial craniomandibular joint (RE Fordyce, 2011, unpublished data). Because *Waharoa ruwhenua* and most other eomysticetids possess this feature, a synovial joint capsule was likely primitively present in these extinct mysticetes in addition to all toothed mysticetes. An anteroposteriorly elongate coronoid process lacking lateral deflection of the apex also suggests a simple hinge without the rotation seen in extant balaenopterids.

The probable absence of a fibrocartilaginous craniomandibular joint in *Waharoa ruwhenua* suggests that it was incapable of complex mandibular rotation and dislocation relating to maximizing oral volume. Furthermore, the posterior end of the mandible of *Waharoa* is delicate and includes a small, anteroposteriorly thin mandibular condyle and a cavernous mandibular foramen and transversely thin (∼1 mm thick) “pan bone” like Odontoceti and basilosaurid archaeocetes (Figs. 3, 4 and 20). As in other eomysticetids the coronoid process is dorsally directed and not laterally deflected as seen in Balaenopteridae, some Cetotheriidae, and “cetotheres” *sensu lato*, which further suggests that the mandibles of *Waharoa* did not rotate longitudinally as in modern rorquals (Lambertsen, Ulrich & Straley, 1995). In contrast, the posterior mandible of extant balaenopterids is robust with a small mandibular foramen and a large, thick and subspherical mandibular condyle. The delicate construction of the posterior mandible suggests that *Waharoa ruwhenua* and other eomysticetids were incapable of lunge feeding, as it would likely fracture during violent abduction of the mandibles. Lastly, the mandibles of *Waharoa ruwhenua* and other eomysticetids are only slightly laterally bowed in comparison to extant Balaenopteridae. The strongly bowed mandibles of extant balaenopterids rotate longitudinally during mandibular abduction, facilitating the capture of a larger cross-section than otherwise available from the dimensions of the palate alone (Lambertsen, Ulrich & Straley, 1995). In contrast, the mandibles of *Waharoa* are only incipiently bowed (Figs. 3, 4 and 20), indicating that it had not evolved towards a similar optimum of maximized oral volume and may not have rotated its mandibles during feeding.

### Functional implications of Rostromandibular Ontogeny

**Figure 32:**
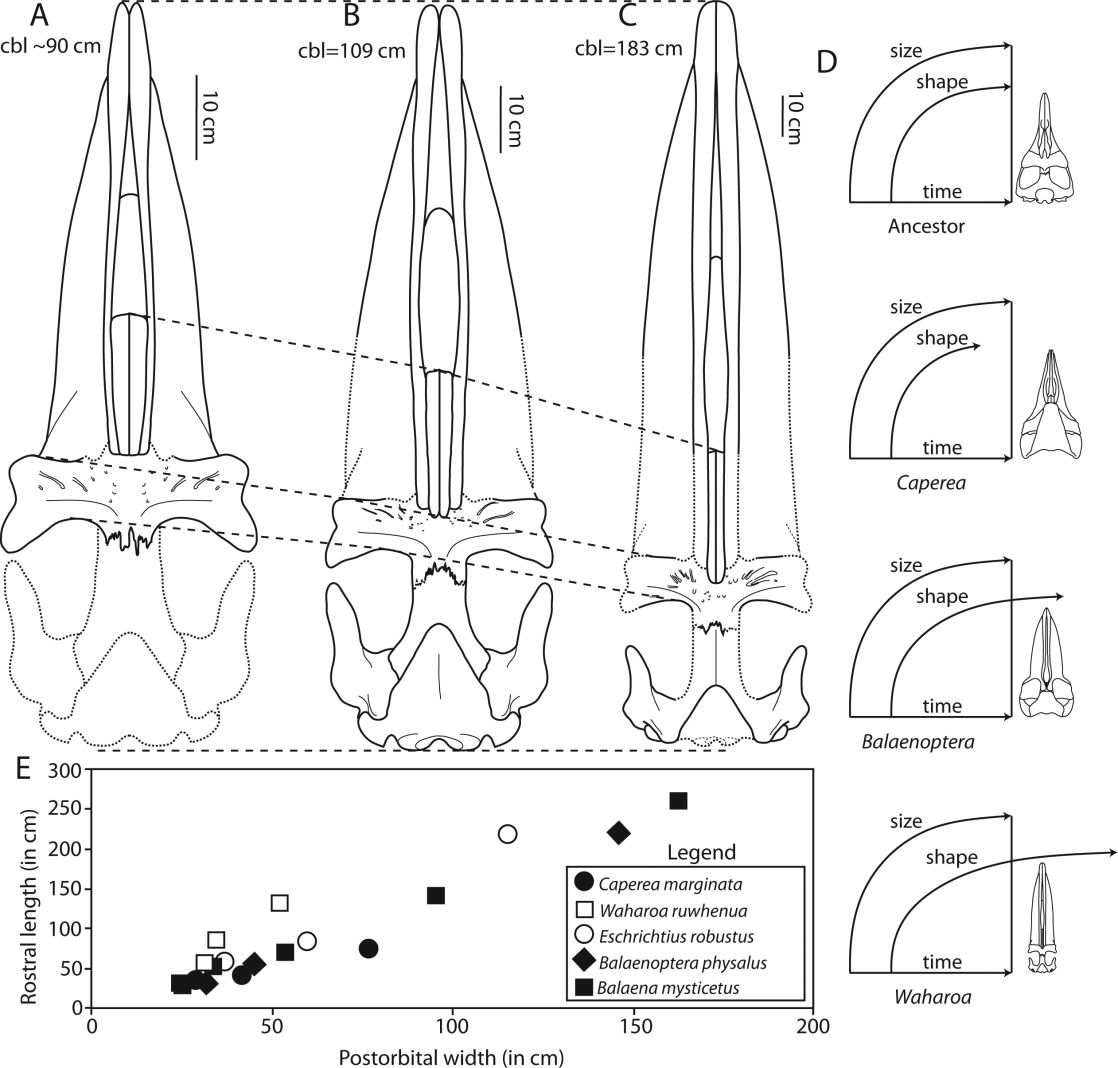
Rostral ontogeny in *Waharoa ruwhenua*. Comparison of relative proportion of temporal fossa, cranium length, nasal length, and condylobasal length in (A) small juvenile (OU 22075); (B) large juvenile (OU 22163), and (C) adult (OU 22044). (D) ontogenetic clock models of hypothetical archaeocete ancestor, *Caperea, Balaenoptera, and Waharoa*, modified from Tsai & Fordyce (2014a). (E) Relation between rostrum length and postorbital width in *Balaena, Balaenoptera, Caperea, Eschrichtius*, and *Waharoa*.

The well-preserved ontogenetic series of *Waharoa ruwhenua* described herein clearly demonstrates that the rostrum and mandibles of this extinct mysticete elongated rapidly during postnatal ontogeny with minimal widening (Figs. 32A–32C and 32E). This postnatal increase in rostrum length indicates that the feeding strategy of *Waharoa ruwhenua* was optimized towards a longer palate than most extant mysticetes. Furthermore, the rostrum of *Waharoa ruwhenua* appears to become anteroposteriorly elongated during postnatal ontogeny at a more rapid rate than extant balaenopterids and balaenids (Figs. 32D–32E), the former of which are characterized by the peramorphic process of acceleration (Tsai & Fordyce, 2014a). Ontogenetic evidence thus suggests that the elongate rostrum was positively selected amongst the Eomysticetidae, rather than merely a vestige of the elongate rostrum of archaeocetes. Additionally, all earlier diverging mysticetes are characterized by relatively short rostra, further indicating that the longirostrine nature of eomysticetid crania is derived. The more extreme rate at which the rostrum elongates relative to skull width during postnatal ontogeny in comparison to other mysticetes such balaenopterids (Fig. 32E) suggests that the peramorphic process of acceleration has caused the longirostrine morphology of *Waharoa* and other eomysticetids, and that this process is exaggerated in eomysticetids relative to balaenopterids (Fig. 32D).

### Feeding ecology of *Waharoa ruwhenua* and implications for early Chaeomysticete evolution

The peculiar feeding apparatus of *Waharoa ruwhenua* and other Eomysticetidae may be interpreted in the context of feeding behavior and functional anatomy of extant baleen whales. Extant mysticetes filter feed using three different behaviors (Werth, 2000): lunge (or engulfment) feeding (Balaenopteridae), benthic suction feeding (Eschrichtiidae), and skim (or ram) feeding (Balaenidae). Lunge-feeding rorquals swim rapidly towards prey and engulfs a large volume of prey-laden water by abducting its mandibles. During jaw opening, the bowed mandibles dislocate at the craniomandibular joint and abduct over a 90° angle and rotate longitudinally, increasing the cross-sectional area of the oral cavity. As the mandibles are abducted, the prey-laden mass of water passively inflates the oral cavity via the distensible ventral throat pouch (Pivorunas, 1979; Lambertsen, 1983; Lambertsen, Ulrich & Straley, 1995) and high drag slows the whale to a standstill (Goldbogen, Pyenson & Shadwick, 2007). As the mandibles are adducted and rotate back into “occlusion” with the maxillary margin, water is expelled through the baleen as the throat pouch is tightened and collapsed (Pivorunas, 1979; Lambertsen, 1983; Werth, 2000). As discussed above, *Waharoa* lacks the skeletal adaptations for lunge feeding as seen in extant balaenopterids (e.g., strongly bowed mandibles, laterally deflected coronoid process, robust posterior mandible and reduced mandibular foramen, and probable fibrocartilaginous craniomandibular joint), and was probably incapable of lunge feeding. Other studies have identified posteriorly extended and “telescoped” rostral elements, rostral kinesis, and anteriorly thrusted occipital shield of balaenopterids (and some cetotheriids) as adaptations for specialized lunge feeding (Bouetel, 2005; Godfrey, Geisler & Fitzgerald, 2013); in contrast, the skull of *Waharoa* exhibits weakly “telescoped” posterior rostral elements, rigid medial rostral elements, and limited thrusting of the occipital shield, consistent with the lack of lunge feeding adaptations in the Eomysticetidae.

The gray whale (*Eschrichtius robustus*) feeds by rolling onto its side and ingesting large volumes of prey-laden sediment into the oral cavity, and filtering out sediment and water (Nerini, 1984; Werth, 2000). The feeding apparatus of *Eschrichtius robustus* is intermediate in some regards between skim feeding balaenids and lunge feeding balaenopterids. Balaenid-like features include a narrow and dorsally arched rostrum, a possible subrostral gap in the baleen at the rostral terminus, a mandible with limited lateral bowing, and a reduced coronoid process on the mandible; balaenopterid-like features include a fibrocartilaginous craniomandibular joint and strongly “telescoped” rostral elements. Despite reports of suction feeding in extant gray whales, osteological features specifically pertaining to suction feeding are unremarkable (Johnston & Berta, 2011). This may be unsurprising given that gray whales are capable of lunge feeding like rorquals (Nerini, 1984) and are perhaps better characterized as generalists (Johnston & Berta, 2011). Because no distinctive osteological correlates of benthic suction feeding have been identified in extant *Eschrichtius* (Johnston & Berta, 2011), it is not possible to evaluate benthic suction feeding in *Waharoa ruwhenua*. However, it is worth noting that, as for lunge feeding, the delicate posterior of the mandible may have been too fragile to cope with stresses involved in scooping large volumes of sediment. Although *Eschrichtius* lacks obvious adaptations for benthic feeding, at least two genera of chaeomysticetes in the Cetotheriidae (*Cetotherium* and *Herpetocetus*) have been proposed as benthic suction feeders owing to a unique craniomandibular articulation that restricted abduction of the mandible but emphasized longitudinal rotation (Gol’din, Startsev & Krakhmalnaya, 2014; El Adli, Deméré & Boessenecker, 2014), but these features adaptations are lacking in *Waharoa* and other eomysticetids.

Extant right whales (*Balaena* and *Eubalaena*) employ skim (or ram) feeding behavior (Pivorunas, 1979; Werth, 2000; Werth, 2004; Lambertsen et al., 2005). Right whales swim slowly through the water column, and water enters the oral cavity via the large subrostral gap. Flow is primarily laminar and unidirectional, and water additionally flows through the orolabial sulcus between the baleen and the dorsally arched lower lip; negative oral pressure via the Bernoulli and venture effects is generated by water flowing at a higher through the orolabial sulcus rather than medial to the baleen, preventing the formation of a compressive bow wave (Werth, 2004; Lambertsen et al., 2005). The efficiency of this style of skim feeding is directly related to the cross-sectional area of the filter feeding apparatus (Lambertsen et al., 2005); in balaenids, the cross-sectional area of the filter feeding apparatus is maximized by rostral arching elongation of baleen plates (up to 4 m in length in *Balaena*; Werth, 2004). Because the range of mandibular abduction and magnitude of stresses imposed upon the craniomandibular joint during skim feeding are minimal in comparison to lunge feeding, dislocation of the craniomandibular joint does not occur during skim feeding and right whales primitively retain a synovial (rather than fibrocartilaginous) craniomandibular joint (Lambertsen, Ulrich & Straley, 1995). The enlarged, elongate temporal fossa of *Waharoa* and other eomysticetids appears, based on the small fossa of most toothed mysticetes, secondarily derived in eomysticetids. This, in concert with a large coronoid process and enormous temporal fossa indicates the temporalis muscle—responsible for adducting the mandible—was relatively large and important during feeding. Such large jaw-closing muscles could indicate lunge feeding behavior if not for the delicate mandible lacking a laterally deflected coronoid process and the probable retention of a synovial craniomandibular joint. Instead, an enlarged temporalis could have been important to stabilize the beam-like mandible at the optimal angle during skim feeding, as the position of the lower lip relative to the baleen and orolabial sulcus is critical to maintaining unidirectional flow through the oral cavity and baleen (Werth, 2004; Lambertsen et al., 2005).

*Waharoa* lacks certain adaptations consistent with lunge and benthic suction feeding and further possessing certain skeletal features which may have precluded these behaviors, the peculiar feeding apparatus of *Waharoa ruwhenua* and other eomysticetids is consistent with a style of skim feeding analogous to extant right whales. Like balaenids, *Waharoa* may have possessed a large subrostral gap, although formed by the possible absence of baleen in the anteriormost rostrum rather than laterally splayed baleen racks as in balaenids. The degree to which baleen was absent is unclear, and we speculate that to be functionally viable perhaps baleen was continually present along the lateral margin. The inferred retention of a synovial craniomandibular joint and delicate mandible likely precludes lunge feeding, but is consistent with skim feeding; elongate temporal fossa and coronoid process and associated massive temporalis musculature suggests an important role in the stabilization and closure of the beam-like mandible. The most obvious difference with balaenids is the lack of rostral arching in *Waharoa ruwhenua*, a pathway towards maximizing the cross-section of the feeding apparatus. In *Waharoa ruwhenua*, the flat rostrum elongates rapidly during postnatal ontogeny. A short rostrum is primitive amongst mammals and optimized for suckling in neonatal cetaceans, but the rate at which the rostrum of *Waharoa ruwhenua* grows during postnatal ontogeny is more extreme than any other group of mysticetes (Fig. 32D). Palatal elongation represents an incipient pathway for maximizing the size of the filter feeding apparatus in lieu of rostral arching; perhaps eomysticetids were incapable of growing long baleen plates. As *Waharoa* lacks an arched rostrum and (by inference) elongate baleen plates, the filter feeding apparatus had a proportionally smaller cross section than in extant balaenids. Because all other described eomysticetids do not differ substantially from *Waharoa* (with one exception: palatal foramina extending slightly further anteriorly in *Yamatocetus canaliculatus*; (Okazaki, 2012): Figs. 3 and 4), a similar feeding behavior seems reasonable for the entire family. Isotopic analyses of New Zealand Eomysticetidae appear to corroborate these functional interpretations. The tympanic bulla of *Tokarahia* sp., cf. *T. lophocephalus* (OU 22081) was sampled for *δ*^13^C and yielded some of the lowest values yet reported for any mysticete, modern or extinct; these low values are similar to those of modern Balaenidae, and suggest a similar diet of zooplankton (Clementz et al., 2014). Differences with adult balaenids of uncertain functional significance include the presence of a large coronoid process and an anteroposteriorly elongate temporal fossa; an anteroposteriorly elongate coronoid process is present in juvenile *Eubalaena* (Marx et al., 2013). However, the temporal fossa of balaenids is similarly large (in comparison to other crown Mysticeti) but transversely wide and anteroposteriorly shortened rather than anteroposteriorly elongate, possibly suggesting analogous importance in stabilizing the mandible during feeding. Finite element modeling of the craniomandibular joint in eomysticetids and other modern and fossil mysticetes could elucidate capability for lunge feeding, and test hypotheses presented above.

In both a geochronologic and phylogenetic context, eomysticetids like *Waharoa ruwhenua* are the earliest obligate filter feeders amongst the Mysticeti. Some hypotheses on the evolution of filter feeding have been advanced based upon the feeding apparatus of toothed mysticetes (Fordyce, 1984; Fitzgerald, 2006; Fitzgerald, 2010). Because eomysticetids lack a functional dentition, their feeding behavior is more directly relevant towards understanding the early adaptive history of filter feeding in chaeomysticetes than any other group of baleen whales. Some eomysticetids such as *Waharoa* and *Yamatocetus* possess apparent alveoli and may have housed adult teeth (Okazaki, 2012; Fig. 28), and *Tokarahia* preserves a putative isolated peglike tooth (Boessenecker & Fordyce, in press); the presumed dentition of eomysticetids was rudimentary and likely restricted to the anterior third of the feeding apparatus. In *Waharoa*, the alveoli are shallow and separated by wide diastemata, suggesting a few (6–13 in the rostrum) peg-like, shallowly rooted teeth (Fig. 28A) like the putative tooth of *Tokarahia*. Such a dentition would not function well as an adaptation towards filter feeding. It is unclear whether or not an adult dentition was present in *Waharoa*, and if present, whether it was truly vestigial or retained for social purposes (e.g., display or antagonistic behavior, as in Ziphiidae).

Numerous phylogenetic studies have recovered balaenids and neobalaenids as the earliest diverging lineage of crown Mysticeti, and notably balaenids (or balaenoids) frequently constitute the next diverging lineage crownward of the *Eomysticetidae* (Geisler & Sanders, 2003; Marx, 2011). The interpretation of *Waharoa ruwhenua* and other eomysticetids as skim feeding specialists and the presence of skim-feeding balaenids as the next diverging lineage of mysticetes suggests that skim feeding may reflect the primitive mode of feeding amongst the Chaeomysticeti (although see Tsai & Fordyce, 2015). The oldest known published balaenid, *Morenocetus parvus* Cabrera, 1926, dates to the early Miocene (Cozzuol, 1996; Burdigalian according to Cione et al., 2011), and right whale-like chaeomysticetes with arched rostra, tentatively identified as early right whales, have been reported from the same upper Oligocene strata in New Zealand that produced *Waharoa ruwhenua* Fordyce, 2002. Competition with newly evolved right whales and right whale-like mysticetes may have driven eomysticetids to extinction near the Oligo-Miocene boundary.

### Breeding

The presence of relatively young juvenile specimens of *Waharoa ruwhenua*, likely under one year old, indicates that the continental shelf of Zealandia was perhaps a calving or nursing ground during the Oligocene. No substantial areas of continental shelf exist between Zealandia and the equator, and because modern mysticetes tend to calve in continental waters along lowermost latitudes of their geographic ranges (Hindell, 2009), the continental shelf of Zealandia was the area of continental shelf nearest the equator. Although specimens of *Waharoa* were not sampled, *δ*^13^ C values of other eomysticetids (*Tokarahia* sp., cf. *T. lophocephalus*, OU 22081) have relatively low *δ*^13^ C values consistent with latitudinal migration (Clementz et al., 2014). The eastern North Pacific gray whale calves during the winter months in subtropical lagoons in Baja California (20°N), and embarks on a long distance migration to the Bering Sea (<75°N), totaling over 55°of latitude (Jones & Swartz, 2009). Analogous seasonal migrations to productive Antarctic waters and a winter return to breed along the Zealandia shelf may have been possible for *Waharoa ruwhenua*; additional isotopic study could possibly shed further elucidate the breeding behavior and life history of stem mysticetes.

## Conclusions

The new eomysticetid *Waharoa ruwhenua* is the first stem mysticete, and the first early neocete, for which an ontogenetic series of fossils is available for study. Amongst eomysticetids, *Waharoa* has a gracile skull, anteriorly oriented zygomatic processes, small periotics with a short anteroposteriorly directed and smooth posterior bullar facet, narrow and dorsoventrally shallow tympanic bullae, transversely wide atlases and axes and posterior cervicals with dorsoventrally deep vertebral foramina. External morphology, suture development, and osteohistology clearly identifies the smallest specimen (OU 22075) as a young juvenile, a slightly larger specimen as mature juvenile (OU 22163), and the holotype as an old adult. Several craniomandibular changes are noted through ontogeny, including anteroposterior lengthening of the rostrum, nasals, and mandibles, decrease in the size of the symphyseal groove, elaboration of the sagittal and nuchal crests, lengthening of the tympanic bulla, and increase in diameter of the facial canal of the periotic. Postnatal growth of the feeding apparatus is more extreme even than within modern rorquals, indicating that the long rostrum of eomysticetids is not simply a primitively inherited condition but that an elongate feeding apparatus was positively selected for. Distinct glenoid fossae indicate the presence of synovial craniomandibular joints, and in concert with the delicate posterior mandible indicate that *Waharoa* was likely not capable of rapid lunge feeding like rorquals. The lack of lateral palatal sulci from the anterior third of the palate may suggest the absence of baleen from the rostral terminus, perhaps forming a subrostral gap functionally analogous to that of balaenids and permitting skim feeding behavior. Osteohistology indicates that *Waharoa* primitively retained dense osteosclerotic ribs but lost localized pachyosteosclerosis characteristic of archaeocetes. Discovery of several juvenile *Waharoa* from New Zealand suggests that the continental shelf of Zealandia served as a calving ground for some of the earliest toothless mysticetes, perhaps serving as a warm-water winter habitat prior to a latitudinal migration to productive Antarctic waters. The radiation of early skim feeders such as putative late Oligocene balaenids may have contributed to the demise of *Waharoa* and other eomysticetids at the end of the Oligocene.

## Supplemental Information

10.7717/peerj.1129/supp-1 Supplemental Information 1 Electronic Supplementary Information Electronic supplementary information for RW Boessenecker and RE Fordyce. Anatomy, feeding ecology, and ontogeny of a transitional baleen whale: a new genus and species of Eomysticetidae (Mammalia: Cetacea) from the Oligocene of New Zealand.

## Institutional Abbreviations

OU: University of Otago Geology Museum, Dunedin, New Zealand

## Additional Information and Declarations

### New Species Registration

The following information was supplied regarding the registration of a newly described species: Zoobank LSID urn:lsid:zoobank.org:act:3601D851-F9D2-4364-83F5-7A466DC432F3. LSID urn:lsid:zoobank.org:act:2FC4AC75-2378-4FFA-801A-2A3475BFDF31.
