# Supplementary material for: Anatomy, feeding ecology, and ontogeny of a transitional baleen whale: a new genus and species of Eomysticetidae (Mammalia: Cetacea) from the Oligocene of New Zealand
Source: PeerJ. 2015 Sep 10;3:e1129. doi: 10.7717/peerj.1129 (PMC4570844; doi:10.7717/peerj.1129)
Supplement: Supplemental Information 1 — Electronic supplementary information for RW Boessenecker and RE Fordyce. Anatomy, feeding ecology, and ontogeny of a transitional baleen whale: a new genus and species of Eomysticetidae (Mammalia: Cetacea) from the Oligocene of New Zealand. [file peerj-03-1129-s001.doc]

**ELECTRONIC SUPPLEMENTARY INFORMATION**

R.W. Boessenecker and R.E. Fordyce. Anatomy, feeding ecology, and ontogeny of a transitional baleen whale: a new genus and species of Eomysticetidae (Mammalia: Cetacea) from the Oligocene of New Zealand.

1. List of institutional abbreviations.

**Institutional Abbreviations**: **AMP** - Ashoro Museum of Paleontology, Ashoro, Japan; **CAS/CASG** - California Academy of Sciences, San Francisco, California, USA; **CCNHM** - Mace Brown Natural History Museum, College of Charleston, Charleston, South Carolina, USA; **ChM** - Charleston Museum, Charleston, South Carolina, USA; **FMNH** - Field Museum of Natural History, Chicago, Illinois, USA; **GMNH** - Gunma Museum of Natural History, Tomioka, Japan; **HMN** - Hiwa Museum for Natural History, Hiwa, Japan; **IRSNB** - Institute Royal des Sciences Naturelles, Bruxelles, Belgium; **JGU** - Institut für Geowissenschaften Paläontologie, Johannes Gutenberg-Universität, Mainz, Germany; **KMNH** - Kitakyushu Museum of Natural History, Kitakyushu, Japan; **LACM** - Natural History Museum of Los Angeles, Los Angeles, California, USA; **MB -** Museum für Naturkunde, Berlin, Germany; **MCZ** - Harvard Museum of Comparative Zoology, Cambridge, Massachusetts; **MFM** - Mizunami Fossil Museum, Gifu, Japan; **MGPT** - Museo di Geologia e Paleontologia, Torino, Italy; **MGB** - Museo Geopaleontologico G. Capellini, Bologna, Italy; **MNHN PPI/SAS** - Museum National d'Histoire Naturelle, Paris, France; **MPST** - Museo Paleontologico di Salsomaggiore Terme, Italy; **MSM** - Museum Sønderjylland, Gram, Denmark; **MSNTUP** - Museo di Storia Naturale e del Territorio, Pisa, Italy; **NHG** - Natuurhistorische collecti van het Zeeuws Genootschap der Wetenschappen, Middelburg, Netherlands; **NMB** - Natuurmuseum Brabant, Tilburg, Netherlands; **NHMUK** - The Natural History Museum, London, UK; **NMNH** - Academician V.A. Topachevsky Paleontological Museum of the National Museum of Natural History, Kiev, Ukraine; **NMNS** - National Museum of Nature and Science, Tokyo, Japan; **NMNZ** - Museum of New Zealand Te Papa Tongarewa, Wellington, New Zealand; **NMRA** - National Museum of the Adygea Republic, Maykop, Russia; **NMV** - Museum Victoria, Melbourne, Australia; **OM** - Otago Museum, Dunedin, New Zealand; **OU** - University of Otago Geology Museum, Dunedin, New Zealand; **PIN** - Paleontological Institute, Moscow, Russia; **SCMNH** - Santa Cruz Museum of Natural History, Santa Cruz, California, USA; **SDNHM** - San Diego Natural History Museum, San Diego, California, USA; **SFMCV** - Shinshushinmachi Fossil Museum, Shinshushinmachi, Japan; **SMNK** - Staatliches Museum für Naturkunde, Karlsruhe, Germany; **SMNS** - Staatliches Museum für Naturkunde, Stuttgart, Germany; **TNU** - Taurida National University, Simferopol, Ukraine; **UCMP** - University of California Museum of Paleontology, Berkeley,

California, USA; **UM** - University of Michigan, Ann Arbor, Michigan, USA; U**OMNH** - University of Oregon Museum of Natural and Cultural History, Eugene, Oregon; **USNM** - National Museum of Natural History, Washington, D.C., USA; **ZMA** - Zoölogisch Museum, Leiden, Netherlands.

**2. List of specimens examined. Asterisk denotes taxa examined from literature or photographs.**

***Zygorhiza kochii***

USNM 11962

USNM 16638

USNM 16639

USNM 21443

USNM 25210

***Basilosaurus* spp.**

UM 97507

USNM 4674

USNM 4675

USNM 6087

USNM 12261

***Dorudon atrox****

UM 94812

***Simocetus rayi***

USNM 256517

***Waipatia maerewhenua***

OU 22095

**Charleston toothed mysticetes**

ChM PV 4745

ChM PV 5720

***Mammalodon colliveri***

NMV P204379

***Janjucetus hunderi***

NMV P216929

NMV P229455

***Chonecetus goedertorum***

LACM 131146

LACM 138027

***Aetiocetus cotylalveus***

USNM 25210

***Aetiocetus polydentatus***

AMP 12

***Aetiocetus weltoni***

UCMP 122900

***Eomysticetus whitmorei***

ChM PV 4253

***Micromysticetus rothauseni***

ChM PV 4844

ChM PV 5933

CCNHM 169

***Yamatocetus canaliculatus****

KMNH VP000017

***Tohoraata* spp.**

OU 22178

OM GL 409

***Tokarahia kauaeroa***

OU 22235

***Tokarahia lophocephalus***

OM GL 412

OM GL 443

OU 22081

***Waharoa ruwhenua***

OU 22044

OU 22075

OU 22140

OU 22163

***Caperea marginata***

OM VT 227

NMNZ MM002064

NMNZ MM002119

***Miocaperea pulchra****

SMNS 46978

***Balaenella brachyrhynus****

NMB 42001

***Eubalaena glacialis***

FMNH 15559

NHMUK 1891.9.12.1

USNM 269161

USNM 504343

USNM 504886

USNM A23077

***Eubalaena shinshuensis****

SFMCV 0024

***Balaenula astensis****

MSNTUP I12555

***Balaena mysticetus***

IRSNB 1532

LACM 54479

NHMUK 1934.10.10.1

USNM 15695

USNM 63300

USNM 63301

USNM 255992

USNM 291101

***Balaena ricei****

USNM 22553

***Joumocetus shimizui***

GMNH PV2401

***Metopocetus durinasus***

USNM 8518

***Vampalus sayasanicus****

PIN 5341

***Kurdalogonus mchedlidzei****

NMRA 10476

***Cetotherium riabinini****

NMNH P668/1

***Cetotherium rathkii****

PIN 1840/1

***Brandtocetus chongulek****

TNU skull A

TNU skull B

TNU skull C

***Herentalia nigra****

ZMA 5069

***Piscobalaena nana***

MNHN PPI 259

NMNH PPI 260

MNHN SAS 892

MNHN SAS 1616

MNHN SAS 1617

MNHN SAS 1618

MNHN SAS 1623

MNHN SAS 1624

SMNK PAL 4050

***Nannocetus eremus***

UCMP 26502

***Herpetocetus transatlanticus***

USNM 182962

USNM 183074

USNM 183075

USNM 183077

USNM 299653

USNM 299655

USNM 299656

USNM 312542

USNM 312543

***Herpetocetus bramblei***

SCMNH 9990.04

SCMNH 9990.06

SCMNH 9926.29

SCMNH 21229

SCMNH 21230

SCMNH 21236

SCMNH 21638

UCMP 82465

UCMP 219079

UCMP 219109

UCMP 219110

UCMP 219111

UCMP 219112

UCMP 129113

UCMP 219124

UCMP 219125

***Herpetocetus sendaicus***

NFL 17

NSMT VP-159540

***Herpetocetus morrowi***

SDNHM 65781

SDNHM 130390

UCMP 129450

and many additional referred specimens reported in El Adli et al. (2014:404-407)

***Aglaocetus moreni****

FMNH P13407

***Aglaocetus patulus***

USNM 23690

USNM 13472

***Titanocetus*** ***sammarinensis****

MGB 1CMC172 9073

***Isanacetus laticephalus****

MFM 18004

MFM 28501

***Diorocetus chichibuensis****

SMNH VeF19

SMNH VeF68

***Diorocetus hiatus***

USNM 16783

USNM 23494

***Cophocetus oregonensis****

UOMNH 305

***Uranocetus gramensis****

MSM p 813

***Parietobalaena campiniana****

RBINS M.399-R.4018

RBINS M.2010

RBINS M.2011

***Parietobalaena palmeri***

USNM 10668

USNM 10677

USNM 11535

USNM 16119

***Parietobalaena yamaokai****

HMN F00004

HMN F00022

HMN F00042

HMN F00064

HMN F000127

HMN F000640

***Pelocetus calvertensis***

USNM 11976

***Gricetoides aurorae****

USNM 25762

USNM 182921

USNM 183004

***Eschrichtioides gastaldii****

MRSN 13802

***Eschrichtius robustus***

AMP R09

NMNS M15940

NMNS M25899

USNM 13803

USNM 364973

USNM 364975

***Archaebalaenoptera castriarquati****

MGPC SBAER 240536

**"*Balaenoptera*" *cortesi* var. *portisi***

MCZ 17882

MGPT 13803

UCMP 219135

***Diunatans luctoretmurgo****

NHG 22279

NHG 22347

***Parabalaenoptera baulinensis***

CASG 66660

***Plesiobalaenoptera quarentellii****

MPST SBAER 240505

***Protororqualus cuvierii****

"Skeleton from Mount Pulgnasco", no number given, see Deméré et al. (2005:118)

***Megaptera hubachi****

MB.Ma. 28570

***Megaptera miocaena***

USNM 10300

***Megaptera novaeangliae***

MSNTUP M263

NMNS M25929

NMNS M33734

NMNZ MM000228

NMW Z.1982.058

OM VT532

USNM 13656

USNM 269982

USNM 301636

***Balaenoptera siberi****

SMNK (no number)

SMNS 47307

***Balaenoptera acutorostrata***

CAS 22180

ChM CM1183

MSNTUP M260

NHG 22426

NMNS M25927

SDNHM 23642

USNM 61715

USNM A13877

***Balaenoptera bertae***

UCMP 131815

UCMP 219078

***Balaenoptera bonaerensis***

NMNS M19792

NMNS M24357

NMNZ TMP 002188

OM VT3057

OM VT3060

USNM 504952

***Balaenoptera borealis***

NMNS M03536

NMNS M25908

SMNS 26443

USNM 236680

USNM 504244

USNM 504698

USNM 504699

USNM 571340

***Balaenoptera brydei***

NMNS M03538

NMNS M27007

NMNS M34505

***Balaenoptera musculus***

MSNTUP M250

NHMUK 1892.3.1.1

NMNS M25900

NMNS M29617

USNM 124326

USNM 239280

***Balaenoptera omurai****

NMNS M32505

NMNS M32992

***Balaenoptera physalus***

MSNTUP M251

NMNS M25904

OM VT2570

USNM 16039

USNM 237566

USNM 301635

USNM A16045

**3. List of cladistic codings.**

***Zygorhiza kochii***

000000000000000000000000?00000000000000000000000000000000000000100000000000000000[01]000000000000000000?000000000000000000000000000000000000000000?3000000000000000001000000000100010000?000000000000000000000000?000000000000?000000?0000000000000000000000000000000000000000000000000000000000002000100000000000000001????00000000000?0?0?0000100000??????0?????????????????

***Basilosaurus* spp.**

000000000000110000000000?00000000000010000000000000000?000000001000000000000000000000000000000?000000000000000000000000000000000000000000000000?000000000000000130000000000?100010000?000000000000001?00000000?00000000000000000000000001??000???0?00?0??0?00000000000000000000000000000000100001001000000001000000100??0000000000000000?000000100?????????????????????????

***Dorudon atrox****

000000000010010000000000?0?0000000000100000000000000000000000000000000000000000000000?00000001?0?0000000000000000000000000000000000000000000000?0000000010000000300001000010100?1?000?000000000000000?00000000??000000000000000000?0001000000000000000000000000000000000000000000000000000000002200100000000000000000000000000000000000000000000000????????????????????????

***Simocetus rayi***

?0100010?00130001000?110?00???000002000002100000000011?001100101000000010000?1000000010020001000000010000000000000100000000000000000000020000?0000000000???00000????1?10?1???1?01???????0?00??0??00000?00?00????0??????00????????????????????????????????????0000000???????00?????0000??00100?011001010????????????????????????????????????????????????????????????????????

***Waipatia maerewhenua***

?0110010?00130000000?110?00???000012000002100000000111?001110104000100010003?10001000110200010?0?00010000000000000100000000001000000000020000?0?0000000000000000301101100110110010000?000000000000000000000000??0??000000100000000?010000?1000000011??00?0???000000000000000000000000000001000011101010???0000?????????????????????????????????????????????????????????????

**ChM PV 4745**

?10??1?100?????01102?0?0????000??0020??002010?0?0010101?1??0?0?????00000010000000?000000000001001000?0100102000200100000000000001000001000000????100000000000000201201100110110021010?000000000000010000000001?000021100000???000?0000001100000?00000????00?????????????????????????????011??0022111100????????????????????????????????????????????????????????????????????

**ChM PV 5720**

?100?1020000200001?2?000???0000??00?01?002000?00001011??10011001000000000100000000000000000001?0100???1?01020002?0200000000000001000001000200????100000000000000201201000110110011010?000000000000010000000001?000020100000?0?00000000001000000000000000000??0000000001?200000000?0000??0111000221111?0????????????????????????????????????????????????????????????????????

***Mammalodon colliveri***

?10100000?10?01121010100?10000000012000000010?1110110??00210000100011000?100000011100000100010?010001000000?001000?00?000000??00????00??0????????10000100000000030221010011?11?010010?00000?000000001110000000?000020000010?00000?000010111000000001000?010?00001000000?000000000?0??0??011?0101?1?1?0000?????1????0????????0????????????????????????????0?????????????????

***Janjucetus hunderi***

?10101010010111111010100?100000000110000000000111011010000100001000110001100000011100000200011?00000100000010010001000000?0000000?00000000000?00?1000100???00000???21010?1???1?02?01??0?0?0????0?00000?0??00????0002???0?1?10000???000?????0000000?10?0??1???0001000000?00?00000000??0?00110?10111110?0??????????????????????????????????????????????????0?????????????????

***Chonecetus goedertorum***

01011101000021021?020101?10000000001010000000000001101010201000100010000110000001101010010001110100000001002001200100000000001000?10000100100?0?31000000???00001???20?1000???1?010000?000?00??00?000???0??00????0?0???0?0??1000000?00010111000000001000??10??0101010000?10000?00000101??0??0021??????1?00?00??1010011?????010?00010000000012010101?????????????????????????

***Aetiocetus cotylalveus***

0001110100001002110201012100000000010000020000000011010102000002000110001100001010010000200010100000001000021010000120030000010000100001?01002003101????????00????????????????????????0???0????????????????????????????????000?0?0?01010???0000000?1000?00??????????????????????????????0111?210011111100?????1?0?0000?1??120??????????????????????????????????????????????

***Aetiocetus polydentatus***

?0011101000010?21102010??10000000002010002000000?0100?01020110020001000001000010?1010000200010?00000?0100002101010?12003000001?000100001?0100200?101?????????0????????????????????????0???0????????????????????????????????00??0???01010???0000000?10?0??0?0001020100000100000000?0101??011??2101?11011000011110100100[01]111??0?0?1?1101???01??10?01?????????????????????????

***Aetiocetus weltoni***

??01110100001002110201012100100000010100020000000010010102011002000110001100001011010000210010101001?0?0?0011?1?000120030000010000100001?01002003101?00????0000????????????????0??????0?0?0???00??0?????????????0??????????00000000010101010000000010000000??0102010000010000000000101000?11121011?1?11????????????????????????????????????????????????????????????????????

***Eomysticetus whitmorei***

?01110010??02032???2?1?10101?00??01??1?002011?0000211???1221100100000100?010??10010??100100001??0000?01010110010001102000000111011100001101102???101000000000001101210101111110110010?000000000001000001001000?00001000010010000000001001110000000110000100??0102010001?200100000?0??100???????????????0??111?1?1??0?1010000??????00?001?112010101?????????????????????????

***Micromysticetus rothauseni***

?10110?101?02032???2???12??1?00??01??11?020?1?0100211???11200001000??100?010101000001100100001?0000000100012001010110200000011101110000110100????111001010000001102201101011110110010?00000?000000000000001000?000010000100????00?000?000?1000????110????10??010201?????????0?????02010????????????????01210101000010?????00???????0?0???????1?1???????????????????????????

***Yamatocetus canaliculatus****

?1011001011020321102010101010000001201100201100100211?0012200010003001000000?01001001100100001?00000?0100012001210110200100011101110000110100000?101?????????0?????????????????????????????????????????????????????????????1000000?00000?11000000010000?1000?010201000002001000000010100???????????????0021110101001001100????00110000011100010101?????????????????????????

***Tohoraata* spp.**

?1?????1???????????????????1?00??01??1?0???1?????0??????1??0???????0??000010101011001100?00?11??00?????010?10010001??2001000?1100???000?10?10????101001010000001101210110103111011121?00000?000001000001001110?000010010?11?000000000000[01]110000001100000110????0???0?0?0?00??00??0????00???????????????012111?101?110111???????????????????????????????????????????????????

***Tokarahia kauaeroa***

?101?0000??020321??2????????000??01??1100201??0100211???11200010000??10000101010110??100100001??0????0101?1?00101110020??0??1???0?110001?01?????????001000000001111211110102011011120?0000?0000000010100000011?000010020101?0000000001001110000001110000110??010201??0??2??10?000?0111?????????????????012101????01??10[01]10??111011100101111?010101?????????????????????????

***Tokarahia lophocephalus***

??????0101?????21??2010121?10000001??1?002001?0??021???0?121??1?000?0?0?0010?010?10???00100001???0????101011?0?001100200000011100011000110110????101001000000001101210110102011011120?00000?0000000[01]0100000011?000010020101?0000000000001110000001110000110??0102010001?200?0?000?0211??0??????????????002111010101101?111??11?????????????????????????????????????????????

***Waharoa ruwhenua***

?101?0010110203211?201012??1?00??01??11002011?0100211???1[12][02]000[0 1]000000100?0101010110011001000[01]1??0000?010[01]112001011100200100001100011000110110????10100101000000110121011010301[01]011121?00000000000100000[01]00[01]110?0000[01]0010111?000000000010[01]1100000011[01]0000110??010201000102001000000021100???????????????012[01]11?100?0[01]11[01][01]010011????10???1??00010101?????????????????????????

***Caperea marginata***

0020000003102033111201011101300001220010000121010021111012130101012001010211002001011101212011300101?1401120100110102?001010020?00??102?110111121110010000011111312?11002000?101400200000000001011101?10000000???00001?01?0011121111121100100002021?11001?001110221100214001000?200101101??????????????21110???001?1??0?1?1111001121111111120101101000000121111011000001000

***Miocaperea pulchra****

?0200000031020331??2?????1?130????2????000012?01002111?012?301010120?10102?100200?01110121?011400100?1401120100110100?101010020?00??102?11011???21110100???1111131??1?1020???1?12000?00?0?00??0011100??1?00??????0???0?01??????????????????????????????????????????????????????????????????????????????????????????????????????????????????????????????????0???????????????

***Balaenella brachyrhynus****

?020000003?020331102010121?130000021001000012?01?0211??01?13???????00201002100200?010101212011?00000?140012?1001102?201?1000?20?200010??11211??1210?0120???1?20?????1?1?21???1?????0????0?00???0?12?1??1????????2??????0????1?12??10?210?010100?011?1?1???1?????????????????????????????1??????????????????????????????????????????????????????????????????????????????????

***Eubalaena glacialis***

102000000310203311020101211130000022001011012101012111101113000100100201002100100101110121201110000[01]?04001201021102020121000020020001020112111212100012000011200302?1110210??11140000?000100010011201111200000?0200201?0110110120010021100100001111?10101?00?110221100214021100?200101101??????????????211102000?11??100111011100111011111021110100000001121110000000000001

***Eubalaena shinshuensis****

?02000000??0203311?2010121?13?00002??010?1?12?01012????0?1130001001??2?10?2100?001???1?12?201???00????40012?1021102??01?1?0??2???????0???121?????10??????????2??????????????????????????????????????????????????????????????????????????????????????????????????????????????????????????1??????????????????????????????????????????????????????????????????????????????????

***Balaenula astensis****

?020?00003?0?0??1??201?1?1?13000002?00?011012101?1211??0?1130001001002010021002001011101212011?00000?0400120102110202011100002002000102011211???21000120???1120030??????2100?111?0000?0?0?00??00112??11?0???????????????110?1??2??10021110101100011?101?1?0??1102?1?0?21???1100???0?????1??????????????????????????????????????????????????????????????????????????????????

***Balaena mysticetus***

10200000031020331102010121110000002200110101210100211110121300?1001002010021001001010101212011100000?04000201021102020121000020020001020112111212100012000011200312?11102110?11140000?000100000011201111200000?0200201?0110110120010021100100001111?10101?101110221100214021100?200101101??????????????2?1??0?00?11??1???10011000011011111021100100000001122110000000000001

***Balaena ricei****

?02????????0???31???01?12?????0??02????10?0?2?0100211??????3?????????2?1??2?00?????????12?201????0?????0002?10?110????1?1????2???????0??????????????01200001??0?302?111021???11??0000??0010?000011201011200000?1200000?0??0??????????????????????????????????11?221????????11??????101??1??????????????211100100?11??110????1100011101111102110010?????????????????????????

***Joumocetus shimizui***

?001000102102232111201?101?00110002210200?011?010021110002100101100??1?102?0?02001?11101210011?00?0??000??0010001?????????????0??????????????????1??0????????1?1????1?1??????1??2????00?????????????????????????????????????1??11??11211?110010?011??????10??110211?????????0??????101??1??????????????0???????????????????????????????????????????????????????????????????

***Metopocetus durinasus***

?0?????1???????????20???????2?1000??002??????????0???????21?010?10?????1?????0???1???1??2?0111?00001?01011001000?0????0110?0?2010???10??10?1??1?2???010010112110302?1100210??1012000001100011000?1101110000000?1000001?0110????????????????????????????????????????????????????????????????????????????????????????????????????????????????????????????????????????????????

***Vampalus sayasanicus****

?0?????0????????????0?????????1000??????0????????????????2???1?????0?????????????10??10?2?0?1???01?1??2??1?1?0???010?000?0?00201000010101011????????01001011211?302?1100210??10?100?01110001100?0?100?0?00000????00?00?0?10????21?11?2110110?1??0?1????????????????????????????????????????????????????0???????????????????????????????????????????????????????????????????

***Kurdalogonus mchedlidzei****

?00????????0???211??01?1????001000221?20???1??????2?1???1??0???????00101?010002?01011101200011?00000?0201110100000102000200002010000101010110????1110120???12111302?1?0021???1?11002?11?0?01???0??1?0?01?0000???0??0???0?????????????????????????????????????1?02?1?????3??10??1??0????????????????????????????????????????????????????????????????????????????????????????

***Cetotherium riabinini****

100000011210223211120111010021100022002000011101002111??12110102100001010012?02001011101200011100001?010110010000010200020000201000010101001021?2111???????121??????????2????????????11????0???????????????????????????????01112?1?1?21??110?10001??000??????1102111002131010100000101101??????????????0?????????????1001100??10112111011102010011?????????????????????????

***Cetotherium rathkii****

?00??0011??0?2?2?1120111?1001110002?0020?0011001?02???001211010?10300?010012002001011101200011?00001?01011001000?010200020?0020100001010100100102111012010?1211130??1?0021???1?11002?1110?00???0??1001010000????00?0?0?0???1111211?1021??110?10001??000?0????11?2?1????????1????????????1??????????????????????????????????????????????????????????????????????????????????

***Brandtocetus chongulek****

?0?????????????????10???????111000???02??????????????????2?1010?10?????1?????01??1?1?101200011200100?01011011000102???002001?2010???00??1??102??2111012011012111302?111021???1?11000011?00?0000001101001000?00??200?00?0111?111101?10211011[01]0000011?0100110????????????????????????????????????????????????????????????????????????????????????????????????????????????????

***Herentalia nigra****

?0?????????????????11???????111010??102??????????????????2??010?10?????1??????1??1???10?2?0111?00102?0101100100010????????0??20?????????1???????2111?100????2111????????21??????4????01?0?00??10??1????????????????????????????????????????????????????????????????????????????????????????????????????????????????????????????????????????????????????????????????????????

***Piscobalaena nana***

0001000112102232411211110100212010221020020111010021111012110102112011010113?01001011101210111100002?01011001100101020001001020102001000100100102111010010112110302?11102100?101200200100101100001101001000000?0000001?011?0111111?1021100111011011?0100210?11102111102131010111000101111??????????????011201001001011001111??00112111011102010011?????????????????????????

***Nannocetus eremus***

???????1?????????????????????????0????2??????????0???????2????????????01?2??1?1001??110120?????0?012??1??1?0?????010?00010?1020100001020110111????11010010112111302?1110210??10110020011000100000110??01000000?0000000?011001?1111?1?21101101000011?010?110????????????????????????????????????????????????????????????????????????????????????????????????????????????????

***Herpetocetus transatlanticus***

?0????????1????????2??????????2??????0??????1???????????????????1??????1?????0??0????101???11?11001??????1?0??0??010?00010?1020100001010110111??2111012010112111302?11102100?101100000101101100001101001000000?1200001?0110?11111111021101101001011?0100110?????????????????????????????1??????????????????????????????????????????????????????????????????????????????????

***Herpetocetus bramblei***

?001000112?02232411211110100212010221020020110010021110012100102112111010110?01001011101202111110012?01011001100101020001001020102001010110111102111012010012111302?11002100?101200000101011000001101001000000?1200001?0110011110111021101111011011?0100110??1102111102131010111000101101??????????????????????????????????????????????????????????????????????????????????

***Herpetocetus sendaicus***

0001000012?0?23???121111010011201022102002?11?01?02??1?0?21301011121?101?111?01001?11101202011110012?02011101100?01020001001020100001010110111102111012010012111302?1???2100?10110020010101100000?101?0?000000?1200001?01101111101?1021101101000011?01001?0??1?02?11102131010111000???1?1??????????????0102010010?1101001110??00112??????102?10110?????????????????????????

***Herpetocetus morrowi***

?00100001210223241120111010011201022102002011001002111?012130101112101010111101001011101210011110012?02011101100101020001001020102001010110111102111012010112111302?11002100?101200200101111100001101001000000?1200001?0110011110111021101111001011?01001????1102111102131010111000101111??????????????0111012010?1101?0???????????????1???????????????????????????????????

***Aglaocetus moreni****

?1010001021021323112010101000100002?0020020120010021110011100102000001010010002001011101210011200101?020110010021020200111?0020120001010?011221?3111???????1?2??????????2?????????????0?0??0?????????????????????????????????????????????????????????????????111211???0121010000011101101??????????????????????????????????????????????????????????????????????????????????

***Aglaocetus patulus***

?001000102102122311201010101000000220110020120010021110012100102000001010010003001011101210011?00001?03011101002102020011100020120001000101122003111010100011210302?11102000?10110000?0?00000?1011201111100001?0020211?0111?111111?1021101111001011?0100110???????????????????0?????????1??????????????0?12020???????110??1?????0??????????????????????????????????????????

***Titanocetus*** ***sammarinensis****

?00100010?10212211?2010101001100002?0?2002012001002111?011110102000001010010?020010??1012100112?0?00?0100110100010102000100002012000101010?1221??11?????????????????????2?????????????0????0???????????????????????????????????????????????0?????????????????1112010101?2?010001001101101??????????????01101??01???0?1?????????????????????????????????????????????????????

***Isanacetus laticephalus****

?00100010210?122211201010100010000220010020120010021110012100101000001010010?02001010101210011400000?02011001000102020001000020120001000100122102111010001011210302?10102100?10110000?000100000001201111000000??000011?01110111211?1121101100101011?01001?0??11?211????????10??????101??1??????????????01?????0??110111011?????????????????????????????????????????????????

***Diorocetus chichibuensis****

?001000102102122?11201010101000000220?100201100100211100111001020?0001010010?02001011101210011200000?010111010021010200111?002012000100010112?00???1?????????2?0????????2????1??????????0?00??00??2?????????????????????????11?211?1021101100101011?0100110??11?2010101121010001011101??1?????????????????????0??????100???????????????????????????????????????????????????

***Diorocetus hiatus***

0001000102102132311201010100000000220?100201100100211100121001020??001010010?0?00101110121001130000000101100100210102002100002012000?0001011200?3111010000011211302?11102110?10120000?000000011011100011100000??00?000?0211210101111021101100001011?0?00?10??1102110?01121010001010101101???????????????1?????0?01101?001110??0011????????02010010?????????????????????????

***Cophocetus oregonensis****

?00100010?10212221?201?101000100002?001002011001002111001210010?000?0?010??0?0200101?1012100112?01?1??20110010?21010?00011000201000010?0101120103111010000011210302?1?102?1??1?110010??001??0?10?12???11?0000??0000????011021??????11211????????????0?0??????1102?10101121010001010???101??????????????01?????0?0??1??????11???011210101?1????00100????????????????????????

***Uranocetus gramensis****

?001000?02?0213221?2010101010110002?0?2002011001002111?012??0101100?01010010?02001011101210111??0100?02?11001002?020??0211?002010?001010102122???111010?00?112?0302?1?102????1?13001??0?00000?10?12???11?000????000200?0?112111111?1121111100000011?0000??0??1112?1000112?010001000???101??????????????01??0??010??1?1?01???1100012111011102010011?????????????????????????

***Parietobalaena campiniana****

??01???????????2111??10101????????2????00001???10?2111??1??????????00101001?10200?011?012??????0??00???????0?????0102000100002012000100010210???2111010100111210302?111021???1?110000?000000011011200111000000??000?11?0110?1?111?11121111101000011?0?????0??1?12?10101121010001000???101??????????????01120?2???????11011?????????????????????????????????????????????????

***Parietobalaena palmeri***

00010001021021323112010101010000002?0010020120010021110011100101000001010010102001011101210011300000?0101110100010102001100002012000100010210?0?3111010100111211302?11102100?11110000?000100011011201111100000?0000211?01102111111?1121101101111011?0000010??1102110001121010001011101101????????????0?0112022110?001100??1??????????????1?????????????????????????????????

***Parietobalaena yamaokai****

?0010001021021223??201?1?10?0000002?0??00201100100211?001??0010?????01010010?0?001010101210011?0000???20111010021010?00210?0020120001000101122003111?1?????1?2????????????????????????0????0???0???????????????????????????2111111?1?21111?0?10?011?010?0????1102110101121010001001???101??????????????0?????????????100??1???00?1????????????0????????????????????????????

***Pelocetus calvertensis***

?0010001021021222112010101010100002100100201100100211100121001020?0001010010?0200101110121001130?1000?2011001002101020001100020120001000101?2?103111010????11210?02?1?1?21???1?12??2??0?0?00???0?1201111?00?????20?2???0???0111111?1121101101101011?00000?0111102?10?01121010001011101101??????????????011011011010001?10110??0100210101110201?010?????????????????????????

***Gricetoides aurorae****

??????????????????????????????????????????????????????????????????????????????????????????????????????????????????????0?10?0?2?1????10??1??1??1?2111110001011210302?1?1021???1?12000??00010000?0?11?011121000?0??20000?2110?1?121?11121111101100011?000?1?0????????????????????????????????????????????????????????????????????????????????????????????????????????????????

***Eschrichtioides gastaldii****

?00?00010??0?2?2?11201?101001100002200?0??012001?0211?0012?10102000001120210001101?11101210011?00001?0111111110210?0?00?10?00201000010101121??1?2111???????1?2??????????2??????????????????0????????????????????????????????11?21??1?????????????????????????1112101012121110001200101101??????????????01120??010?0111?0??????????21010111????0000?????????????????????????

***Eschrichtius robustus***

00000002021022321112010131021100002201?000012001002111001211010201000112021000110101110121101110000[01]?01111111102101020011010020100001010111130102111110011011210302?11102100?10110000?0001000000111?111121000200?20000?2211011121111121111101101011?00000?01?111211101214111000?200101101??????????????0?1????01?10??1??10101100?121010111121100101000100110000000000000000

***Archaebalaenoptera castriarquati****

?001000102?0222211121101?1?21100002200200202??0100211??012110103000001120212103?010??101210111?00?00?020111?1100102010011?10020?0?001010?0113????????????????????????????????????????????????????????????????????????????????????????????????????????????????1112?1?????2?0?0?00???10?1?1??????????????????????????????????????????????????????????????????????????????????

**"*Balaenoptera*" *cortesi* var. *portisi***

?0???????????22????????????2000?102????0??????????21????1??0???????00?12?21?1?300101110121?111??0000?010212011021020200010?0020100001000102132?????1110010011211302?11002100?10110020?000000000011101?1120000210100000?1211??11211?1121?01100000011?00012?0??111211100222?0100010001011?1??????????????01101??011?1101001?10??0011?1010111????0?10?????????????????????????

***Diunatans luctoretmurgo****

?0?????1?????2??????????????000111???02??????????????????210010?010????2?????????1???1?121111?200000?03011201102?010?10111000201000010101021321?2111110001011210302?1100210??10120020?000100210001????110000021?111000?2?1001?1211?11?1?0110?1????1?0??12?0????????????????????????????????????????????0???????????????????????????????????????????????????????????????????

***Parabalaenoptera baulinensis***

?001000102?02232??1211?1?1?2110000220020020220010021??0012110102010001120212103001011101211111??00?0?020212011021010210111?0020100001010?021321??111???????1?2?0????1???2????1????????0???00??????????????????1?1??????????01????????2?1?1????000???000??????111211???2221010001100101101??????????????01??0???1???1?0????1???0????111011?12???????????????????????????????

***Plesiobalaenoptera quarentellii****

??0?????02?022?221???1?????2??0???220?200?????????211???1???????????????????1???????????????????????????????????????????????????????????????????????110000011210302?11002100?10120020?0000?021000??0??11000002101110?0?2200??11211?1121?11101101011?00010?1??1112111002221010001?00101101??????????????0??10?2???????1?????011?????????????????????????????????????????????

***Protororqualus cuvierii****

0??100010?10222211121101?10211000022002002022001002111?01211??0200000112021210?001011101211111??00???020211010001020100111?002010000100??01??2???111????????????????????2?????????????0??????????????????????????????????????????????2???????????????????????1112111?02221010001000??11?1??????????????0?1????0?????????????1100??2???????????0????????????????????????????

***Megaptera hubachi****

000100010?102222111211?1010201000022002002022001002111?012100102010001120212103001011101211111?00000?020212011020020100111100201000010101021321?2110110????11210????1?0021???1?110020?0?0?00???0?1?0???1?1?0??100??????22?1????????????????0??????0?????????11112??10022210?00010001011?1??????????????0?12?2201?11??1??111011001121110111120100101????????????????????????

***Megaptera miocaena***

?00??0010??0?2?2?112?1010102000000?20020?2022001?02????012100001000001120210102001011101211111?00000?030212011020020210211?002010000101010113?1?21111100???11210302?1100210??10120020?000100000001??1?1121000?00120000?1?1101?111??1101111100001001?000???0?????????????????????????????1??????????????????????????????????????????????????????????????????????????????????

***Megaptera novaeangliae***

0001000102102232111211013102010000220020000220010021110012100102010001120212103001011101211111400001?03021201102002021021110020100001010101132102111110000011210302?11102100?10120020?00000000002110111121000210020000?1211011111111121101101110011?0001??0111112111002221010001100101101??????????????01110200001010100111011111121011111121110101211100112000000000001001

***Balaenoptera siberi****

000100011??0223221122101010211011122002002022001002111?0121001030100011202131030010??101211111?00000?020112?10021010210011?0020?0?001010?011??1??11??????????2????????????????????????????00????????????????????????????????1??211?1??1?11101111021?000?0?0??111211????22?01?00110?10?1?1??????????????0?1??2001?11??1??111011001121010111020100101??????1?????????????????

***Balaenoptera acutorostrata***

0000000102102222111221010102010110220020020220010021111012100103000001120212103001011101211111200000003021201102101021001110020100001110102132102111110000011210302?11002100?10140020?00000000011110111121000210020300?1211011121111121001102111011?00012?0111112111002221010001000101101??????????????0112020010101110011101100112111111102010110111111011[1 2]001112013213210

***Balaenoptera bertae***

?0?????????????????????????2000?10??????????????????????1?10???????00?12?2101?3001011101211111?00000?03021201102101021001100020100001110102132122111???????1121?????????2?????????????0???00???????????????????????????????????????????????????????????????????????????????????????????????????????????????????????????????????????????????????????????????????????????????

***Balaenoptera bonaerensis***

0000000102102222111221010102010110220020020220010021111012100103000001120212103001011101211111?0000000302120110210102100111002010000111010213210?111110???011210302?11002100?10140020?00000000011110111121000210020300?1211011121111121001102111011?00012?0111112111002221010001000101101??????????????0112022010111110011101100112111111112010110111111011[1 2]000211113113212

***Balaenoptera borealis***

0000000102102232111221010102010110220020020220010021110012100103000001120212103001011101211111300000003021201102101021011110020100001110102132102111110000011210302?11002100?10140020?00000000112110111121000210000300?1111011121111121001102101021?00012?0111112111002221010001100101101??????????????011112001?111?100111011001121110111020100101111110112001111010012011

***Balaenoptera edeni***

0000000112102222111211010102010000220020020220010021110012100103000001120213103001011101211111200000002021201102101021001110020100001100102132102111110????11210302?11002100?10140020?00000000111110111121000211000000?1111011121111121001101111111?00012?0111112111002221010001100101101??????????????0111020010111010011101100112111011102110010121112011200????????????1

***Balaenoptera musculus***

0001000112102232211221010102110111220020020220010021111012110103000001120213103001011101211111300001002021201102101021021110020100001100102132123111110000011210302?11002100?10140020?00000000112110111121000200001000?1211011111111121001101100111?00010?1111112111002221010001100101101??????????????01120200101010100101011001121111111120101101211110112000020001100001

***Balaenoptera omurai****

000100011210223221122101010211011022002002022001002111001211010300000112021310300101110121111130000000202120110210102102111002010000111010213210311111?????1?210302?11002110?10140020?00000000002110111121000210000000?12110111211?1121001101111121?00012?1111112111002221010001100101101??????????????0??????????????????10??????2??111111201?010121111011000????????????2

***Balaenoptera physalus***

0000000112102222111221010102110111220020020220010021110012100103010001120213103001011101211111300001002021201102101021021110020100001100102132122111110000011210302?11102100?10140020?00010000112110111121000200001000?1211011121111121001102111111?00010?1111112111002221010001100101101??????????????011??200101010100101011001121011111120100101211110112002111111112122

**4. List of morphological characters used in cladistic analysis.**

1. Cranium, length. Modified from Bisconti (2000: 72), Deméré et al. (2005: 83), Bisconti (2008: 56), Marx (2011: 26), Churchill et al. (2012: 114).

0=less than 1/3 body length.

1=approximately 1/3 body length.

2. Cranium, dorsal profile posterior to orbit. Modified from Bisconti (2005: 63), Fitzgerald (2010: 55), Churchill et al. (2012: 1), El Adli et al. (2014: 8).

0=dorsal edge of skull ascends steeply towards vertex, vertex forms >10º angle between anterior edge of occipital shield and anterior skull bones

1=dorsal edge of skull is low or flat, forming <10º at the vertex

3. Cranium, facial plane. Modified from Bisconti (2000: 1, 2), Kimura and Ozawa (2002: 8), Geisler and Sanders (2003: 63), Dooley et al. (2004: 33), Deméré et al. (2005: 1), Bouetel and Muizon (2006: 1), Fitzgerald (2006: 65), Bisconti (2008: 33), Deméré et al. (2008: 1), Fitzgerald (2010: 37), Kimura and Hasegawa (2010: 1), Churchill et al. (2012: 1, 4), Bisconti et al. (2013: 5, 6), El Adli et al. (2014: 1).

0=straight

1=concave

2=convex

4. Premaxilla, anterior part in dorsal aspect. Modified from Fitzgerald (2006: 8), Geisler and Sanders (2003: 8), Bisconti (2008: 106), Fitzgerald (2010: 6), Kimura and Hasegawa (2010: 9), Marx (2011: 7), Bisconti et al. (2013: 33), Fordyce and Marx (2013: 4, 7).

0=narrows anteriorly or with parallel margins

1=anteriorly widens

5. Premaxilla, posterior part in lateral aspect. Modified from Bouetel and Muizon (2006: 2), Steeman (2007: 14), Marx (2011: 8), Fordyce and Marx (2013: 5).

0=shallower dorsoventrally or same depth as anteriorly

1=dorsoventrally deeper posteriorly

6. Premaxilla, overhang of maxilla. Modified from Kimura and Ozawa (2002: 17), Geisler and Sanders (2003: 110), Fitzgerald (2006: 9, 100), Bisconti (2008: 128), Fitzgerald (2010: 49), Marx (2011: 9), Fordyce and Marx (2013: 7).

0=do not overhang maxilla adjacent to narial fossa

1=premaxilla overhangs maxilla

7. Premaxilla, premaxillary foramen. Modified from Kimura and Ozawa (2002: 16), Geisler and Sanders (2003: 69), Fitzgerald (2006: 71), Fitzgerald (2010: 40), Marx (2011: 43), Bisconti et al. (2013: 32).

0=absent

1=present

8. Premaxilla, posterior extension relative to maxilla. Modified from Bouetel and Muizon (2006: 18), Fitzgerald (2010: 41), Kimura and Hasegawa (2010: 5), Marx (2011: 49), Bisconti et al. (2013: 34).

0=anterior to anteriormost supraorbital process of frontal

1=at level of anterior half or midpoint of supraorbital process of frontal

2=at level of posterior half of supraorbital process of frontal

9. Premaxilla, position of inflection in dorsal view. Modified from Geisler and Sanders (2003: 107, 108), Bouetel and Muizon (2006: 12), Fitzgerald (2006: 98), Steeman (2007: 6), Bisconti (2008: 81), Deméré et al. (2008: 18), Marx (2011: 53),

0=within posterior half of rostrum

1=at level of anterior edge or anterior half of supraorbital process of frontal

2=at level of posterior half of supraorbital process or postorbital process

10. Premaxilla, cross section of rostral portion. New character.

0=ventrally flat and contiguous with ventral maxilla

1=ventrally concave

2=medially and ventrally concave

3=medially concave, ventrally/laterally convex

11. Premaxilla, exposure in palate. Modified from Fitzgerald (2010: 13), Fordyce and Marx (2013: 17).

0=present posterior to anteriormost tip of maxilla, medial to maxilla

1=only exposed anterior to maxilla

12. Premaxilla, rostral process. Modified from Bisconti (2000: 10), Geisler and Sanders (2003: 68), Fitzgerald (2006: 70), Fitzgerald (2010: 39).

0=convex transversely

1=flat or concave, forming premaxillary sac fossa

13. Premaxilla, medial contact of premaxillae along anterior 2/3 of rostrum. Modified from Geisler and Sanders (2003: 5, 9, 67), Bouetel and Muizon (2006: 22), Fitzgerald (2006: 10), Fitzgerald (2010: 7), Kimura and Hasegawa (2010: 6), Bisconti et al. (2013: 13).

0=medially fused along most of rostrum

1=separated along most of rostrum, medially fused at anterior tip

2=separated along entire length of rostrum

3=widely separated, greater than or equal to transverse width of premaxilla

14. Premaxilla, posterior end. Modified from Geisler and Sanders (2003: 112), Fitzgerald (2006: 102), Fitzgerald (2010: 50).

0=faces anterolaterally

1=faces anteriorly

2=faces anteromedially

15. Maxilla, length of rostral portion. Modified from Kimura and Ozawa (2002: 9), Geisler and Sanders (2003: 3), Deméré et al. (2005: 3), Fitzgerald (2010: 2), Kimura and Hasegawa (2010: 26), Marx (2011: 1), Churchill et al. (2012: 3), Bisconti et al. (2013: 1), Fordyce and Marx (2013: 1), El Adli et al. (2014: 3).

0=intermediate, 40-49% of CBL

1=short, <40% of CBL

2=long, >50% of CBL

3=very long, >60% of CBL

16. Maxilla, lateral edge. Modified from Kimura and Ozawa (2002: 12), Geisler and Sanders (2003: 4), Deméré et al. (2005: 4, 5), Bouetel and Muizon (2006: 6), Fitzgerald (2006: 4), Deméré and Berta (2008: 45), Deméré et al. (2008: 3, 4), Fitzgerald (2010: 3), Kimura and Hasegawa (2010: 12, 13), Marx (2011: 3), Churchill et al. (2012: 7, 10), Bisconti et al. (2013: 26, 27), El Adli et al. (2014: 4).

0=thick lateral edge, >45* in cross section along length of maxilla

1=thin lateral edge posteriorly, <45*, anterior half of maxilla steeper and approximately 45* or more

2=thin lateral edge along entire maxilla, <45*

3=thin lateral edge posteriorly, transverse width of dorsal surface of maxilla in anterior half is less than ½ transverse width of ventral surface

17. Maxilla, lateral border anterior to antorbital process. Modified from Fitzgerald (2006: 3), Bisconti (2008: 107), Marx (2011: 5), Bisconti et al. (2013: 3, 4)

0=straight

1=continuously convex

2=posteriorly parasagittal, anteriorly straight and converging at rostrum tip

3=posterior 3/4 parallel and narrowing in anterior 1/4

18. Maxilla, steep face on antorbital process separating it from rostral portion. Modified from Geisler and Sanders (2003: 13), Bouetel and Muizon (2006: 9), Fitzgerald (2006: 14), Bisconti (2008: 7), Deméré and Berta (2008: 39), Fitzgerald (2010: 10), Kimura and Hasegawa (2010: 16), Marx (2011: 11), Bisconti et al. (2013: 10).

0=absent

1=present

19. Maxilla, anterior border of antorbital process. Modified from Fitzgerald (2010: 9), Marx (2010: 12), Fordyce and Marx (2013: 10).

0=maxilla closely approximates or underlies frontal

1=separated by a basin

20. Maxilla, posterior portion. Modified from Kimura and Ozawa (2002: 15), Deméré et al. (2005: 14), Deméré and Berta (2008: 46), Deméré et al. (2008: 13). Fitzgerald (2010: 44), Kimura and Hasegawa (2010: 19), Marx (2011: 14), Fordyce and Marx (2013: 12), El Adli et al. (2014: 16).

0=situated lateral to frontal

1=situated lateral to nasal

2=situated lateral to nasals and premaxilla

21. Maxilla, posterior end of ascending process. Modified from Geisler and Sanders (2003: 14), Deméré et al. (2005: 20), Bouetel and Muizon (2006: 12), Fitzgerald (2006: 15), Steeman (2007: 6), Bisconti (2008: 77), Deméré et al. (2008: 18), Fitzgerald (2010: 43), Kimura and Hasegawa (2010: 20), Marx (2011: 15), Churchill et al. (2012: 8), Fordyce and Marx (2013: 13), El Adli et al. (2014: 24).

0=lateral edges convergent, process tapers to point

1=lateral edges parallel

2=lateral edges divergent, process transversely expanded at posterior end

22. Maxilla, antorbital notch. Modified from Kimura and Ozawa (2002: 11), Bouetel and Muizon (2006: 8), Steeman (2007: 16), Bisconti (2008: 80), Kimura and Hasegawa (2010: 15), Marx (2011: 16), Churchill et al. (2012: 9), Bisconti et al. (2013: 10, 11), Fordyce and Marx (2013: 9, 14).

0=antorbital notch absent

1=antorbital notch present

23. Maxilla, antorbital process. Modified from Bouetel and Muizon (2006: 10), Kimura and Hasegawa (2010: 17), Marx (2011: 17), Bisconti et al. (2013: 8, 9, 10, 11).

0= does not extend anterior to notch

1=extends anterior to antorbital notch

24. Maxilla, nutrient foramina and sulci. Modified from Geisler and Sanders (2003: 17), Bouetel and Muizon (2006: 4), Fitzgerald (2006: 18), Steeman (2007: 2), Deméré and Berta (2008: 16), Deméré et al. (2008: 38), Fitzgerald (2010: 14), Kimura and Hasegawa (2010: 11), Marx (2011: 20), Churchill et al. (2012: 16), Bisconti et al. (2013: 12), Fordyce and Marx (2013: 19), El Adli et al. (2014: 50).

0=absent

1=present

25. Maxilla, geometry of lateral nutrient foramina and sulci. Modified from Deméré et al. (2005: 41), Deméré et al. (2008: 54), Marx (2011: 45), Churchill et al. (2012: 16), El Adli et al. (2014: 51).

0=posterior foramina with radially arranged sulci, no associated open maxillary groove

1=posterior foramina coincide with open maxillary groove

2=foramina restricted to posterior 2/3 of palate, with isolated posterior foramina without sulci and 1-2 bilateral pairs of large foramina with elongate sulci anteriorly

3=posterior foramina present in two rows without well developed sulci

26. Maxilla, infraorbital plate. Modified from Bisconti (2000: 71), Geisler and Sanders (2003: 59), Bisconti (2005: 5, 58, 64), Deméré et al. (2005: 17), Bouetel and Muizon (2006: 7), Fitzgerald (2006: 60), Bisconti (2008: 6, 141), Deméré and Berta (2008: 29), Deméré et al. (2008: 16), Fitzgerald (2010: 34), Kimura and Hasegawa (2010: 14), Marx (2011: 39), Churchill et al. (2012: 12, 13), Bisconti et al. (2013: 114), Fordyce and Marx (2013: 30), El Adli et al. (2014: 23).

0=absent

1=present

27. Maxilla, window adjacent to infraorbital plate. Modified from Deméré et al. (2005: 40), Deméré et al. (2008: 37), Marx (2011: 40), Churchill et al. (2012: 14), Fordyce and Marx (2013: 31), El Adli et al. (2014: 49).

0=absent

1=present

28. Maxilla, angle between anterior margin of supraorbital process of frontal and ascending process of maxilla. Modified from Bisconti (2000: 26), Kimura and Ozawa (2002: 13), Geisler and Sanders (2003: 49), Bisconti (2005: 59), Bouetel and Muizon (2006: 25), Fitzgerald (2006: 50), Steeman (2007: 9), Bisconti (2008: 110, 130), Kimura and Hasegawa (2010: 24), Marx (2011: 28, 42), Bisconti et al. (2013: 20, 21) , Fordyce and Marx (2013: 23).

0=larger than 90˚, frontal-maxilla suture trends posteromedially towards vertex

1=larger than 90˚, frontal-maxilla suture transverse or trends anteromedially towards vertex

2= less than 90˚

29. Maxilla, posterior edge of ascending process. Modified from Kimura and Ozawa (2002: 1), Geisler and Sanders (2003: 76), Dooley et al. (2004: 37), Bisconti (2005: 25), Deméré et al. (2005: 16), Bouetel and Muizon (2006: 11), Fitzgerald (2006: 78), Deméré and Berta (2008: 3), Deméré et al. (2008: 15), Fitzgerald (2010: 42), Kimura and Hasegawa (2010: 18), Marx (2011: 44), Bisconti et al. (2013: 18), El Adli et al. (2014: 22).

0=at level of anterior half of supraorbital process of frontal or in line with halfway point

1=at level of posterior half or at position of postorbital process

2=extends posterior to postorbital process

3=well anterior to orbit

30. Maxilla, posterior edge of ascending process. Modified from Marx (2011: 47).

0=anterior to orbitotemporal crest

1=posterior to anteriormost point of orbitotemporal crest

31. Maxilla, medial contact of ascending processes. Modified from Bouetel and Muizon (2006: 13), Kimura and Hasegawa (2010: 22), Marx (2011: 48), Bisconti et al. (2013: 23), Fordyce and Marx (2013: 34).

0=absent, maxillae widely separated

1=processes contacting at tips or nearly contacting

2=long medial contact of ascending processes, anteroposteriorly longer than dorsal exposure of nasal

32. Maxilla, posterior border of ascending process. Modified from Marx (2011: 50).

0=transversely oriented or medial corner more posterior

1=lateral corner more posterior

33. Maxilla, posterior border of ascending process. Modified from Geisler and Sanders (2003: 14), Bouetel and Muizon (2006: 12), Fitzgerald (2006: 15), Steeman (2007: 6), Bisconti (2008: 81), Deméré et al. (2008: 18), Marx (2011: 51), Bisconti et al. (2013: 18).

0=pointed or rounded

1=squared off

34. Maxilla, ascending process in dorsal aspect. Modified from Geisler and Sanders (2003: 129), Steeman (2007: 7), Bouetel and Muizon (2006: 21), Marx (2011: 60), Bisconti et al. (2013: 24).

0=posteriormost point anterior to supraoccipital

1=posterior to anteriormost point of supraoccipital

35. Maxilla and Mandible, open alveolar groove. New character.

0=dentition present, not implanted in alveolar groove

1=alveolar groove developed (dentition absent or present)

2=dentition and alveolar groove absent

36. Maxilla, dorsal infraorbital foramina. Modified from Kimura and Ozawa (2002: 14), Geisler and Sanders (2003: 64), Fitzgerald (2006: 66), Fitzgerald (2010: 38), Churchill et al. (2012: 11).

0=single

1=two

2=three or more

37. Maxilla, foramina in ascending process. New character.

0=foramina absent

1=large dorsally oriented foramen or pair of foramina in ascending process

38. Maxilla, relative position of posterior edge. Modified from Bouetel and Muizon (2006: 14), Fitzgerald (2006: 80), Deméré and Berta (2008: 38), Bisconti et al. (2013: 22), Fordyce and Marx (2013: 35).

0=at level of or posterior to posterior edge of nasal

1=anterior to posterior edge of nasal

39. Maxilla, development of ascending process. Modified from Bisconti (2005: 23).

0=short, approximately as long as wide

1=absent or indistinct, transversely wider than long or not clearly differentiated from rostral portion of maxilla

2=long and narrow, approximately twice as long as wide or longer

40. Rostrum, proximal portion in lateral aspect. Modified from Bisconti (2008: 142), Marx (2011: 2), Fordyce and Marx (2013: 2).

0=below SOS and frontal

1=at or above level of SOS and frontal

41. Rostrum, curvature in lateral aspect. Modified from Bisconti (2005: 62), Bisconti (2008: 139), Marx (2011: 4), Bisconti et al. (2013: 7).

0=regular

1=anterior 1/4 abruptly deflected ventrally

42. Rostrum, width at antorbital notch. Modified from Geisler and Sanders (2003: 7), Deméré et al. (2005: 2), Fitzgerald (2006: 7), Bisconti (2008: 67), Deméré and Berta (2008: 1, 40), Deméré et al. (2008: 2), Fitzgerald (2010: 5), Kimura and Hasegawa (2010: 4), Marx (2011: 6), Churchill et al. (2012: 2), Bisconti et al. (2013: 2), El Adli et al. (2014: 2).

0=wide, 40-54% of BZW

1=narrow, <40% of BZW

2=very wide, >55% of BZW

43. Rostrum, posterior rostral edge in dorsal aspect. Modified from Geisler and Sanders (2003: 11), Fitzgerald (2006: 12), Deméré et al. (2008: 78), Marx (2011: 10), Bisconti et al. (2013: 11), El Adli et al. (2014: 25).

0=straight or gently concave

1=bowed laterally forming V or U shaped antorbital notch

44. Rostrum, maxilla-frontal contact. Modified from Deméré et al. (2008: 17), Fitzgerald (2010: 9), Kimura and Hasegawa (2010: 27), Marx (2011: 13), Bisconti et al. (2013: 29, 31), Fordyce and Marx (2013: 11).

0=maxilla and frontal contact along ankylosed suture

1=frontal-maxilla contact is loose, groove developed

2=contact is loose with large vertical gap or pocket present anteromedially

45. Rostrum, palatal surface. Modified from Geisler and Sanders (2003: 16), Deméré et al. (2005: 39), Bouetel and Muizon (2006: 5), Fitzgerald (2006: 17), Steeman (2007: 23), Bisconti (2008: 25), Deméré and Berta (2008: 41), Deméré et al. (2008: 36), Fitzgerald (2010: 12), Kimura and Hasegawa (2010: 3, 33), Marx (2011: 18), Churchill et al. (2012: 5), Fordyce and Marx (2013: 15, 16), El Adli et al. (2014: 48).

0=flat or gently concave

1=longitudinal keel formed by vomer and maxilla present, gently rounded in cross section and confined to posterior ½ of palate

2=longitudinal keel present, narrow and sharp in cross section and present along at least ¾ of rostrum

46. Rostrum, maxilla/palatine suture in ventral aspect. Modified from Geisler and Sanders (2003: 20), Deméré et al. (2005: 43), Fitzgerald (2006: 21), Deméré et al. (2008: 40), Kimura and Hasegawa (2010: 32), Marx (2011: 21), Churchill et al. (2012: 29), Fordyce and Marx (2011: 20).

0=straight and transverse or anteriorly bowed

1=V-shaped suture with maxillae medially separating palatines

47. Rostrum, anterior edge of narial fossa. Modified from Bisconti (2000: 4), Deméré et al. (2005: 7), Deméré and Berta (2008: 35), Marx (2011: 22).

0=located in posterior 3/4 of rostrum

1=located in anterior 1/4 of rostrum

48. Rostrum, lateral aspect. Modified from Bouetel and Muizon (2006: 2), Steeman (2007: 14), Fitzgerald (2010: 47), Kimura and Hasegawa (2010: 2, 7? ), Marx (2011: 23), Fordyce and Marx (2013: 21), El Adli et al. (2014: 5).

0=step-like profile, depressed anterior to nasals

1=smooth profile

49. Rostrum, shape. Modified from Marx (2011: 24), Bisconti et al. (2013: 2)

0=narrow, width at antorbital notches <80% of rostrum length

1=broad, width >80% of rostrum length

50. Rostrum, geometry of cranial and rostral element contact. Modified from Bisconti (2008: 140), Marx (2011: 52).

0=smooth or angular transition

1=two sets of bones form distinct apex

51. Rostrum, fusion of maxilla-premaxilla suture. Modified from Geisler and Sanders (2003: 10), Deméré et al. (2005: 6), Fitzgerald (2006: 11), Deméré and Berta (2008: 44), Deméré et al. (2008: 5), Fitzgerald (2010: 8), Kimura and Hasegawa (2010: 25), El Adli et al. (2014: 6).

0=firmly ankylosed

1=firmly ankylosed, deep longitudinal groove present

2=not sutured

52. Rostrum, embrasure pits on palate. New character.

0=present

1=absent

53. Rostrum, scalloped edge on maxilla and premaxilla. New character.

0=present

1=absent

54. Vomer, exposure in palate. Modified from Deméré and Berta (2008: 13), Deméré et al. (2008: 96), Fitzgerald (2010: 15), Marx (2011: 19), Fordyce and Marx (2013: 18), El Adli et al. (2014: 55).

0=not exposed

1=exposed in palatal window

55. Vomer, ventral border in ventral view. Modified from Bisconti (2005: 40), Deméré et al. (2005: 44), Deméré et al. (2008: 41), Churchill et al. (2012: 33), Fordyce and Marx (2013: 78), El Adli et al. (2014: 53).

0=posteriormost portion projects beyond posterior tips of palatine and is visible in ventral view

1=completely covered by palatine

56. Lacrimal, dorsal aspect. Modified from Geisler and Sanders (2003: 50), Deméré et al. (2005: 21), Fitzgerald (2006: 51), Bisconti (2008: 165), Deméré and Berta (2008: 12), Deméré et al. (2008: 19), Fitzgerald (2010: 11, 121), Marx (2011: 38), Bisconti et al. (2013: 45), El Adli et al. (2014: 67).

0=entirely lateral to ascending maxilla

1=extends medially between ascending maxilla and rostral portion of maxilla

57. Lacrimal, articulation with maxilla and frontal. New character.

0=sutured to maxilla and frontal

1=unsutured

58. Nasal, lateral edges anterior to preorbital process. Modified from Bouetel and Muizon (2006: 19), Fitzgerald (2010: 52), Kimura and Hasegawa (2010: 38), Bisconti et al. (2013: 41), El Adli et al. (2014: 19).

0=converge anteriorly

1=parallel

2=diverge anteriorly

59. Nasal, length. Modified from Bisconti (2000: 13), Deméré et al. (2005: 8), Deméré et al. (2008: 7), Kimura and Hasegawa (2010: 40), Marx (2011: 54), Churchill et al. (2012: 17), Bisconti et al. (2013: 43), Fordyce and Marx (2013: 36), El Adli et al. (2014: 10).

0=long, >40% BZW

1=short, <40% BZW

2=very long, >65% BZW

60. Nasal, posterior edge. Modified from Kimura and Ozawa (2002: 26), Geisler and Sanders (2003: 122), Deméré et al. (2005: 13), Fitzgerald (2006: 112), Bisconti (2008: 111), Deméré and Berta (2008: 28), Deméré et al. (2008: 12), Fitzgerald (2010: 54), Marx (2011: 55), Bisconti et al. (2013: 57), Fordyce and Marx (2013: 41), El Adli et al. (2014: 17).

0=at level of anterior half of supraorbital process or halfway point

1=at level of posterior half of supraorbital process or postorbital process

2=posterior to postorbital process

3=just anterior to supraorbital process

61. Nasals, dorsal profile of anterior tip. New character.

0=flat

1=anterodorsally flaring

62. Nasals, transverse arching. New character.

0=nasals and premaxillae transversely arched

1=nasals and adjacent rostral elements dorsally flat in cross section

63. Nasal, longitudinal foramina. New character.

0=absent

1=present

64. Nasals, anterior edge. Modified from Fitzgerald (2010: 72), Bisconti et al. (2013: 38), El Adli et al. (2014: 9).

0=in line with p1 or anterior 4/5 of rostrum

1=in line with p2 or posterior 1/5 of rostrum

2=just anterior or at level of anterior edge of supraorbital process

3=in line with anterior half or midpoint of supraorbital process

65. Nasal, posterior portion. Modified from Bisconti et al. (2013: 40), Fordyce and Marx (2013: 37), El Adli et al. (2014: 18).

0=blocky or triangular with medially converging lateral margins

1=transversely thin sheet barely exposed dorsally

66. Nasal, dorsal surface. Modified from Deméré et al. (2005: 12), Deméré et al. (2008: 11), Kimura and Hasegawa (2010: 43), Churchill et al. (2012: 22), Bisconti et al. (2013: 44), Fordyce and Marx (2013: 39), El Adli et al. (2014: 15).

0=flattened

1=forms sagittal keel

67. Nasal, anterior margin. Modified from Deméré et al. (2005: 10), Fitzgerald (2006: 106), Bisconti (2008: 100), Deméré et al. (2008: 9), Kimura and Hasegawa (2010: 42), Churchill et al. (2012: 20), Bisconti et al. (2013: 42), Fordyce and Marx (2013: 38), El Adli et al. (2014: 13).

0=roughly straight or U shape

1=forms posteriorly pointing W shape

2=with point on midline and gap between nasal and premaxilla

68. Orbit, anteroposterior diameter. Modified from Marx (2011: 27).

0= <25% BZW

1= >25% BZW

69. Orbit, dorsal edge in dorsal aspect. Modified from Kimura and Ozawa (2002: 5), Bouetel and Muizon (2006: 30, 34), Deméré and Berta (2008: 6), Kimura and Hasegawa (2010: 52), Marx (2011: 35).

0=roughly straight or slightly concave

1=deeply notched

70. Orbit, dorsal edge relative to lateral edge of rostrum. Modified from Geisler and Sanders (2003: 47), Bouetel and Muizon (2006: 33), Fitzgerald (2006: 48), Kimura and Hasegawa (2010: 53), Marx (2011: 37), Fordyce and Marx (2013: 28).

0=orbit elevated above rostrum edge

1=orbit low, in line with edge or slightly above

2=below rostrum edge

71. Frontal, posterior margin of supraorbital process. Modified from Geisler and Sanders (2003: 60), Bouetel and Muizon (2006: 29), Fitzgerald (2006: 61), Fitzgerald (2010: 35), Kimura and Hasegawa (2010: 50), Marx (2011: 30), Bisconti et al. (2013: 49), Fordyce and Marx (2013: 24).

0=concave

1=straight

72. Frontal, supraorbital process. Modified from Bisconti (2000: 25), Kimura and Ozawa (2002: 3), Geisler and Sanders (2003: 46), Dooley et al. (2004: 29), Bisconti (2005: 20), Deméré et al. (2005: 25), Bouetel and Muizon (2006: 28), Fitzgerald (2006: 47), Steeman (2007: 11), Bisconti (2008: 24), Deméré and Berta (2008: 31), Deméré et al. (2008: 23), Fitzgerald (2010: 32), Kimura and Hasegawa (2010: 49), Marx (2011: 31), Churchill et al. (2012: 24?, 25?, 26), Bisconti et al. (2013: 46), Fordyce and Marx (2013: 25), El Adli et al. (2014: 32).

0=horizontal

1=gradually slope ventrolaterally from vertex

2=abruptly depressed below vertex

73. Frontal, anterior border of supraorbital process in dorsal aspect. Modified from Marx (2011: 32),

0=bordered by lacrimal and maxilla

1=bordered by lacrimal only

74. Frontal, supraorbital process in dorsal aspect. Modified from Deméré et al. (2005: 24), Bouetel and Muizon (2006: 26), Steeman (2007: 10), Bisconti (2008: 108, 152), Deméré et al. (2008: 22), Fitzgerald (2010: 31), Marx (2011: 33), Bisconti et al. (2013: 50).

0=medial portion as long as lateral portion

1=medial portion distinctly shorter, SOPF is triangular

2=medial portion longer than lateral

75. Frontal, anteroposterior length of supraorbital process. Modified from Kimura and Ozawa (2002: 6, 7), Bisconti (2005: 18), Bouetel and Muizon (2006: 27), Steeman (2007: 19), Bisconti (2008: 2), Deméré and Berta (2008: 22), Kimura and Hasegawa (2010: 48), Marx (2011: 34), Bisconti et al. (2013: 47, 48), Fordyce and Marx (2013: 26), El Adli et al. (2014: 29).

0=roughly as long as wide

1=wider than long

2=more than twice as long as wide

76. Frontal, position of line joining anteriormost points of supraorbital process. Modified from Geisler and Sanders (2003: 80), Bouetel and Muizon (2006: 23), Fitzgerald (2006: 83), Steeman (2007: 4), Bisconti (2008: 112), Deméré et al. (2008: 6), Kimura and Hasegawa (2010: 39), Marx (2011: 36), Fordyce and Marx (2013: 27).

0=passing through nasals

1=posterior to or at posterior edge of nasals

2=at anterior edge of nasals

3=anterior to nasals

77. Frontal, anteromedial corner of supraorbital process. Modified from Marx (2011: 41), Fordyce and Marx (2013: 32).

0=lacks anterior projection

1=triangular projection present

78. Frontal, maxilla/frontal overlap. Modified from Kimura and Ozawa (2002: 1), Geisler and Sanders (2003: 12, 76, 127), Dooley et al. (2004: 37), Deméré et al. (2005: 18), Fitzgerald (2006: 13, 78, 117), Deméré and Berta (2008: 3), Deméré et al. (2008: 17), Kimura and Hasegawa (2010: 23), Marx (2011: 44), Churchill et al. (2012: 15), Fordyce and Marx (2013: 42).

0=maxilla overlaps anteromedial corner of supraorbital process of frontal

1=maxilla almost completely overlaps frontal

79. Frontal, orbitotemporal crest. Modified from Bisconti (2000: 34), Geisler and Sanders (2003: 131), Bisconti (2005: 3, 57), Bouetel and Muizon (2006: 24), Fitzgerald (2006: 121), Steeman (2007: 19), Bisconti (2008: 3, 19, 90), Deméré and Berta (2008: 43), Deméré et al. (2008: 79), Kimura and Hasegawa (2010: 46), Marx (2011: 56), Churchill et al. (2012: 23), Bisconti et al. (2013: 51, 52), Fordyce and Marx (2013: 43), El Adli et al. (2014: 33).

0=forms posterior margin of supraorbital process

1=anteriorly retracted from posterior margin, within posterior 50% of SOPF

2=positioned within anterior 50% of SOPF

3=indistinct

80. Frontal, orbitotemporal crest, shape. Modified from Bisconti (2008: 151), Marx (2011: 57), Bisconti et al. (2013: 52), Fordyce and Marx (2013: 44).

0=straight

1=distal half abruptly projecting posterolaterally

81. Frontal, supraorbital process. New character.

0=foramina absent

1=numerous foramina and sulci present on dorsal surface

82. Frontal, triangular extension of frontal anteriorly separating nasals. Modified from Geisler and Sanders (2003: 121), Deméré et al. (2005: 11), Bouetel and Muizon (2006: 20), Fitzgerald (2006: 111), Deméré et al. (2008: 10), Fitzgerald (2010: 53), Kimura and Hasegawa (2010: 45), Churchill et al. (2012: 21), Fordyce and Marx (2013: 40), El Adli et al. (2014: 14).

0=present

1=absent

83. Frontal, preorbital process. Modified from Fitzgerald (2010: 33), El Adli et al. (2014: 30).

0=anterior edge of preorbital process has squared off or slightly concave margin

1=rounded off, anteriorly convex margin

84. Frontal, postorbital ridge. Modified from Geisler and Sanders (2003: 62), Fitzgerald (2006: 64), Fitzgerald (2010: 36).

0=present, forms well defined ridge posterior to optic canal

1=no well defined ridge, smoothly convex surface

85. Frontal, position of optic groove. Modified from Bisconti et al. (2013: 115).

0=positioned about anteromedial midpoint of supraorbital process

1=positioned in posterior 1/3 of supraorbital process

86. Frontal, height relative to nasals. Modified from Geisler and Sanders (2003: 124), Bisconti (2005: 19), Fitzgerald (2006: 114), Fitzgerald (2010: 56), Churchill et al. (2012: 25).

0=higher than nasals

1=same height as nasals

87. Frontal, orbitotemporal crest roofs over entire temporal fossa. Modified from Fitzgerald (2006: 122), Fitzgerald (2010: 58).

0=absent

1=present

88. Cranium, shape of temporal fossa. Modified from Bisconti (2000: 30, 31), Bouetel and Muizon (2006: 32), Steeman (2007: 12), Kimura and Hasegawa (2010: 77), Marx (2011: 58), Fordyce and Marx (2013: 45).

0=longer than wide, or roughly equidimensional

1=wider than long

89. Parietal, intertemporal constriction. Modified from Bisconti (2000: 32), Bisconti (2005: 31), Fitzgerald (2006: 127), Bisconti (2008: 126), Deméré and Berta (2008: 8), Kimura and Hasegawa (2010: 59, 60), Marx (2011: 59), Bisconti et al. (2013: 63, 69, 70), Fordyce and Marx (2013: 46), El Adli et al. (2014: 31).

0=longer anteroposteriorly than transversely wide, straight medial margins of temporal fossae

1=longer anteroposteriorly than transversely wide, concave medial margins of temporal fossae

2=wider transversely than long

90. Parietal and interparietal. Modified from Bisconti (2008: 22), Deméré and Berta (2008: 4), Deméré et al. (2008: 95), Marx (2010: 61), Bisconti et al. (2013: 77), Fordyce and Marx (2013: 47).

0=anteriormost point behind supraorbital process of frontal or at level of postorbital process

1=anteriormost point in line with supraorbital process

91. Parietal, dorsal aspect. Modified from Bisconti (2000: 41), Geisler and Sanders (2003: 134), Deméré et al. (2005: 27), Bouetel and Muizon (2006: 35), Fitzgerald (2006: 124), Bisconti (2008: 34, 74), Deméré et al. (2008: 25), Fitzgerald (2010: 59), Kimura and Hasegawa (2010: 55, 59), Marx (2011: 62), Churchill et al. (2012: 36), Bisconti et al. (2014: 78), El Adli et al. (2014: 34).

0=contact medially or separated by interparietal

1=present in skull roof as small triangular exposures in intertemporal constriction

2=completely absent in skull roof

92. Parietal and Interparietal, anteriormost point. Modified from Kimura and Ozawa (2002: 28), Geisler and Sanders (2003: 133), Deméré et al. (2005: 22), Fitzgerald (2006: 123), Bisconti (2008: 163), Marx (2011: 63), Deméré et al. (2008: 20), Kimura and Hasegawa (2010: 21), Bisconti et al. (2013: 56, 76), Fordyce and Marx (2013: 49), El Adli et al. (2014: 27).

0=posterior to or at position of posteriormost ascending maxilla

1=more anterior

93. Parietal, intertemporal constriction. Modified from Bisconti (2000: 33), Fitzgerald (2006: 126), Bisconti (2008: 79), Bisconti et al. (2013: 64).

0=high sagittal crest present

1=sagittal crest absent or present as low ridge

94. Parietal, orbitotemporal crest. New character.

0=extends posteriorly onto parietal

1=on frontals only

95. Alisphenoid, contacts in temporal fossa. Modified from Geisler and Sanders (2003: 141), Fitzgerald (2006: 131), Bisconti (2008: 113, 114), Marx (2011: 66), Churchill et al. (2012: 32), Bisconti et al. (2013: 97) Fordyce and Marx (2013: 53).

0=contacts frontal, squamosal, parietal, palatine, and pterygoid

1=contacts frontal, squamosal, parietal, pterygoid, not palatine

2=contacts squamosal, parietal, pterygoid, not frontal or palatine

3=contacts parietal and pterygoid, not squamosal, palatine, or frontal

4=no alisphenoid exposed

96. Alisphenoid, exposure. Modified from Fitzgerald (2006: 132), Bisconti (2008: 114), Bisconti et al. (2013: 96).

0=small or not exposed

1=hypertrophied

97. Braincase, frontoparietal suture. Modified from Fitzgerald (2010: 60), Bisconti et al. (2013: 55), El Adli et al. (2014: 36).

0=transversely oriented

1=suture is strongly V or U-shaped, with sutures converging posteriorly and frontal penetrating between parietals

98. Braincase, posterior apex of nuchal crest. Modified from Bisconti (2008: 83), Marx (2011: 65), Bisconti et al. (2013: 73) Fordyce and Marx (2013: 51).

0=anterior to or at level of posteriormost point of occipital condyles

1=posterior to condyles

99. Braincase, postparietal foramen. Modified from Bisconti et al. (2013: 71).

0=absent

1=present

100. Braincase, shape of parieto-squamosal suture. Modified from Bisconti (2005: 4?), Bouetel and Muizon (2006: 37), Bisconti (2008: 4?), Churchill et al. (2012: 38), Bisconti et al. (2013: 74), Fordyce and Marx (2013: 59).

0=ridge absent

1=present and low

2=present and distinctly elevated

101. Braincase, squamosal-alisphenoid suture. Modified from Geisler and Sanders (2003: 180), Fitzgerald (2006: 170), Bisconti (2008: 115), Fitzgerald (2010: 75).

0=anterior to external foramen ovale

1=courses along branch of trigeminal nerve

2=just medial to anterior edge of squamosal fossa

102. Braincase, tubercle on parieto-squamosal suture. Modified from Fordyce and Marx (2013: 52).

0=absent

1=tubercle developed at junction of suture and occipital shield

103. Occipital, anteriormost point of supraoccipital. Modified from Bisconti (2000: 42), Kimura and Ozawa (2002: 2), Geisler and Sanders (2003: 139), Deméré et al. (2005: 29), Bouetel and Muizon (2006: 39), Fitzgerald (2006: 129), Steeman (2007: 5), Bisconti (2008: 78), Deméré et al. (2008: 27), Fitzgerald (2010: 62), Kimura and Hasegawa (2010: 78), Marx (2011: 64), Churchill et al. (2012: 49), Bisconti et al. (2013: 56, 98), Fordyce and Marx (2013: 50), El Adli et al. (2014: 37).

0=at level of braincase between subtemporal crest and posteriormost skull

1=at level of gap between postorbital process and subtemporal crest

2=at level of posterior half of supraorbital process or postorbital process

3=at level of anterior half of supraorbital process

4=anterior to or at level of anterior edge of supraorbital process

104. Occipital, paired tubercles on occipital shield. Modified from Bisconti (2000: 51), Marx (2011: 76).

0=absent

1=present

105. Occipital, lateral margins of occipital shield in dorsal aspect. Modified from Deméré et al. (2005: 31), Bisconti (2008: 62), Deméré et al. (2008: 29), Kimura and Hasegawa (2010: 81), Marx (2011: 77), Churchill et al. (2012: 50), Bisconti et al. (2013: 107, 109), Fordyce and Marx (2013: 69), El Adli et al. (2014: 39).

0=convex

1=straight or concave

2=sinuous

106. Occipital, paroccipital process in dorsal aspect. Modified from Bisconti (2000: 55), Kimura and Ozawa (2002: 29), Geisler and Sanders (2003: 197), Bisconti (2005: 28), Deméré et al. (2005: 46), Bouetel and Muizon (2006: 55), Fitzgerald (2006: 187), Deméré et al. (2008: 43), Fitzgerald (2010: 78), Kimura and Hasegawa (2010: 83), Marx (2011: 83), Churchill et al. (2012: 35), Bisconti et al. (2013: 110), Fordyce and Marx (2013: 81), El Adli et al. (2014: 60).

0=extends anterior to posterior edge of condyles

1=at level of or posterior to posterior edge of condyles

107. Occipital, nuchal crests in dorsal view. Modified from Bisconti (2000: 40, 47), Geisler and Sanders (2003: 153, 154), Bouetel and Muizon (2006: 54), Fitzgerald (2006: 143, 144), Bisconti (2008: 82, 132), Deméré and Berta (2008: 34), Fitzgerald (2010: 69), Kimura and Hasegawa (2010: 80), Churchill et al. (2012: 55), Bisconti et al. (2013: 67, 68).

0=vertical and posteriorly or dorsally directed, not obscuring temporal fossa

1=subvertical to horizontal and anterolaterally directed, obscuring temporal wall but not temporal fossa

2=subvertical to horizontal and anterolaterally directed, obscuring temporal fossa

108. Occipital, paroccipital process. New character.

0=forms a vertical, plate-like process

1=anteroposteriorly swollen in ventral aspect

2=anteroposteriorly swollen with anteroventral pit for stylohyoid

109. Occipital, shape in posterior view. Modified from Bisconti (2005: 69).

0=trefoil shaped

1=triangular or subtriangular

110. Supraoccipital, shape of apex. Modified from Bisconti (2000: 48), Geisler and Sanders (2003: 152), Bisconti (2005: 60), Deméré et al. (2005: 30), Bouetel and Muizon (2006: 53), Fitzgerald (2006: 142), Bisconti (2008: 63), Deméré et al. (2008: 28), Fitzgerald (2010: 68), Kimura and Hasegawa (2010: 79), Churchill et al. (2012: 51), Bisconti et al. (2013: 105, 106), El Adli et al. (2014: 38).

0=rounded or triangular

1=flattened apex

107. Cranium, dorsally highest part. Modified from Geisler and Sanders (2003: 128), Bouetel and Muizon (2006: 38), Fitzgerald (2006: 118), Bisconti (2008: 164).

0=apex of occipital shield

1=nuchal crest elevated above occipital apex

2=rostral elements elevated above occipital

111. Occipital, external occipital crest. Modified from Bisconti (2000: 49, 50), Geisler and Sanders (2003: 155), Bisconti (2005: 22, 68), Bouetel and Muizon (2006: 40), Fitzgerald (2006: 145), Bisconti (2008: 145?, 157), Fitzgerald (2010: 70), Kimura and Hasegawa (2010: 82), Churchill et al. (2012: 52, 54), Bisconti et al. (2013: 103), Fordyce and Marx (2013: 70).

0=sharp ridge present dividing shield into two concave fossae

1=blunt ridge dividing anterior half of shield into two dorsolaterally sloping facets

2=absent

112. Occipital, dorsal condyloid fossae. Modified from Geisler and Sanders (2003: 156), Fitzgerald (2006: 146), Fitzgerald (2010: 71), Kimura and Hasegawa (2010: 85).

0=present

1=absent

114. Occipital, posterior margin of exoccipital. New character.

0=straight

1=posteriorly convex and bulbous

115. Squamosal, zygomatic process. Modified from Geisler and Sanders (2003: 142), Deméré et al. (2005: 32), Bouetel and Muizon (2006: 42), Fitzgerald (2006: 132), Bisconti (2008: 91), Deméré et al. (2008: 30), Fitzgerald (2010: 63), Kimura and Hasegawa (2010: 64), Marx (2011: 67), Churchill et al. (2012: 41, 45), Bisconti et al. (2013:92), Fordyce and Marx (2013: 54), El Adli et al. (2014: 40).

0=directed anteromedially

1=directed anteriorly

2=directed anterolaterally

116. Squamosal, zygomatic process in lateral aspect. Modified from Deméré and Berta (2008: 10), Fitzgerald (2010: 64), Marx (2011: 68), Fordyce and Marx (2013: 55), El Adli et al. (2014: 45).

0=dorsal and ventral margins convergent

1=zygomatic expanded anteriorly and posteriorly with constriction in middle

117. Squamosal, zygomatic process. Modified from Kimura and Ozawa (2002: 21), Deméré et al. (2005: 23), Bouetel and Muizon (2006: 31), Bisconti (2008: 93), Deméré and Berta (2008: 11), Deméré et al. (2008: 21), Fitzgerald (2010: 65), Kimura and Hasegawa (2010: 62), Marx (2011: 69), Churchill et al. (2012: 46), Bisconti et al. (2013: 54), Fordyce and Marx (2013: 56), El Adli et al. (2014: 28).

0=does not contact postorbital process, entirely posterior to frontal

1=does not contact postorbital process, anterior tip is lateral to frontal

2=contacts or situated below postorbital process

118. Squamosal, supramastoid crest. Modified from Bisconti (2000: 39), Bisconti (2005: 14), Bouetel and Muizon (2006: 43), Fitzgerald (2006: 139), Bisconti (2008: 18, 134?, 158), Kimura and Hasegawa (2010: 65), Marx (2011: 70), Churchill et al. (2012: 40), Bisconti et al. (2013: 87, 88), Fordyce and Marx (2013: 57).

0=present along length of zygomatic

1=extends past posterior margin of temporal fossa but not to tip of zygomatic

2=does not extend anterior to posterior margin of temporal fossa

119. Squamosal, axis of development. Modified from Bisconti (2000: 68), Kimura and Ozawa (2002: 19), Bisconti (2005: 33), Deméré et al. (2005: 35), Bisconti (2008: 35), Deméré et al. (2008: 33), Marx (2011: 71), Churchill et al. (2012: 42), Bisconti et al. (2013: 86), Fordyce and Marx (2013: 58), El Adli et al. (2014: 43).

0=anteroposterior

1=dorsoventral

120. Squamosal, transverse width lateral to exoccipital (posterior aspect). Modified from Geisler and Sanders (2003: 145), Deméré et al. (2005: 36), Fitzgerald (2006: 135), Deméré et al. (2008: 34), Fitzgerald (2010: 66), Kimura and Hasegawa (2010: 73), Marx (2011: 72), Fordyce and Marx (2013: 61), El Adli et al. (2014: 44).

0=intermediate width, 10-40% of distance from midline to lateral edge of exoccipital

1=wide, 35-49% of distance

2=very wide, >50% of distance

3=narrow, <10% of distance

121. Squamosal, orientation of postglenoid process in lateral aspect. Modified from Bouetel and Muizon (2006: 45), Marx (2011: 73), Bisconti et al. (2013: 94), Fordyce and Marx (2013: 63).

0=subvertical to anteriorly projecting

1=subvertical to posteroventrally projecting

2=concave posterior margin, horizontal and posteriorly directed postglenoid process

122. Squamosal, orientation of postglenoid process in ventral view. Modified from Bouetel and Muizon (2006: 46), Kimura and Hasegawa (2010: 67), Marx (2011: 74), Fordyce and Marx (2013: 65), El Adli et al. (2014: 46).

0=ventrally oriented

1=ventrolaterally oriented

123. Squamosal, squamosal cleft. Modified from Bisconti (2005: 26), Deméré et al. (2005: 34), Bisconti (2008: 69), Deméré et al. (2008: 32), Kimura and Hasegawa (2010: 70), Marx (2011: 75), Churchill et al. (2012: 43), Bisconti et al. (2013: 80), Fordyce and Marx (2013: 68), El Adli et al. (2014: 42).

0=absent

1=present

124. Squamosal, twisting of postglenoid process in ventral aspect. Modified from Bouetel and Muizon (2006: 47), Kimura and Hasegawa (2010: 68), Marx (2011: 78), Fordyce and Marx (2013: 67).

0=not twisted, oriented transversely

1=twisted clockwise on left and counterclockwise on right

125. Squamosal, zygomatic shape in dorsal aspect. Modified from Bouetel and Muizon (2006: 41).

0=zygomatic tapers anteriorly

1=medial and lateral margins parallel

126. Squamosal, subtemporal crest. Modified from Deméré and Berta (2008: 33).

0=well developed horizontal crest that is anterolaterally convex or bulges into temporal fossa

1=well developed anterolaterally concave horizontal crest

2=crest absent, squamosal is anteroventrally smooth and convex

127. Squamosal, secondary squamosal fossa. Modified from Bisconti (2008: 162).

0=absent

1=developed as a pit or trough lateral to squamosal fossa

128. Squamosal, glenoid fossa. Modified from Bisconti (2000: 23), Kimura and Ozawa (2002: 22), Bisconti (2005: 15, 55), Bouetel and Muizon (2006: 49), Bisconti (2008: 20, 36), Kimura and Hasegawa (2010: 72), Bisconti et al. (2013: 84).

0=margins of fossa clearly developed

1=absent, margins of fossa indistinct or convex

129. Squamosal, squamosal prominence on supramastoid crest. Modified from Geisler and Sanders (2003: 149), Fitzgerald (2006: 139), Fordyce and Marx (2013: 60).

0=absent

1=developed as large knob or tubercle

2=developed as a dorsally arched, narrow flange

130. Squamosal, ventromedial fossa on zygomatic process. New character.

0=fossa absent

1=ventral or ventromedial fossa present at apex

2=longitudinal groove for masseter insertion present on ventral surface of zygomatic base

131. Squamosal, longitudinal twisting of zygomatic process. Modified from Bisconti et al. (2013: 93).

0=absent

1=zygomatic process twisted so that lateral surface faces dorsolaterally

132. Squamosal, curvature of zygomatic process in dorsal view. Modified from Bisconti (2000: 38).

0=lateral margin is straight or laterally convex, concave medial margin

1=medially bowed with laterally concave medial and lateral margins

133. Squamosal, postglenoid process in lateral view. Modified from Bouetel and Muizon (2006: 48), Fitzgerald (2006: 141), Fitzgerald (2010: 67), Kimura and Hasegawa (2010: 66), Churchill et al. (2012: 48).

0=anteroposteriorly flattened

1=anteroposteriorly inflated

134. Squamosal, squamosal crease. Modified from Bisconti (2008: 149).

0=absent

1=present

135. Squamosal, proportions of zygomatic process in lateral view. Modified from Bisconti (2000: 37), Geisler and Sanders (2003: 188), Bisconti (2005: 27, 38), Bouetel and Muizon (2006: 41), Fitzgerald (2006: 178), Bisconti (2008: 37), Kimura and Hasegawa (2010: 63), Churchill et al. (2012: 44), Bisconti et al. (2013: 89).

0=anteroposterior length is twice as long as dorsoventral depth at base of process or longer

1=dorsoventral depth and anteroposterior length are approximately equal

2=dorsoventrally deeper than long

136. Squamosal, dorsal arching in lateral view. Modified from Bisconti (2013: 90).

0=absent

1=present

137. Squamosal, tympanosquamosal recess. Modified from Geisler and Sanders (2003: 178, 260), Fitzgerald (2006: 168, 250), Fitzgerald (2010: 74).

0=absent, but small pit for sigmoid process is present

1=absent

2=present

138. Squamosal, shape of lateral skull border. Modified from Fordyce and Marx (2013: 42).

0=lateral skull border forms an angle

1=continuous lateral skull border

139. Squamosal, length of squamosal fossa. Modified from Geisler and Sanders (2003: 146, 147, 148), Deméré et al. (2005: 33), Fitzgerald (2006: 136, 137, 138), Deméré and Berta (2008: 17), Deméré et al. (2008: 31), Kimura and Hasegawa (2010: 74, 75), Fordyce and Marx (2013: 62), El Adli et al. (2014: 41).

0=length of fossa equal to or exceeds width of temporal fossa

1=length of fossa is 3/4 of temporal fossa width or longer

2=length of squamosal fossa is under 3/4 of temporal fossa width

140. Squamosal, ventral extension of postglenoid process. Modified from Bisconti (2008: 120, 133), Fordyce and Marx (2013: 64).

0=ventral edge of postglenoid process approximately in line with or dorsal to ventral edge of exoccipital

1=extends ventral to ventral edge of exoccipital

141. Squamosal, outline of postglenoid process in anterior or posterior view. Modified from Geisler and Sanders (2003: 151), Fordyce and Marx (2013: 66).

0=parabolic

1=parabolic but with parallel or concave lateral and medial edges

2=triangular

3=trapezoidal with ventrally deflected medial border

142. Squamosal, base of postglenoid process in ventral view. Modified from Steeman (2007: 19), Bisconti (2008: 76?), Kimura and Hasegawa (2010: 69), Bisconti et al. (2013: 111), Fordyce and Marx (2013: 77).

0= in line with center of tympanic bulla

1= in line with anterior half of tympanic bulla or further anterior

2= posterior to the longitudinal midpoint of the tympanic bulla

143. Pterygoid, ventral aspect. Modified from Kimura and Ozawa (2002: 18), Bisconti (2005: 48, 50), Deméré et al. (2005: 42), Steeman (2007: 22), Bisconti (2008: 46), Deméré and Berta (2008: 15), Deméré et al. (2008: 39), Kimura and Hasegawa (2010: 31), Marx (2011: 79), Churchill et al. (2012: 30), Bisconti et al. (2013: 117), Fordyce and Marx (2013: 71), El Adli et al. (2014: 52).

0=exposed for entire or most of their length

1=anterior half covered by palatines

2=almost entirely covered by palatines

144. Pterygoid, shape of hamulus. Modified from Geisler and Sanders (2003: 172), Fitzgerald (2006: 162), Bisconti (2008: 150), Deméré et al. (2008: 80), Kimura and Hasegawa (2010: 35), Churchill et al. (2012: 31), Fordyce and Marx (2013: 74), El Adli et al. (2014: 58).

0=fingerlike

1=expanded into dorsoventrally flattened plate partially flooring pterygoid sinus fossa

2=reduced in size or almost absent

145. Braincase, foramen pseudovale. Modified from Bisconti (2000: 59, 60), Bisconti (2005: 8), Deméré et al. (2005: 38), Bouetel and Muizon (2006: 52), Bisconti (2008: 11, 117), Deméré et al. (2008: 35), Kimura and Hasegawa (2010: 76), Marx (2011: 80), Bisconti et al. (2013: 121), Fordyce and Marx (2013: 75), El Adli et al. (2014: 47).

0=absent

1=within pterygoid

2=between squamosal and pterygoid

146. Basioccipital, basioccipital crest. Modified from Bisconti (2000: 65), Geisler and Sanders (2003: 191), Deméré et al. (2005: 45), Bouetel and Muizon (2006: 50, 51), Fitzgerald (2006: 181), Steeman (2007: 21), Bisconti (2008: 8), Deméré and Berta (2008: 42), Deméré et al. (2008: 42), Fitzgerald (2010: 76), Kimura and Hasegawa (2010: 84), Marx (2011: 81), Churchill et al. (2012: 34), Fordyce and Marx (2013: 79), El Adli et al. (2014: 59).

0= transversely narrow

1= wide and bulbous

147. Basioccipital, basioccipital crest orientation. Modified from Geisler and Sanders (2003: 194), Fitzgerald (2006: 184), Deméré and Berta (2008: 18), Fitzgerald (2010: 77), Marx (2011: 82), Bisconti (2008: 194), Marx (2011: 82), Fordyce and Marx (2013: 80),

0= diverging posteriorly

1= parallel or subparallel

148. Basioccipital, width relative to basisphenoid. New character.

0=basisphenoid abruptly narrower than basioccipital, <2/3 basioccipital width

1=basisphenoid and basioccipital roughly equivalent, >2/3 basioccipital width

149. Periotic, cerebral elongation of pars cochlearis. Modified from Geisler and Luo (1996: 12), Kimura and Ozawa (2002: 61), Geisler and Sanders (2003: 230), Dooley et al. (2004: 12), Bisconti (2005: 81), Deméré et al. (2005: 50), Bouetel and Muizon (2006: 77), Fitzgerald (2006: 220), Steeman (2007: 75), Deméré and Berta (2008: 46), Deméré et al. (2008: 46), Fitzgerald (2010: 98), Kimura and Hasegawa (2010: 92, 94?), Ekdale et al. (2011: 27), Marx (2011: 84), Churchill et al. (2012: 69), Bisconti et al. (2013: 134), Fordyce and Marx (2013: 84), El Adli et al. (2014: 86).

0=absent

1=present, pars cochlearis dorsoventrally longer than anteroposteriorly wide

150. Periotic, contact of anterior process with tympanic bulla. Modified from Geisler and Luo (1996: 3, 5, 6, 27), Kimura and Ozawa (2002: 53, 55), Geisler and Sanders (2003: 209, 253, 255), Dooley et al. (2004: 3, 6), Fitzgerald (2006: 199, 243, 245), Bisconti (2008: 29), Fitzgerald (2010: 114), Marx (2011: 85), Churchill et al. (2012: 63), Fordyce and Marx (2013: 91).

0=little or no contact, or contact with accessory ossicle

1=fused into anterior pedicle

151. Periotic, lateral tuberosity. Modified from Geisler and Luo (1996: 4, 11), Kimura and Ozawa (2002: 54, 60?), Geisler and Sanders (2003: 210, 211), Dooley et al. (2004: 4, 11?), Bisconti (2005: 53), Deméré et al. (2005: 51), Bouetel and Muizon (2006: 69), Fitzgerald (2006: 200, 201), Steeman (2007: 28), Bisconti (2008: 55), Deméré et al. (2008: 47), Fitzgerald (2010: 85), Kimura and Hasegawa (2010: 89), Ekdale et al. (2011: 18), Marx (2011: 86), Churchill et al. (2012: 65), Bisconti et al. (2013: 152, 154), Fordyce and Marx (2013: 92), El Adli et al. (2014: 78).

0=absent or blunt projection

1=conical and laterally projecting

2=long, ventrally directed blade-like projection half the length of the anterior process or longer

3=flattened triangular shelf, anteroposteriorly longer than wide

152. Periotic, hiatus epitympanicus. Modified from Geisler and Luo (1996: 8), Kimura and Ozawa (2002: 57), Dooley et al. (2004: 8), Marx (2011: 87).

0=present

1=absent or indistinct

153. Periotic, facial canal, internal acoustic meatus and perilymphatic foramen aligned. Modified from Geisler and Luo (1996: 20), Steeman (2007: 39), Marx (2011: 88).

0=absent

1=present

154. Periotic, aperture for cochlear aqueduct. Modified from Geisler and Sanders (2003: 227), Marx (2011: 89), Fordyce and Marx (2013: 102).

0=smaller than aperture for vestibular aqueduct

1=approximately similar in size

155. Periotic, facial canal. Modified from Kimura and Ozawa (2002: 69), Geisler and Sanders (2003: 238), Dooley et al. (2004: 20), Deméré et al. (2005: 56), Bouetel and Muizon (2006: 75), Fitzgerald (2006: 228), Deméré et al. (2008: 52), Fitzgerald (2010: 105), Kimura and Hasegawa (2010: 105), Ekdale et al. (2011: 47), Marx (2011: 90), Bisconti et al. (2013: 131, 132), Fordyce and Marx (2013: 105).

0=roughly circular and contiguous with broad hiatus fallopii

1=continuous with fissure-like hiatus fallopii

156. Periotic, articulation with tympanic bulla. Modified from Kimura and Ozawa (2002: 76), Geisler and Sanders (2003: 241), Dooley et al. (2004: 27), Bouetel and Muizon (2006: 62), Fitzgerald (2006: 231), Steeman (2007: 37), Bisconti (2008: 28), Fitzgerald (2010: 107), Marx (2011: 91), Churchill et al. (2012: 66), Fordyce and Marx (2013: 107).

0=unfused

1=fused in adults

157. Periotic, facial sulcus distal to stylomastoid notch. Modified from Geisler and Luo (1996: 26), Kimura and Ozawa (2002: 75), Geisler and Sanders (2003: 244), Dooley et al. (2004: 26), Bouetel and Muizon (2006: 83), Fitzgerald (2006: 234), Bisconti (2008: 105), Fitzgerald (2010: 108), Kimura and Hasegawa (2010: 111), Marx (2011: 92), Bisconti et al. (2013: 158), Fordyce and Marx (2013: 108).

0=short, no sulcus posterior to notch

1=long but relatively shallow sulcus, disappearing distally

158. Periotic, morphology of compound posterior process. Modified from Geisler and Luo (1996: 23, 28), Kimura and Ozawa (2002: 72, 77), Geisler and Sanders (2003: 245, 248, 249), Dooley et al. (2004: 23, 28), Bouetel and Muizon (2006: 84, 85), Fitzgerald (2006: 239, 235, 238), Steeman (2007: 35), Bisconti (2008: 23), Fitzgerald (2010: 109, 112), Kimura and Hasegawa (2010: 107, 112, 113), Marx (2011: 93), Bisconti et al. (2013: 155, 156), Fordyce and Marx (2013: 110, 111), El Adli et al. (2014: 103).

0=short, processes not exposed or barely exposed on skull wall

1=conical-tetrahedral process exposed laterally, widening distally

2=process exposed and cylindrical or flattened

159. Periotic, neck of posterior process. Modified from Geisler and Luo (1996: 24), Kimura and Ozawa (2002: 73), Geisler and Sanders (2003: 247), Dooley et al. (2004: 24), Bouetel and Muizon (2006: 56), Fitzgerald (2006: 237), Steeman (2007: 36), Kimura and Hasegawa (2010: 109), Marx (2011: 94), Churchill et al. (2012: 67).

0=transversely constricted

1=transversely and dorsoventrally constricted

2=absent

160. Periotic, apex of anterior process. Modified from Geisler and Sanders (2003: 208), Fitzgerald (2006: 198), Bisconti (2008: 82, 127), Kimura and Hasegawa (2010: 88), Ekdale et al. (2011: 20), Bisconti et al. (2013: 147), El Adli et al. (2014: 80).

0=transversely blunt or massive

1=conical or sharply bladelike

161. Periotic, superior process. Modified from Geisler and Luo (1996: 17), Geisler and Sanders (2003: 231?, 232, 233), Fitzgerald (2006: 221?, 222, 223), Bisconti (2008: 66, 75?), Fordyce and Marx (2013: 103), El Adli et al. (2014: 84, 91).

0=continuous, well developed

1=discontinuous with anterior and posterior apices

2=discontinuous, posterior apex only, anterior apex missing

3=absent or poorly developed

162. Periotic, conical projection on dorsal surface posteriorly adjacent to IAM. Modified from Geisler and Sanders (2003: 235), Fitzgerald (2006: 225), Fitzgerald (2010: 103).

0=absent

1=present

163. Periotic, vestibular and cochlear nerve canals. Modified from Geisler and Sanders (2003: 236), Fitzgerald (2006: 226), Fitzgerald (2010: 101, 104).

0=separated by crest, crest is dorsally higher than crista transversa

1=separated by crest

2=confluent, or separated by crest deeply recessed within internal acoustic meatus

164. Periotic, posterior bullar facet. Modified from Geisler and Sanders (2003: 242), Fitzgerald (2006: 232).

0=flat

1=transversely concave

2=transversely convex

165. Periotic, pars cochlearis. Modified from Geisler and Sanders (2003: 221), Fitzgerald (2006: 211), Fitzgerald (2010: 92), Ekdale et al. (2011: 30), El Adli et al. (2014: 102).

0=longitudinal ridge present on ventral surface

1=ridge absent, pars cochlearis smooth and convex

166. Periotic, anterior process. Modified from Geisler and Luo (1996: 2), Kimura and Ozawa (2002: 52), Dooley et al. (2004: 2).

0=Deep pit present on lateral surface

1=pit shallow or absent

167. Periotic, position of fenestra ovalis. New character.

0=present ~2/3 of a/p distance from anterior margin of pars cochlearis

1=present within anterior 2/3 of pars cochlearis

168. Periotic, incisural flange appressed to anterior pars cochlearis. New character.

0=absent

1=present

169. Periotic, posterior bullar facet divided into two distinct facets divided by transverse hingeline. Modified from Geisler and Sanders (2003: 243), Fitzgerald (2006: 233).

0=absent

1=present

2=posterior processes fused

170. Periotic, position of mallear fossa relative to lateral tuberosity. New character.

0=posterior to lateral tuberosity

1=medial to lateral tuberosity

171. Periotic, orientation of anterolateral sulcus. Modified from Fitzgerald (2010: 82).

0=transverse

1=anterolaterally

172. Periotic, groove on dorsal side of posterior process. Modified from Geisler and Sanders (2003: 239), Fitzgerald (2006: 229).

0=absent

1=groove on dorsomedial side of posterior process

2=grooves present on dorsomedial and dorsolateral sides of posterior process

3=deep dorsolateral groove, poorly developed dorsomedial groove

173. Periotic, longitudinal grooves on posterior bullar facet. New character.

0=absent

1=present

174. Periotic, anteromedial margin of pars cochlearis. Modified from Geisler and Sanders (2003: 219), Fitzgerald (2006: 209), Fitzgerald (2010: 90), Kimura and Hasegawa (2010: 91), Fordyce and Marx (2013: 94).

0=forms corner

1=smoothly convex

175. Periotic, posteroexternal foramen. New character.

0=small pore

1=foramen opens into anteroposteriorly directed fissure or furrow

176. Periotic, ventral margin of anterior process in medial view. Modified from Geisler and Sanders (2003: 202), Bouetel and Muizon (2006: 64), Fitzgerald (2006: 129), Fitzgerald (2010: 80).

0=at same level or dorsal to ventral edge of pars cochlearis

1=well ventral to ventral edge of pars cochlearis, process is ventrally deflected

177. Periotic, length of anterior process. Modified from Geisler and Luo (1996: 1), Kimura and Ozawa (2002: 51), Geisler and Sanders (2003: 203), Dooley et al. (2004: 1), Bouetel and Muizon (2006: 63), Fitzgerald (2006: 193), Fitzgerald (2010: 81), Kimura and Hasegawa (2010: 86), Ekdale et al. (2011: 37), Bisconti et al. (2013: 146), Fordyce and Marx (2013: 87), El Adli et al. (2014: 95).

0=present but very short, length <36% of the length of the pars cochlearis

1=short, length between 59 and 94% of pars cochlearis length.

2=approximately same length as pars cochlearis, 100-134%

3=long, length between 141% and 174% of pars cochlearis length

4=very long, >212% of pars cochlearis length

178. Periotic, angle formed by dorsal and posterior margins. Modified from Geisler and Sanders (2003: 246), Fitzgerald (2006: 236), Fitzgerald (2010: 110), Fordyce and Marx (2013: 109).

0=broadly rounded or forms corner >90º

1=forms corner ≤ 90º

179. Periotic, shape of dorsal margin of anterior process (between anteroventral and anterodorsal angles). New character.

0=straight or dorsally convex

1=dorsally concave

180. Periotic, position of dorsoventrally deepest part of anterior process. Modified from Geisler and Sanders (2003: 201), Bisconti (2005: 30), Bouetel and Muizon (2006: 70), Fitzgerald (2006: 191), Bisconti (2008: 84), Bisconti et al. (2013: 148).

0= within posterior 50% of anterior process, process is rounded in medial/lateral view

1=within anterior 50% of anterior process, or dorsal and ventral margins parallel

2=within posterior 50% of anterior process, process is triangular in medial/lateral view

181. Periotic, lateral face of anterior process. New character.

0=surface anterior to anterolateral sulcus smooth or smoothly convex

1=distinct tubercle present immediately anterior to anterolateral sulcus and lateral tuberosity

182. Periotic, shape of lateral face of posterior process (if exposed on lateral skull wall). New character.

0=oval or circular

1=triangular

183. Periotic, lateral face of posterior process. New character.

0=convex or tapering

1=flat or concave

184. Periotic, shape of facial and vestibulocochlear canals if separated by crista transversa. New character.

0=circular

1=oval, transversely compressed and anteroposteriorly aligned

185. Periotic, squamosal flange. New character.

0=absent

1=secondary squamosal flange present lateral to fossa for stapedial muscle

186. Periotic, relative size of facial and vestibulocochlear canals. Modified from Ekdale et al. (2011: 40), Fordyce and Marx (2013: 106), El Adli et al. (2014: 97).

0=approximately equal

1=facial canal measures less than or equal to 2/3 transverse diameter of vestibulocochlear canal

187. Periotic, articulation with squamosal. New character.

0=petrosal and squamosal share vertical butt joint

1=lateral projection of falciform process fits into groove on anterior margin of lateral tuberosity

188. Periotic, lateral portion of facial sulcus. New character.

0=distinct or indistinct sulcus

1=sulcus nearly encircled by bone

189. Periotic, thickness of crista transversa. Modified from Bouetel and Muizon (2006: 74), Kimura and Hasegawa (2010: 104), Bisconti et al. (2013: 128, 129), El Adli et al. (2014: 93).

0=anteroposteriorly thin or absent

1=anteroposteriorly as thick or thicker than transverse width of facial canal

2=facial canal and IAM separated by approximately 200% of maximum IAM diameter

190. Periotic, relative size of facial canal and aperture for vestibular aqueduct. Modified from Steeman (2007: 40).

0=facial canal larger than or similar in size to endolymphatic foramen

1=endolymphatic foramen transversely wider than facial canal

191. Periotic, transversely thickest part of body in ventral view. New character.

0=apex of lateral margin positioned posterior to or at level of pars cochlearis

1=apex of lateral margin positioned anterior to or at anterior margin of pars cochlearis

192. Periotic, ventral margin of anterior process. Modified from Bouetel and Muizon (2006: 64), Fitzgerald (2006: 194), Bisconti (2008: 103?), Kimura and Hasegawa (2010: 87), Bisconti et al. (2013: 150).

0=straight or convex in medial view

1=concave, apex of anterior process is ventrally deflected

193. Periotic, lateral surface. Modified from Geisler and Sanders (2003: 216), Fitzgerald (2006: 206).

0=smooth

1=rugose

2=rugose anterior to anterolateral sulcus only

194. Periotic, mallear fossa. Modified from Geisler and Luo (1996: 9), Kimura and Ozawa (2002: 58), Geisler and Sanders (2003: 214), Dooley et al. (2004: 9), Bouetel and Muizon (2006: 78), Fitzgerald (2006: 204), Steeman (2007: 42), Bisconti (2008: 27), Fitzgerald (2010: 86), Kimura and Hasegawa (2010: 96), Ekdale et al. (2011: 43), El Adli et al. (2014: 100).

0=present as distinct pit

1=absent or poorly defined

195. Periotic, ventrolateral ridge. Modified from Geisler and Luo (1996: 16), Kimura and Ozawa (2002: 65), Geisler and Sanders (2003: 215), Dooley et al. (2004: 16), Bouetel and Muizon (2006: 66), Fitzgerald (2006: 205), Fitzgerald (2010: 87).

0=absent

1=present

2=present and expanded

196. Periotic, shape of fenestra rotunda. Modified from Geisler and Sanders (2003: 222), Bouetel and Muizon (2006: 68), Fitzgerald (2006: 212), Fitzgerald (2010: 93), Kimura and Hasegawa (2010: 93).

0=oval

1=teardrop with fissure directed toward aperture for cochlear aqueduct

197. Periotic, caudal tympanic process. Modified from Geisler and Sanders (2003: 225), Bouetel and Muizon (2006: 82), Fitzgerald (2006: 215), Steeman (2007: 32), Fitzgerald (2010: 94), Kimura and Hasegawa (2010: 101), Ekdale et al. (2011: 26), Fordyce and Marx (2013: 98), El Adli et al. (2014: 85).

0=low, semicircular in medial view

1=prominent, forms an acute point in medial view

198. Periotic, caudal tympanic process in posteromedial view. Modified from Kimura and Ozawa (2002: 64), Geisler and Sanders (2003: 226), Dooley et al. (2004: 15), Fitzgerald (2006: 216), Fitzgerald (2010: 95), Kimura and Hasegawa (2010: 100?), Bisconti et al. (2013: 144), Fordyce and Marx (2013: 96), El Adli et al. (2014: 101).

0=separated from crista parotica, no division between stapedial muscle fossa and stylomastoid fossa

1=narrow separation or contact, clear separation of fossae

199. Periotic, fundus of IAM. Modified from Geisler and Luo (1996: 19), Kimura and Ozawa (2002: 68), Geisler and Sanders (2003: 234), Dooley et al. (2004: 19), Bisconti (2005: 37), Bouetel and Muizon (2006: 73), Fitzgerald (2006: 224), Bisconti (2008: 59), Fitzgerald (2010: 102), Kimura and Hasegawa (2010: 103).

0=funnel-shaped

1=tube-shaped

200. Periotic, orientation of caudal tympanic process in ventral view. New character.

0=posteromedially divergent

1=parallel with longitudinal axis of pars cochlearis

201. Periotic, suprameatal area. Modified from Kimura and Ozawa (2002: 67), Dooley et al. (2004: 18), Bouetel and Muizon (2006: 67), Deméré et al. (2008: 50), Ekdale et al. (2011: 33), El Adli et al. (2014: 91).

0=smooth or rugose compact bone

1=distinct pit floored by cancellous bone

2=cancellous bone exposed along dorsal surface of pars cochlearis and suprameatal area

202. Periotic, dorsal surface of pars cochlearis. Modified from Geisler and Luo (1996: 18), Bisconti et al. (2013: 141).

0=dorsal surface is flat or convex and aligned anteroposteriorly

1=pars cochlearis is anterodorsally extended, entire dorsal surface visible in posterior view

203. Periotic, fenestra rotunda. Modified from Steeman (2007: 31?).

0=bone dorsally adjacent to fenestra rotunda is smooth

1=tubercle with transverse sulci and ridges bulging into dorsal margin of fenestra rotunda

204. Periotic, anterior margin of fenestra rotunda.

0=anterior margin is posterior to or at level of posterior margin of fenestra ovalis

1=anterior margin of fenestra rotunda overlaps fenestra ovalis in ventral view

205. Periotic, pinching of IAM. New character.

0=high crista transversa present, or margins of IAM not pinched

1=crista transversa absent and medial and lateral projections from IAM rim pinch the IAM between the facial canal and foramen singulare

206. Periotic, sharp transverse crest between stylomastoid and suprameatal fossae. New character.

0=absent

1=present

2=present as small semicircular ridge ventrally encircling small stylomastoid fossa, suprameatal fossa indistinct

207. Periotic, attachment of anterior process to pars cochlearis in taxa with cranially elongated pars cochlearis. Modified from Ekdale et al. (2011: 19), Fordyce and Marx (2013: 88), El Adli et al. (2014: 79).

0=absent

1=present

208. Periotic, anteroexternal sulcus. Modified from Geisler and Sanders (2003: 205), Bouetel and Muizon (2006: 65), Fitzgerald (2006: 195), Ekdale et al. (2011: 23), Fordyce and Marx (2013: 89).

0=present

1=absent

209. Periotic, distinct ridge delimiting insertion surface of tensor tympani. Modified from Geisler and Luo (1996: 7), Kimura and Ozawa (2002: 56), Geisler and Sanders (2003: 217), Dooley et al. (2004: 7), Bisconti (2005: 82), Deméré et al. (2005: 52), Bouetel and Muizon (2006: 80), Fitzgerald (2006: 207), Bisconti (2008: 148), Deméré et al. (2008: 48), Fitzgerald (2010: 88), Kimura and Hasegawa (2010: 98), Ekdale et al. (2011: 28), Churchill et al. (2012: 72), Bisconti et al. (2013: 138), Fordyce and Marx (2013: 93), El Adli et al. (2014: 87).

0=absent

1=absent, but insertion is excavated

2=present

210. Periotic, promontorial groove. Modified from Geisler and Luo (1996: 13), Kimura and Ozawa (2002: 62), Dooley et al. (2004: 13), Deméré et al. (2005: 53), Bouetel and Muizon (2006: 81), Deméré et al. (2008: 49), Kimura and Hasegawa (2010: 99), Ekdale et al. (2011: 29), Churchill et al. (2012: 70), Fordyce and Marx (2013: 95), El Adli et al. (2014: 88, 137).

0=present

1=present and deeply excavated

2=absent

211. Periotic, anteroposterior alignment of apertures for cochlear and vestibular aqueducts. Modified from Ekdale et al. (2011: 38), Fordyce and Marx (2013: 100).

0=absent

1=present

212. Periotic, path of anterointernal sulcus. Modified from Ekdale et al. (2011: 21), El Adli et al. (2014: 81).

0=sulcus absent

1=directed towards anteroventral angle

2=directed towards anterodorsal angle or ascends dorsally at level of anterior pars cochlearis

213. Periotic, aperture for vestibular aqueduct. New character.

0=opens dorsally

1=forms laterally directed elongate sulcus

214. Periotic, accessory promontorial groove immediately medial to IAM. New character.

0=absent

1=present

215. Periotic, shape of posterior bullar facet. New character.

0=oval or quadrate

1=teardrop shaped, narrowing proximally and broadly rounded distally

2=diamond-shaped, narrowing proximally and distally and widest in middle with medial and lateral apices

216. Periotic, posteromedial corner of pars cochlearis in ventral view. Modified from Geisler and Luo (1996: 21), Kimura and Ozawa (2002: 70), Geisler and Sanders (2003: 228), Dooley et al. (2004: 21), Deméré et al. (2005: 55), Fitzgerald (2006: 218), Bisconti (2008: 92), Deméré et al. (2008: 51), Kimura and Hasegawa (2010: 95), Ekdale et al. (2011: 32), Bisconti et al. (2013: 139), Fordyce and Marx (2013: 97), El Adli et al. (2014: 90).

0=pars cochlearis visible medial to fenestra rotunda

1=dorsally ascending sulcus begins at fenestra rotunda, pars cochlearis not visible

2=as in state 1, but fenestra rotunda is confluent with aperture for cochlear aqueduct or connected by deep sulcus

217. Periotic, stylomastoid fossa. Modified from Geisler and Luo (1996: 14), Kimura and Ozawa (2002: 63), Geisler and Sanders (2003: 224), Dooley et al. (2004: 14), Bisconti (2005: 51), Steeman (2007: 29), Bisconti (2008: 53, 54), Kimura and Hasegawa (2010: 106), Ekdale et al. (2011: 31), Churchill et al. (2012: 75), Bisconti et al. (2013: 143), El Adli et al. (2014: 89).

0=absent or poorly developed

1=developed on much of posterior “base” of pars cochlearis

2=extends distally onto posterior process

218. Periotic, suprameatal fossa. Modified from Bouetel and Muizon (2006: 71), Ekdale et al. (2011: 33, 34), Bisconti et al. (2013: 133), El Adli et al. (2014: 91, 92).

0=deep

1=shallow or absent

219. Periotic, crista transversa and internal acoustic meatus. Modified from Bisconti (2005: 21), Bouetel and Muizon (2006: 72), Bisconti (2008: 65), Kimura and Hasegawa (2010: 102), Ekdale et al. (2011: 35), Churchill et al. (2012: 71), Bisconti et al. (2013: 129), El Adli et al. (2014: 93).

0=crista transversa elevated and easily seen within meatus or elevated to meatal rim

1=crista transversa deeply recessed within meatus or poorly developed

220. Tympanic bulla, orientation of main axis in ventral aspect. Modified from Bouetel and Muizon (2006: 56), Kimura and Hasegawa (2010: 115), Marx (2011: 95), El Adli et al. (2014: 75).

0=parallel

1=diverging posteriorly

2=diverging anteriorly

221. Tympanic bulla, orientation of outer lip. Modified from Bouetel and Muizon (2006: 61), Marx (2011: 96), Fordyce and Marx (2013: 115).

0=faces laterally

1=faces ventrolaterally to ventrally

222. Tympanic bulla, sigmoid fissure curving anteriorly. Modified from Geisler and Sanders (2003: 2593), Fitzgerald (2010: 115), Kimura and Hasegawa (2010: 120), Marx (2011: 97), Fordyce and Marx (2013: 119).

0=present as distinct horizontal crease that curves anteriorly past level of sigmoid process tip

1=absent, present as vertical sigmoid fissure

223. Tympanic bulla, elliptical foramen. Modified from Geisler and Sanders (2003: 261), Fitzgerald (2006: 251), Bisconti (2008: 31), Marx (2011: 98, 99), Bisconti et al. (2013: 165), Fordyce and Marx (2013: 121).

0=present as distinct notch separating two pedicles

1=elliptical foramen absent, single posterior pedicle present

224. Tympanic bulla, medial lobe. Modified from Dooley et al. (2004: 41), Steeman (2007: 44), Ekdale et al. (2011: 5), Marx (2011: 100), Churchill et al. (2012: 61), Bisconti et al. (2013: 173), El Adli et al. (2014: 66).

0=present and distinct from lateral lobe, separated by median furrow on posterior surface

1=present as robust dorsal posterior prominence on posterior surface of involucrum

2=dorsal posterior prominence not developed, medial lobe indistinct

225. Tympanic bulla, eustachian opening in anterior aspect. Modified from Marx (2011: 101), Bisconti et al. (2013: 163).

0=well-developed groove, ventrally depressed below dorsal margin of involucrum

1=above or at level of dorsal margin of involucrum

226. Tympanic bulla, sigmoid process in lateral aspect. Modified from Marx (2011: 102).

0=overlaps anterior half or more of conical process

1=no overlap or slight overlap, gap between sigmoid and apex of conical process

227. Tympanic bulla, transverse ridge dividing tympanic cavity. Modified from Geisler and Sanders (2003: 272), Fitzgerald (2006: 262), Bisconti (2008: 154), Fitzgerald (2010: 118), Marx (2011: 103).

0=present

1=absent

228. Tympanic bulla, ventral surface. Modified from Kimura and Ozawa (2002: 32), Geisler and Sanders (2003: 266), Deméré et al. (2005: 49), Bouetel and Muizon (2006: 59), Fitzgerald (2006: 256), Deméré et al. (2008: 45), Fitzgerald (2010: 116), Kimura and Hasegawa (2010: 118), Ekdale et al. (2011: 4), Marx (2011: 104), Churchill et al. (2012: 59), Bisconti et al. (2013: 166), Fordyce and Marx (2013: 132), El Adli et al. (2014: 65).

0=transversely concave

1=flat or convex

229. Tympanic bulla, medial lobe. Modified from Geisler and Sanders (2003: 252, 267), Fitzgerald (2006: 242, 257), Bisconti (2008: 153), Fordyce and Marx (2013: 124).

0=horizontal crest present on posterior surface

1=crest absent

230. Tympanic bulla, posterior margin in ventral aspect. Modified from Geisler and Sanders (2003: 266), Bouetel and Muizon (2006: 57), Fitzgerald (2006: 256), Steeman (2007: 43), Bisconti (2008: 30), Deméré et al. (2008: 45), Kimura and Hasegawa (2010: 116, 117), Ekdale et al. (2011: 16), Churchill et al. (2012: 59), Fordyce and Marx (2013: 122), El Adli et al. (2014: 76).

0=lateral lobe extends posterior to medial lobe, not separated by notch

1=lateral lobe extends posterior to medial lobe, separated by notch

2=medial and lateral lobes not clearly differentiated

231. Tympanic bulla, involucral ridge. Modified from Geisler and Sanders (2003: 273), Fitzgerald (2006: 263).

0=present as sharp ventromedial ridge

1=low ridge or absent

232. Tympanic bulla, articular facet for basioccipital. New character.

0=present

1=absent

233. Tympanic bulla, inner posterior pedicle. New character.

0=present as thin flange

1=present on swollen tubercle

234. Tympanic bulla, anterior margin in medial view. New character.

0=ventral and dorsal margins form near right angle

1=evenly curved, oval-shaped anterior margin

235. Tympanic bulla, posterior margin of medial lobe in medial view. New character.

0=forms sharp corner, posterior and ventral margins form ≤ 90º angle

1=forms corner or rounded margins, posterior and ventral margins form > 90º angle

236. Tympanic bulla, posterior spur on medial lobe. New character.

0=absent

1=ventral spur present on posteroventral tip of medial lobe

237. Tympanic bulla, position of main ridge in ventral view. Modified from Ekdale et al. (2011: 3), El Adli et al. (2014: 67).

0=main ridge positioned on lateral half of ventral surface

1=main ridge divides medial and lateral sides of bulla

2=main ridge and involucral ridge positioned close to medial edge

238. Tympanic bulla, involucral and main ridges in medial view. Modified from Ekdale et al. (2011: 6), Fordyce and Marx (2013: 117), El Adli et al. (2014: 67).

0=involucral ridge present along ventral margin

1=involucral ridge retracted medially along entire ventral margin

239. Tympanic bulla, anteromedial shelf. Modified from Ekdale et al. (2011: 7), Fordyce and Marx (2013: 134), El Adli et al. (2014: 68, 72).

0=absent

1=vertical, anteriorly directed flange developed

240. Tympanic bulla, anterior lobe in dorsal view. Modified from Bisconti (2008: 44), Fordyce and Marx (2013: 112).

0=convex, continuous with curvature of posterior lobe

1=strongly convex, deviates from curvature of posterior lobe

2=forms right angle, forming rectangular anterior margin of bulla

241. Tympanic bulla, longitudinal furrow lateral to main ridge. New character.

0=absent

1=present

242. Tympanic bulla, proportions. Modified from Kimura and Ozawa (2002: 31), Geisler and Sanders (2003: 251), Bisconti (2005: 34, 36), Deméré et al. (2005: 47, 48), Bouetel and Muizon (2006: 60), Fitzgerald (2006: 241), Steeman (2007: 49), Bisconti (2008: 41), Deméré et al. (2008: 44), Fitzgerald (2010: 113), Kimura and Hasegawa (2010: 119), Ekdale et al. (2011: 2), Churchill et al. (2012: 58), El Adli et al. (2014: 63).

0=wide, width of bulla >65% of length

1=narrow, width of bulla <65% of length

2=very narrow, width of bulla <50% of length

243. Tympanic bulla, involucrum. Modified from Geisler and Sanders (2003: 271), Fitzgerald (2006: 261), Fitzgerald (2010: 117), Bisconti et al. (2013: 171), El Adli et al. (2014: 71).

0=dorsal margin is stepped, divided into narrow anterior part and bulbous posterior part

1=smooth dorsal margin

244. Tympanic bulla, width of medial lobe. Modified from Fordyce and Marx (2013: 126).

0=medial lobe transversely wider than lateral lobe

1=equivalent in width

245. Tympanic bulla, anterior end in dorsal or ventral view. Modified from Fordyce and Marx (2013: 112).

0=pointed or rounded

1=squared

246. Tympanic bulla, position of dorsal origin of lateral furrow. Modified from Ekdale et al. (2011: 14), Fordyce and Marx (2013: 113), El Adli et al. (2014: 74).

0=located along posterior 2/3 of bulla

1=located in anterior 1/3 of bulla

247. Tympanic bulla, orientation of lateral furrow. Modified from Fordyce and Marx (2013: 114).

0=vertical

1=anteroventrally oriented

248. Tympanic bulla, separation of sigmoid process and malleus. Modified from Fordyce and Marx (2013: 118).

0=separated from pedicle of malleus

1=confluent with pedicle of malleus

249. Tympanic bulla, shape of conical process. Modified from Bisconti (2005: 35), Ekdale et al. (2011: 8, 9), Churchill et al. (2012: 62), Fordyce and Marx (2013: 120), El Adli et al. (2014: 69, 70).

0=well developed and dorsally convex

1=reduced to a low ridge or absent

2=developed as robust triangular process

250. Tympanic bulla, orientation of crest on posterior surface of medial lobe. Modified from Fordyce and Marx (2013: 125).

0=horizontal

1=ventromedially sloping

251. Tympanic bulla, anteriormost point of involucral ridge. Modified from Ekdale et al. (2011: 17), Fordyce and Marx (2013: 127), El Adli et al. (2014: 77).

0=extends anteriorly to form the anteriormost point of the bulla

1=in line with posterior or anterior border of the bulla

252. Hyoid, cross section of stylohyal. Modified from Churchill et al. (2012: 91), Fordyce and Marx (2013: 82).

0=cylindrical

1=flattened

253. Hyoid, ankylosed basihyal and thyrohyals. Modified from Deméré et al. (2008: 92), Kimura and Hasegawa (2010: 155), Fordyce and Marx (2013: 83), El Adli et al. (2014: 129).

0=absent

1=present

254. Mandible, medial surface of middle part. Modified from Kimura and Ozawa (2002: 42), Bouetel and Muizon (2006: 91), Bisconti (2008: 94), Deméré et al. (2008: 88), Kimura and Hasegawa (2010: 124), Marx (2011: 105), Fordyce and Marx (2013: 135).

0=flat or convex, similar to lateral surface

1=distinctly flattened relative to lateral surface

255. Mandible, symphysis. Modified from Bisconti (2000: 17), Kimura and Ozawa (2002: 33), Geisler and Sanders (2003: 40), Bisconti (2005: 6), Deméré et al. (2005: 57, 58), Bouetel and Muizon (2006: 88), Fitzgerald (2006: 41), Bisconti (2008: 9), Deméré and Berta (2008: 26), Deméré et al. (2008: 53), Fitzgerald (2010: 26), Kimura and Hasegawa (2010: 121), Marx (2011: 106), Churchill et al. (2012: 82), Bisconti et al. (2013: 174), Fordyce and Marx (2013: 136), El Adli et al. (2014: 104).

0=sutured or fused

1=not sutured

256. Mandible, condyle and neck. Modified from Deméré et al. (2005: 59), Deméré et al. (2008: 55), Marx (2011: 108), Fordyce and Marx (2013: 137), El Adli et al. (2014: 105).

0=forms part of curvature of horizontal ramus

1=medially curved giving mandible sinusoidal profile in dorsal aspect

257. Mandible, curvature of horizontal ramus in dorsal aspect. Modified from Bisconti (2000: 20), Deméré et al. (2005: 61), Fitzgerald (2010: 27), Kimura and Hasegawa (2010: 125), Marx (2011: 109), Churchill et al. (2012: 76), Bisconti et al. (2013: 198), Fordyce and Marx (2013: 138).

0=medially bowed

1=straight or slightly bowed laterally, line connecting anterior and posterior tips stays within body of mandible

2=strongly bowed laterally, line connecting anterior and posterior tips medial to ramus

258. Mandible, anterior extremity relative to middle portion. Modified from Kimura and Ozawa (2002: 39), Bisconti (2005: 46), Bouetel and Muizon (2006: 89), Steeman (2007: 60), Bisconti 2008: 50), Deméré et al. (2008: 89), Kimura and Hasegawa (2010: 122), Marx (2011: 110), Churchill et al. (2012: 78), Bisconti et al. (2013: 190), Fordyce and Marx (2013: 139), El Adli et al. (2014: 109).

0=vertical

1=twisted with the ventral edge shifted medially

2=apex of mandible rotated nearly horizontally

259. Mandible, height of ramus in lateral aspect. Modified from Kimura and Ozawa (2002: 43), Bouetel and Muizon (2006: 90), Bisconti (2008: 95), Fitzgerald (2010: 120), Kimura and Hasegawa (2010: 123), Marx (2011: 111), Churchill et al. (2012: 77), Fordyce and Marx (2013: 140), El Adli et al. (2014: 117).

0=constant height

1=dorsoventrally constricted in middle portion

2=ramus increases in height posteriorly

260. Mandible, mandibular foramen. Modified from Kimura and Ozawa (2002: 38), Deméré et al. (2005: 62), Bouetel and Muizon (2006: 98), Steeman (2007: 58), Bisconti (2008: 26?, 159), Deméré et al. (2008: 58), Fitzgerald (2010: 28), Kimura and Hasegawa (2010: 132), Marx (2011: 112), Bisconti et al. (2013: 193), Fordyce and Marx (2013: 141), El Adli et al. (2014: 107).

0=dorsoventral height approximates that of mandible

1=dorsoventral height about half that of mandible or less

261. Mandible, anterior margin of mandibular foramen. Modified from Bisconti (2008: 160), Marx (2011: 113), Bisconti et al. (2013: 194), Fordyce and Marx (2013: 142).

0=round

1=triangular

262. Mandible, satellite process. Modified from Marx (2011: 114), Bisconti et al. (2013: 181, 182).

0=absent

1=present

263. Mandible, relative position of anterior margin of mandibular foramen. Modified from Bouetel and Muizon (2006: 97), Fitzgerald (2006: 58), Deméré et al. (2008: 87), Kimura and Hasegawa (2010: 129, 130), Marx (2011: 115), Bisconti et al. (2013: 178), Fordyce and Marx (2013: 143), El Adli et al. (2014: 111).

0=anterior to apex of coronoid process

1=at level of tip or posterior edge of coronoid

2=posterior to coronoid process

264. Mandible, subcondylar furrow. Modified from Bisconti (2005: 17), Bouetel and Muizon (2006: 95), Steeman (2007: 59), Deméré et al. (2008: 86), Kimura and Hasegawa (2010: 138), Fordyce and Marx (2013: 144), El Adli et al. (2014: 112).

0=absent

1=only present medially

2=deep groove posteriorly separating condyle and angular process

265. Mandible, coronoid process. Modified from Bisconti (2000: 22), Kimura and Ozawa (2002: 34), Geisler and Sanders (2003: 44), Dooley et al. (2004: 30), Bisconti (2005: 41), Deméré et al. (2005: 64), Bouetel and Muizon (2006: 92), Fitzgerald (2006: 45), Steeman (2007: 56), Bisconti (2008: 32, 121), Deméré and Berta (2008: 27), Deméré et al. (2008: 60), Fitzgerald (2010: 29), Kimura and Hasegawa (2010: 128), Marx (2011: 117), Churchill et al. (2012: 81), Bisconti et al. (2013: 176, 177), Fordyce and Marx (2013: 145), El Adli et al. (2014: 108).

0=anterior margin convex and merging into a horizontal dorsal margin, posterior margin vertical; length of coronoid greater than height

1=anterior and posterior margins parallel, coronoid posterodorsally directed overhanging neck, coronoid higher than long

2=triangular process, anterior and posterior margins convergent, higher than or equal to length

3=triangular process, anteroposteriorly longer than high

266. Mandible, postcoronoid elevation. Modified from Bisconti (2008: 64), Deméré et al. (2008: 82), Kimura and Hasegawa (2010: 131), Marx (2011: 118), Bisconti et al. (2013: 179), Fordyce and Marx (2013: 146).

0=absent

1=present

267. Mandible, articular surface of condyle. Modified from Bisconti (2000: 24), Kimura and Ozawa (2002: 35), Bisconti (2005: 42), Deméré et al. (2005: 63), Bouetel and Muizon (2006: 99), Steeman (2007: 57), Bisconti (2008: 38), Deméré et al. (2008: 59), Fitzgerald (2010: 30), Kimura and Hasegawa (2010: 136), Marx (2011: 119), Churchill et al. (2012: 80), Fordyce and Marx (2013: 148).

0=posterior or posterodorsal

1=directed dorsally, neck and angular process not elevated above ventral margin of mandible

2=directed dorsally, neck and angular process directed dorsoventrally and elevated above ventral margin

268. Mandible, gingival foramina. Modified from Steeman (2007: 61), Marx (2011: 120), Bisconti et al. (2013: 196).

0=absent

1=present

269. Mandible, sulcus for mylohyoid muscle attachment. Modified from Bisconti (2000: 19), Bisconti (2005: 45), Deméré et al. (2005: 60), Bisconti (2008: 49), Deméré et al. (2008: 56), Marx (2011: 121), Churchill et al. (2012: 79), Bisconti et al. (2013: 191), Fordyce and Marx (2013: 149).

0=absent

1=present

270. Mandible, angular process. Modified from Bisconti (2005: 16, 17), Bouetel and Muizon (2006: 94), Kimura and Hasegawa (2010: 134), Bisconti et al. (2013: 186), Fordyce and Marx (2013: 147), El Adli et al. (2014: 118).

0=positioned below condyle

1=projects far posterior to condyle

271. Mandible, mandibular condyle. Modified from Bouetel and Muizon (2006: 101), Steeman (2007: 57), Deméré et al. (2008: 83), Kimura and Hasegawa (2010: 137), Bisconti et al. (2013: 189), El Adli et al. (2014: 115).

0=convex articular surface

1=flattened condyle

272. Mandible, orientation of coronoid process. Modified from Bouetel and Muizon (2006: 92).

0=vertical

1=laterally hooked

273. Mandible, height of condyle. Modified from Geisler and Sanders (2003: 45), Bouetel and Muizon (2006: 10), Fitzgerald (2006: 46).

0=condyle at or slightly above level of ramus anterior to coronoid process

1=dorsal margin of ramus elevated far above condyle

2=condyle elevated far above tip of coronoid process

274. Mandible, orientation of angular process. Modified from Deméré et al. (2008: 84), Kimura and Hasegawa (2010: 135), Bisconti et al. (2013: 186), El Adli et al. (2014: 114).

0=horizontal (posteriorly directed)

1=posteroventrally curved

275. Mandible, longitudinal furrow on dorsomedial surface of proximal ramus. New character.

0=absent

1=present

276. Mandible, apex of mandibular terminus. New character.

0=mandible tapers gradually

1=apex positioned closer to dorsal margin

2=positioned halfway between dorsal and ventral margins

277. Mandible, shape of mandibular terminus. New character.

0=rounded or rectangular in lateral view

1=spear-shaped in lateral view

278. Mandible, symphyseal groove. Modified from Geisler and Sanders (2003: 41), Deméré et al. (2005: 57), Fitzgerald (2006: 42), Bisconti et al. (2013: 175).

0=absent in adults

1=prominent in adults

279. Mandible, angular process. Modified from Bisconti (2005: 16), Bouetel and Muizon (2006: 93), Bisconti (2008: 84), Kimura and Hasegawa (2010: 133), Bisconti et al. (2013: 187).

0=dorsoventrally flattened and flange-like

1=developed as a swollen tubercle

280. Mandible, groove for insertion for internal pterygoid muscle. Modified from Bouetel and Muizon (2006: 96), Bisconti et al. (2013: 183, 184).

0=absent

1=shallow groove present along medial edge of mandibular foramen

281. Dentition, teeth. Modified from Bisconti (2000: 6, 18), Bisconti (2005: 11), Deméré et al. (2005: 65, 66), Steeman (2007: 1), Bisconti (2008: 15), Deméré and Berta (2008: 42), Deméré et al. (2008: 61), Fitzgerald (2010: 17, 25), Kimura and Hasegawa (2010: 28), Marx (2011: 25), Churchill et al. (2012: 83), Bisconti et al. (2013: 36, 173), Fordyce and Marx (2013: 22), El Adli et al. (2014: 119).

0=present in adult

1=absent in adult

282. Dentition, orientation of accessory cusps on posterior cheek tooth. New character.

0=parallel with central cusp

1=cusps arranged radially

283. Dentition, extent of division between roots. Modified from Fitzgerald (2010: 123).

0=extends past basalmost enamel of crown

1=does not pass enamel of crown, or does not emerge from alveolus

284. Dentition, upper anterior incisors. Modified from Deméré et al. (2008: 100).

0=procumbent

1=vertical

285. Dentition, lower anterior incisors. Modified from Geisler and Sanders (2003: 35), Fitzgerald (2006: (36), Deméré et al. (2008: 100).

0=procumbent

1=vertical

286. Dentition, number of double rooted teeth in maxilla. Modified from Geisler and Sanders (2003: 23), Fitzgerald (2006: 24), Deméré and Berta (2008: 20, 21), Deméré et al. (2008: 99), Fitzgerald (2010: 16), El Adli et al. (2014: 122).

0=5 or more

1=1 or 2

2=none

287. Dentition, diastemata. Modified from Geisler and Sanders (2003: 25), Fitzgerald (2006: 26), Deméré and Berta (2008: 24), Fitzgerald (2010: 18).

0=diastemata absent

1=wide diastemata between cheek teeth

288. Dentition, longitudinal fluting on postcanine teeth. Modified from Geisler and Sanders (2003: 26), Fitzgerald (2006: 27), Deméré and Berta (2008: 22), Deméré et al. (2008: 101), Fitzgerald (2010: 19), El Adli et al. (2014: 121).

0=on lingual surface only

1=well developed lingually and labially

2=poorly developed or absent

289. Dentition, shape of posterior cheek teeth. Modified from Geisler and Sanders (2003: 30), Fitzgerald (2006: 31), Fitzgerald (2010: 20).

0=high, peg-like teeth, crown base is <37% of crown height

1=equilaterally triangular in shape, approximately as high as anteroposteriorly long

2=wide, low teeth, crown base is >120% of crown height

290. Dentition, labial cingulum on upper cheek teeth. Modified from Geisler and Sanders (2003: 31), Fitzgerald (2006: 32), Fitzgerald (2010: 21).

0=present

1=absent

291. Dentition, lingual cingulum on upper cheek teeth. Modified from Geisler and Sanders (2003: 32), Fitzgerald (2006: 33), Fitzgerald (2010: 22).

0=present

1=absent

292. Dentition, accessory denticles on posterior cheek teeth. Modified from Geisler and Sanders (2003: 33), Deméré et al. (2005: 66), Fitzgerald (2006: 34), Deméré and Berta (2008: 23), Deméré et al. (2008: 102), Fitzgerald (2010: 23), El Adli et al. (2014: 120).

0=absent

1=present

293. Dentition, central denticle. Modified from Geisler and Sanders (2003: 34), Fitzgerald (2006: 35), Fitzgerald (2010: 24).

0=larger than accessory denticles

1=subequal

294. Dentition, posteriormost upper tooth. Modified from Fitzgerald (2010: 122).

0=at level of or posterior to antorbital notch

1=anterior to antorbital notch

295. Dentition, heterodonty. New character.

0=present, incisors, canine, and anterior cheek teeth incisiform and posterior cheek teeth are multicuspate

1=homodont, all teeth are incisiform

296. Cervical vertebrae, fusion. Modified from Bisconti (2005: 39), Deméré et al. (2005: 67), Kimura and Hasegawa (2010: 139), Marx (2011: 124), Churchill et al. (2012: 93), Fordyce and Marx (2013: 152), Bisconti et al. (2013: 200, 216), El Adli et al. (2014: 124).

0=separate

1=partially fused

2=fused

297. Atlas and Axis, ventral tubercle/hypophysis. New character.

0=present

1=absent

298. Atlas, base of transverse process. Modified from Marx (2011: 122), Fordyce and Marx (2013: 150).

0=greater than half height of articular surface, process has dorsal and ventral processes

1=less than or equal to half height of articular surface, rectangular or triangular, anteroposteriorly thin

2=greater than half height of articular surface, process is rectangular and anteroposteriorly thick

299. Atlas, shape of neural canal. New character.

0=circular or rectangular, approximately as wide as high

1=circular or rectangular, transverse width approximately 2/3 of dorsoventral height

2=figure-8 shaped

300. Atlas, dorsoventral thickness of neural canal. New character.

0=large, neural canal constitutes more than 2/3 of dorsoventral height of arch and body

1=small, neural canal constitutes 2/3 or less of dorsoventral height of arch and body

301. Atlas, neural arch in dorsal view. New character.

0=arch anteroposteriorly shorter than body, retracted from anterior margin or from both anterior and posterior margins

1=arch anteroposteriorly shorter than body, positioned anteriorly

2=arch equivalent to anteroposterior length of body

302. Atlas, neural spine. New character.

0=present and small, less than dorsoventral thickness of medial part of arch

1=present and tall, higher than dorsoventral thickness of medial part of arch

2=absent

303. Axis, transverse foramen. Modified from Marx (2011: 123), Fordyce and Marx (2013: 151).

0=present

1=absent

304. Axis, transverse width relative to atlas. Modified from Steeman (2007: 65), Kimura and Hasegawa (2010: 141).

0=less than or equal to width of atlas

1=much wider than atlas

305. Axis, shape of neural canal. New character.

0=oval, rectangular, or triangular, flat ventral margin

1=dorsally convex ventral margin giving canal crescent-shape

306. Axis, anterior projection of neural spine. New character.

0=present, extends far anterior to anterior articular surface

1=absent or reduced, does not extend anteriorly past articular surface

307. Axis, lateral margin of pedicle. New character.

0=postzygapophysis extends dorsolaterally, visible as thickening of arch in anterior view

1=postzygapophysis extends posteriorly, not visible in anterior view

308. Axis, ventral margin of body (excluding tubercle/hypophysis and transverse process) . New character.

0=straight or convex

1=ventrally concave

309. Axis, dorsoventral height of neural foramen. New character.

0=less than 2/3 height of anterior articular surface

1=greater than or equal to 2/3 height of anterior articular surface

310. C3-C6, size of lateral vertebral canals. Modified from Bisconti (2013: 211, 212, 213, 214, 215).

0=small, less than dorsoventral thickness of ventral transverse process

1=large, much larger than dorsoventral thickness of ventral transverse process

311. C4-C7, dimensions of neural canal. New character.

0=approximately as transversely wide as dorsoventrally high

1=transversely wide, greater than twice the dorsoventral height

312. C3-C7, shape of neural canal. New character.

0=oval, rectangular, or triangular, flat ventral margin

1=dorsally convex ventral margin giving canal crescent-shape

313. C7, position of transverse process. New character.

0=ventral margin positioned within ventral 50% of body

1=ventral margin positioned within dorsal 50% of body or higher

314. C7, parapophysis. Modified from Fordyce and Marx (2013: 153).

0=present

1=absent

315. Lumbar vertebrae, transverse process. Modified from Geisler and Sanders (2003: 284), Churchill et al. (2012: 96), Fordyce and Marx (2013: 155).

0=ventrolaterally directed

1=laterally directed

316. Lumbar vertebrae, shape of transverse process. Modified from Geisler and Sanders (2003: 285), Fordyce and Marx (2013: 155).

0=slender and transversely wider than anteroposteriorly long

1=shaped like a broad plate and equidimensional

2=wider than long with distinct projection on anterior margin

317. Sternum, composition. Modified from Bisconti (2005: 9), Kimura and Hasegawa (2010: 154), Marx (2011: 125), Bisconti et al. (2013: 246), Fordyce and Marx (2013: 156), El Adli et al. (2014: 127).

0=composed of several bones

1=single element

318. Sternum, articulation with ribs. Modified from Bisconti (2005: 10), Marx (2011: 126), Bisconti et al. (2013: 235).

0=several ribs articulate with sternum

1=single pair articulates

319. Scapula, coracoid process. Modified from Kimura and Ozawa (2002: 48), Geisler and Sanders (2003: 292), Bisconti (2005: 76), Deméré et al. (2005: 69), Kimura and Hasegawa (2010: 146), Marx (2011: 127), Churchill et al. (2012: 99), Bisconti et al. (2013: 222), Fordyce and Marx (2013: 158).

0=present

1=absent

320. Scapula, acromion process. Modified from Bisconti (2005: 75), Deméré et al. (2005: 68), Kimura and Hasegawa (2010: 145), Marx (2011: 128), Churchill et al. (2012: 98), Bisconti et al. (2013: 221), Fordyce and Marx (2013: 159).

0=present

1=absent

321. Scapula, shape. Modified from Bisconti (2005: 77), Bisconti (2008: 68), Kimura and Hasegawa (2010: 147), Fordyce and Marx (2013: 157).

0=anteroposterior length equivalent to proximodistal length

1=anteroposteriorly longer than proximodistal length

322. Scapula, supraspinous fossa. Modified from Geisler and Sanders (2003: 293), Bisconti et al. (2013: 219), Fordyce and Marx (2013: 160).

0=present

1=absent or nearly absent with acromion located near anterior edge of scapula

323. Humerus, length. Modified from Kimura and Ozawa (2002: 50), Geisler and Sanders (2003: 297), Dooley et al. (2004: 40), Bisconti (2005: 78), Deméré et al. (2005: 70), Bisconti (2008: 96), Deméré et al. (2008: 66), Kimura and Hasegawa (2010: 150), Marx (2011: 129), Churchill et al. (2012: 100), Bisconti et al. (2013: 224), Fordyce and Marx (2013: 162), El Adli et al. (2014: 125).

0=longer than radius/ulna

1=same length

2=shorter than radius/ulna

324. Humerus, deltopectoral crest. Modified from Geisler and Sanders (2003: 294), Kimura and Hasegawa (2010: 148), Churchill et al. (2012: 102), Bisconti et al. (2013: 225), Fordyce and Marx (2013: 161).

0=present as distinct crest

1=absent or reduced to a variably developed rugosity

325. Humerus, humeral head in lateral or medial view. Modified from Churchill et al. (2012: 101), Fordyce and Marx (2013: 163).

0=angled

1=vertical

326. Humerus, distal end. Modified from Churchill et al. (2012: 103), Fordyce and Marx (2013: 164).

0=distal trochlea is narrower than distal shaft

1=distal trochlea is equal to or wider than distal shaft

327. Humerus, head. New character.

0=small, maximum diameter less than 1/3 length of humerus

1=large, maximum diameter approximately 1/2 length of humerus

328. Humerus, position of apex of deltopectoral crest. New character.

0=positioned on distal 1/2 of humerus

1=positioned on proximal 1/2 of humerus

329. Humerus, lesser tuberosity. New character.

0=present

1=absent

330. Humerus, distal trochlea. Modified from Bisconti et al. (2013: 226).

0=single convex articular surface for ulna and radius

1=developed as two oval-rectangular planar facets

331. Radius, curvature. Modified from Bisconti (2005: 79).

0=anteriorly bowed

1=straight

332. Radius, proximal end in lateral view. New character.

0=proximally narrow and abruptly widens in proximal 1/4 of shaft

1=widest proximally, tapers distally

2=roughly parallel anterior and posterior margins

333. Radius, length relative to ulna. New character.

0=shorter than ulna

1=equal to or longer than ulna

334. Radius and Ulna, shaft (excluding olecranon process). New character.

0=ulna anteroposteriorly wider than radius

1=ulna anteroposteriorly equal to or narrower than radius

335. Ulna, olecranon process. Modified from Geisler and Sanders (2003: 298), Bisconti (2005: 74), Marx (2011: 130), Bisconti et al. (2013: 227), Fordyce and Marx (2013: 165).

0=present

1=absent

336. Ulna, distal 1/2 of shaft. New character.

0=widens distally

1=parallel margins, rectangular shaft

337. Ulna, olecranon process. Modified from Churchill et al. (2012: 104).

0=extends proximal to humeral articular surface

1=does not extend proximal to humeral articular surface

338. Ulna, distal olecranon process. New character.

0=concave posterior margin, smoothly transitions to shaft

1=distinct notch present between olecranon and shaft

339. Manus, number of digits. Modified from Kimura and Ozawa (2002: 49), Geisler and Sanders (2003: 299), Dooley et al. (2004: 35), Deméré et al. (2005: 71), Bouetel and Muizon (2006: 70), Deméré et al. (2008: 67), Kimura and Hasegawa (2010: 151), Marx (2011: 131), Churchill et al. (2012: 106), Bisconti et al. (2014: 229, 230), El Adli et al. (2014: 126).

0=pentadactyl

1=tetradactyl

340. Soft tissue, ventral throat grooves. Modified from Bisconti (2000: 75), Geisler and Sanders (2003: 301), Deméré et al. (2005: 73), Bisconti (2008: 52), Deméré et al. (2008: 69), Kimura and Hasegawa (2010: 159), Marx (2011: 132), Churchill et al. (2012: 112).

0=absent or few (2-10) confined to throat region

1=numerous and terminate midbody

2=numerous and extend to or posterior to umbilicus

341. Soft tissue, ventral throat pouch. Modified from Deméré et al. (2005: 74), Deméré et al. (2008: 70), Kimura and Hasegawa (2010: 160), Marx (2011: 133), Churchill et al. (2012: 110).

0=absent

1=present

342. Soft tissue, tongue. Modified from Deméré et al. (2005: 79), Deméré et al. (2008: 75), Kimura and Hasegawa (2010: 161), Marx (2011: 134), Churchill et al. (2012: 109).

0=muscular

1=reduced and predominantly connective tissue

343. Soft tissue, temporomandibular joint. Modified from Marx (2011: 135).

0=synovial

1=non-synovial, fibrocartilaginous pad

344. Soft tissue, longitudinal ridges on rostrum. Modified from Deméré et al. (2005: 85), Deméré et al. (2008: 76), Kimura and Hasegawa (2010: 158), Marx (2011: 136).

0=absent or indistinct

1=single median ridge

2=three longitudinal ridges

345. Soft tissue, dorsal fin. Modified from Geisler and Sanders (2003: 304), Deméré et al. (2005: 72), Bisconti (2008: 51), Deméré et al. (2008: 68), Marx (2011: 137), Churchill et al. (2012: 115).

0=present as fin or dorsal hump

1=absent

346. Soft tissue, baleen. Modified from Bisconti (2000: 16), Kimura and Ozawa (2002: 30), Geisler and Sanders (2003: 1), Bisconti (2005: 13), Deméré et al. (2005: 75), Fitzgerald (2006: 1), Steeman (2007: 1), Bisconti (2008: 17), Deméré et al. (2008: 71), Marx (2011: 138), Bisconti et al. (2013: 13).

0=absent

1=present

347. Soft tissue, profile of mouth in lateral view. New character.

0=straight or dorsally concave

1=mostly straight and downturned near eye

2=dorsally arched and evenly curved

348. Soft tissue, number of baleen plates. Modified from Deméré et al. (2005: 75), Churchill et al. (2012: 86).

0=fewer than 200

1=200 to 270

2=greater than 270

349. Soft tissue, length of baleen plates. Modified from Bisconti (2005: 54), Bisconti (2008: 39), Kimura and Hasegawa (2010: 163), Bisconti et al. (2013: 14).

0=shorter than ½ length of rostrum

1=longer than ½ length of rostrum

350. Soft tissue, subrostral gap. Modified from Deméré et al. (2005: 76), Churchill et al. (2012: 87).

0=absent, baleen racks converge at midline

1=present, baleen racks separated by gap

351. Color patterns, rostral saddle. Modified from Arnold et al. (2005: 1), Marx (2011: 139).

0=absent

1=present

2=well developed

352. Color patterns, blowhole streaks. Modified from Arnold et al. (2005: 2), Marx (2011: 140).

0=absent

1=present

2=well developed

353. Color patterns, dark nape field. Modified from Arnold et al. (2005: 3), Marx (2011: 141).

0=absent

1=dark nape present

2=nape light

354. Color patterns, shape of dorsal nape field. Modified from Arnold et al. (2005: 4), Marx (2011: 142).

0=nape streak absent

1=V-shaped chevron

2=nape blaze linear to diffuse

355. Color patterns, ventral nape streak. Modified from Arnold et al. (2005: 5), Marx (2011: 143).

0=absent

1=present

356. Color patterns, ear stripe. Modified from Arnold et al. (2005: 6), Marx (2011: 144).

0=absent

1=present

357. Color patterns, basal flipper color. Modified from Arnold et al. (2005: 7), Deméré et al. (2005: 81), Marx (2011: 145).

0=uniformly colored

1=uniform with white leading edge

2=white

3=dark

358. Color patterns, distal flipper color. Modified from Arnold et al. (2005: 8), Marx (2011: 146).

0=uniformly colored

1=uniform with light leading edge

2=dark grey

359. Color patterns, axillary patch. Modified from Arnold et al. (2005: 9), Marx (2011: 147).

0=absent

1=present

360. Color patterns, thorax field. Modified from Arnold et al. (2005: 10), Marx (2011: 148).

0=body uniformly colored

1=dark dorsum and lighter ventrum, no intermediate gray

2=light gray transitional field present

361. Color patterns, caudal chevron. Modified from Arnold et al. (2005: 11), Marx (2011: 149).

0=absent

1=single chevron

2=double chevron

362. Color patterns, asymmetry. Modified from Arnold et al. (2005: 13), Deméré et al. (2005: 82), Marx (2011: 150).

0=absent

1=present but subtle

2=well developed

363. Color patterns, baleen. Modified from Churchill et al. (2012: 89).

0=uniformly light

1=uniformly black or dark grey

2=dark and light, asymmetrical pigmentation

**References**

**Arnold PW, Birtles RA, Dunstan A, Lukoschek V, Matthews M. 2005.** Colour patterns of the dwarf minke whale *Balaenoptera acutorostrata sensu lato*: description, phylogenetic analysis, and taxonomic implications. *Memoirs of the Queensland Museum* **51:** 277-307.

**Bisconti M. 2000.** New description, character analysis and preliminary phyletic assessment of two Balaenidae skulls from the Italian Pliocene. *Palaeontographia Italica* **87:** 37-66.

**Bisconti M. 2005.** Skull morphology and phylogenetic relationships of a new diminutive balaenid from the lower Pliocene of Belgium. *Palaeontology* **48:** 793-816.

**Bisconti M. 2008.** Morphology and phylogenetic relationships of a new eschrichtiid genus (Cetacea: Mysticeti) from the early Pliocene of northern Italy. *Zoological Journal of the Linnean Society* **153:** 161-186.

**Bisconti M, Lambert O, Bosselaers M. 2013.** Taxonomic revision of *Isocetus depauwi* (Mammalia, Cetacea, Mysticeti) and the phylogenetic relationships of archaic 'cetothere' mysticetes. *Palaeontology* **56:** 95-127.

**Bouetel V, Muizon C de . 2006.** The anatomy and relationships of *Piscobalaena nana* (Cetacea, Mysticeti), a Cetotheriidae s.s. from the early Pliocene of Peru. *Geodiversitas* **28:** 319-395.

**Churchill M, Berta A, Deméré TA. 2012.** The systematics of right whales (Mysticeti: Balaenidae). *Marine Mammal Science* **28:** 497-521.

**Deméré TA, Berta A. 2008.** Skull anatomy of the Oligocene toothed mysticete *Aetiocetus weltoni* (Mammalia; Cetacea): implications for mysticete evolution and functional anatomy. *Zoological Journal of the Linnaean Society* **154:** 308-352.

**Deméré TA, Berta A, McGowen MR. 2005.** The taxonomic and evolutionary history of modern balaenopteroid mysticetes. *Journal of Mammalian Evolution* **12:** 99-143.

**Deméré TA, McGowen MR, Berta A, Gatesy J. 2008.** Morphological and molecular evidence for a stepwise evolutionary transition from teeth to baleen in mysticete whales. *Systematic Biology* **57:** 15-37.

**Dooley AC, Fraser NS, Luo Z. 2004.** The earliest known member of the rorqual-gray whale clade (Mammalia, Cetacea). *Journal of Vertebrate Paleontology* **24:** 453-463.

**Ekdale EG, Berta A, Deméré TA. 2011.** The comparative osteology of the petrotympanic complex (ear region) of extant baleen whales (Cetacea: Mysticeti). *PLoS ONE* **6:** 1-42.

**El Adli JJ, Deméré TA, Boessenecker RW. 2014.** *Herpetocetus morrowi* (Cetacea:Mysticeti) a new species of diminutive baleen whale from the Upper Pliocene (Piacenzian) of California, USA, with observations on the evolution and relationships of the Cetotheriidae. *Zoological Journal of the Linnaean Society* **170:** 400-466.

**Fitzgerald EMG. 2006.** A bizarre new toothed mysticete (Cetacea) from Australia and the early evolution of baleen whales. *Proceedings of the Royal Society B* **273:** 2955-2963.

**Fitzgerald EMG. 2010.** The morphology and systematics of *Mammalodon colliveri* (Cetacea: Mysticeti), a toothed mysticete from the Oligocene of Australia. *Zoological Journal of the Linnaean Society* **158:** 367-476.

**Fordyce RE, Marx FG. 2013.** The pygmy right whale *Caperea marginata*: the last of the cetotheres. *Proceedings of the Royal Society B* **280:** 20122645.

**Geisler JH, A.E. S. 2003.** Morphological evidence for the phylogeny of Cetacea. *Journal of Mammalian Evolution* **10:** 23-129.

**Geisler JH, Luo Z. 1996.** The petrosal and inner ear of *Herpetocetus* sp. (Mammalia: Cetacea) and their implications for the phylogeny and hearing of archaic mysticetes. *Journal of Paleontology* **70:** 1045-1066.

**Kimura T, Hasegawa Y. 2010.** A new baleen whale (Mysticeti: Cetotheriidae) from the earliest late Miocene of Japan and a reconsideration of the phylogeny of cetotheres. *Journal of Vertebrate Paleontology* **30:** 577-591.

**Kimura T, Ozawa T. 2002.** A new cetothere (Cetacea: Mysticeti) from the early Miocene of Japan. *Journal of Vertebrate Paleontology* **22:** 684-702.

**Marx FG. 2011.** The more the merrier? A large cladistic analysis of mysticetes, and comments on the transition from teeth to baleen. *Journal of Mammalian Evolution* **18:** 77-100.

**Steeman ME. 2007.** Cladistic analysis and a revised classification of fossil and recent mysticetes. *Zoological Journal of the Linnean Society* **150:** 875-894.
